# Smoking, nicotine and pregnancy 2 (SNAP2) trial: protocol for a randomised controlled trial of an intervention to improve adherence to nicotine replacement therapy during pregnancy

**DOI:** 10.1136/bmjopen-2024-087175

**Published:** 2024-05-28

**Authors:** Miranda M Clark, Sue Cooper, Felix Naughton, Michael Ussher, Joanne Emery, Lisa McDaid, Ross Thomson, Lucy Phillips, Linda Bauld, Paul Aveyard, David Torgerson, Ivan Berlin, Sarah Lewis, Steve Parrott, Catherine Hewitt, Charlie Welch, Gill Parkinson, Anne Dickinson, Stephen Sutton, James Brimicombe, Katharine Bowker, Andrew McEwen, Kavita Vedhara, Tim Coleman

**Affiliations:** 1 Centre for Academic Primary Care, School of Medicine, University of Nottingham, Nottingham, UK; 2 Addiction Research Group, School of Health Sciences, University of East Anglia, Norwich, UK; 3 Population Health Research Institute, St George's University of London, London, UK; 4 Institute of Social Marketing and Health, University of Stirling, Stirling, UK; 5 Usher Institute and Behavioural Research UK, The University of Edinburgh, Edinburgh, UK; 6 Nuffield Department of Primary Care Health Sciences, University of Oxford, Oxford, UK; 7 York Trials Unit, Department of Health Sciences, University of York, York, UK; 8 Department of Medical Pharmacology, Pitié Salpêtrière Hospital-Sorbonne Université, Paris, France; 9 School of Medicine, University of Nottingham, Nottingham, UK; 10 Department of Health Sciences, University of York, York, UK; 11 Behavioural Science Group, University of Cambridge, Cambridge, UK; 12 Cambridge Research Methods Hub, University of Cambridge, Cambridge, UK; 13 National Centre for Smoking Cessation and Training (NCSCT), Dorchester, UK; 14 Department of Behavioural Science and Health, University College London, London, UK; 15 School of Psychology, Cardiff University, Cardiff, UK

**Keywords:** public health, smoking reduction, maternal medicine, respiratory medicine (see thoracic medicine)

## Abstract

**Introduction:**

Smoking during pregnancy is harmful to unborn babies, infants and women. Nicotine replacement therapy (NRT) is offered as the usual stop-smoking support in the UK. However, this is often used in insufficient doses, intermittently or for too short a time to be effective. This randomised controlled trial (RCT) explores whether a bespoke intervention, delivered in pregnancy, improves adherence to NRT and is effective and cost-effective for promoting smoking cessation.

**Methods and analysis:**

A two-arm parallel-group RCT was conducted for pregnant women aged ≥16 years and who smoke ≥1 daily cigarette (pre-pregnancy smoked ≥5) and who agree to use NRT in an attempt to quit. Recruitment is from antenatal care settings and via social media adverts. Participants are randomised using blocked randomisation with varying block sizes, stratified by gestational age (<14 or ≥14 weeks) to receive: (1) usual care (UC) for stop smoking support or (2) UC plus an intervention to increase adherence to NRT, called ‘Baby, Me and NRT’ (BMN), comprising adherence counselling, automated tailored text messages, a leaflet and website. The primary outcome is biochemically validated smoking abstinence at or around childbirth, measured from 36 weeks gestation. Secondary outcomes include NRT adherence, other smoking measures and birth outcomes. Questionnaires collect follow-up data augmented by medical record information. We anticipate quit rates of 10% and 16% in the control and intervention groups, respectively (risk ratio=1.6). By recruiting 1320 participants, the trial should have 90% power (alpha=5%) to detect this intervention effect. An economic analysis will use the Economics of Smoking in Pregnancy model to determine cost-effectiveness.

**Ethics and dissemination:**

Ethics approval was granted by Bloomsbury National Health Service’s Research Ethics Committee (21/LO/0123). Written informed consent will be obtained from all participants. Findings will be disseminated to the public, funders, relevant practice/policy representatives, researchers and participants.

**Trial registration number:**

ISRCTN16830506.

**Protocol version:**

5.0, 10 Oct 2023.

**STRENGTHS AND LIMITATIONS OF THIS STUDY**

- This randomised controlled trial (RCT) is testing an intervention aimed at addressing women’s concerns about and barriers to nicotine replacement therapy (NRT) adherence.
- The trial design is explanatory and pragmatic and thereby will show whether changes in smoking are due to altered adherence to NRT.
- We report the design of the RCT according to the Standard Protocol Items: Recommendations for Interventional Trial guidelines.
- Participants are not blind to the treatments, and this could cause bias, which is limited by using biochemical verification of abstinence as the primary outcome.
- Obtaining data on smoking abstinence from pregnant people who smoke is difficult; using routine data may ameliorate this.

## Introduction

Smoking in pregnancy is still a prevalent public health issue worldwide. For example in the UK, around 7.5% of UK women smoke during childbirth.^1^ Smoking in pregnancy is associated with preventable negative outcomes for both women and babies, and women who stop smoking during pregnancy are less likely to have premature or low-birth-weight infants.^2^ Compared with mothers who do not smoke, those who continue smoking in pregnancy have heightened risks of placental abruption, miscarriage, stillbirth and ectopic pregnancy.^3^ Children born to parents who smoke are more likely to start smoking themselves,^4^ and tobacco smoking is a major risk factor for six of the eight leading causes of death worldwide.^5^

Pregnancy is probably a life event that most motivates people to try to quit smoking; around 50% of women who smoke stop during gestation; and many others try but fail.^6^ A key reason is that nicotine withdrawal symptoms and smoking urges experienced while trying to stop smoking are difficult to tolerate. Nicotine replacement therapy (NRT) provides nicotine without exposing users to toxins like tar, cyanide and carbon monoxide (CO) and, thereby, safely helps ameliorate withdrawal and smoking urges. In the UK, the National Institute for Health and Care Excellence recommended NRT use in pregnancy since 2010^7 8^ and is now a central component of routine clinical practice.^9–11^ However, although NRT is effective in general,^12^ it appears to work less well in pregnancy,^13^ probably because pregnant women do not use it consistently, long enough or in sufficient doses. In trials enrolling pregnant smokers, only 7% to 30% finished courses of NRT,^13^ and of pregnant smokers prescribed NRT by UK general practitioners (GPs), only 30% were supplied it for longer than 2 weeks,^11^ and such short NRT courses are ineffective. In contrast, non-pregnant smokers enrolled in smoking cessation trials report up to 94% adherence levels.^14^

It is very likely that for NRT to work in pregnancy, higher nicotine doses and dosing consistency are needed than are currently used. In pregnancy nicotine metabolism accelerates,^15 16^ resulting in NRT generating lower blood nicotine concentrations. Additionally, research suggests that many pregnant women struggle to use NRT consistently or for sufficiently long due to concerns about nicotine safety and a lack of belief in the need for NRT to quit smoking. These are influenced by erroneous lay beliefs contributing to idiosyncratic NRT usage patterns and NRT not being used as advised.^17 18^ Such erratic NRT use can be compounded by inaccurate advice on nicotine safety from friends, family and even health professionals, exacerbating women’s uncertainties about whether and how to use NRT.^19^ Both stop-smoking practitioners (SSPs)^20^ and pregnant women^21^ believe that by consistently countering such misinformation, improvements could be made in the number of successful quit attempts. Poor adherence to, and intermittent use of, NRT very likely reduces the chances of smoking cessation in pregnancy, limiting the health benefits that could accrue from the optimal use of this treatment.

If better adherence to NRT is not more harmful to the fetus than smoking and helps more pregnancies become smoke-free while avoiding smoking-related harms, encouraging adherence to NRT would clearly be ethical. There is no biological rationale for suspecting that NRT could be more harmful than smoking in pregnancy. Throughout the 2000s, based on the logical belief that smoking-related harms in pregnancy are unlikely to be due solely to nicotine, there has been expert consensus for using NRT to stop smoking in pregnancy.^22^ NRT in pregnancy is not recommended for ‘never smokers’, but NRT used *instead* of smoking is very likely to be safer. In the unlikely event of the occurrence of unexpected nicotine-attributable fetal harm(s), one would expect these to be vastly outweighed by benefits from smoking cessation following an effective NRT use. A Cochrane review found no evidence that, for pregnancy outcomes, NRT harms either women or their babies, although analyses were generally underpowered to detect moderate-sized effects^13^ and observational studies are not sufficiently robust to add to these findings.^23^ However, compared with smoking, NRT has an apparently protective effect on infant development; at 2 years old, infants in the largest RCT of NRT in pregnancy,^24^ born to women randomised to NRT rather than placebo, were more likely to have unimpaired development.^25^

### Rationale

In a National Institute for Health Research-funded programme, we developed BMN, an intervention to improve adherence to NRT during pregnancy.^26^ In cohort studies, we optimised and monitored the impacts of BMN, and this RCT explores whether BMN helps pregnant women stop smoking and increases adherence to NRT. In this paper, we report the protocol of SNAP2 according to the Standard Protocol Items: Recommendations for Interventional Trial guidelines.^27^

## Methods and analysis

SNAP2 is a multi-centre, parallel-group, individually randomised controlled trial (RCT) of the BMN intervention integrated with usual smoking cessation support during pregnancy versus usual smoking cessation support alone.

This RCT was originally envisaged solely as a ‘proof-of-concept’ study that aimed to detect whether BMN increased NRT adherence. If so, a separate RCT was planned to explore BMN effects on cessation. However, due to National Health Service (NHS) service provision changes and the COVID-19 pandemic, the funder accepted that following the demonstration of ‘proof-of-concept’ from a pilot phase, efficacy could be tested by recruiting sufficient participants to SNAP2. Below, we indicate that methodological features were used only in the pilot, and the sample size section explains the basis for the progression from pilot to full trial.

### Objectives

#### Primary objective

To determine whether, when added to the usual NHS cessation support, the BMN intervention increases smoking abstinence during pregnancy, as measured in late pregnancy or at childbirth, with exhaled CO and/or saliva samples used to validate self-reported abstinence.

#### Secondary objectives

In *all* participants, to compare between the intervention and usual care groups:

1. Reported smoking abstinence at 28 days after the quit date (QD).
2. Reported smoking abstinence at both 28 days after a QD, and in late pregnancy/childbirth with and without validation in late pregnancy.
3. The number of days of NRT use in the first 28 days after the QD.
4. Mean daily nicotine dose in the first 7 days after the QD (‘intensity’ of the NRT use).
5. Adverse pregnancy outcome rates.

In the *intervention group* participants:

1. To assess engagement with BMN intervention components.

*Economic*

1. To investigate the cost-effectiveness of the BMN intervention.

#### Pilot phase objectives

In *all* pilot phase participants, the intervention and usual care groups were compared:

1. Urges to smoke and tobacco withdrawal symptoms on Day 7 after the QD.
2. NRT concerns and necessity beliefs on Day 28.

In the pilot phase, participants and practitioners in the *intervention group* only:

1. To assess the fidelity of the BMN intervention delivery.

#### Other objectives (pilot phase only)

To compare biochemically measured nicotine exposure before and after exposure to BMN intervention

### Participants and setting

#### Inclusion criteria

People are eligible if aged ≥16 years; pregnant and <25 weeks of gestation; they smoked ≥5 daily cigarettes before pregnancy (currently smoking ≥1 daily cigarette) and are referred for or receiving antenatal care. Participants must have sufficient understanding of English to give informed consent; agree to try quitting smoking with NRT within 14 days, receive and send short message/messaging service text messages, and instal the trial’s data collection app on their smartphone.

#### Exclusion criteria

They are ineligible if already using NRT or are enrolled in a smoking cessation study, NHS stop smoking support or a cessation-orientated text message service, or they intend to continue using e-cigarettes or have contraindications to NRT.

#### Recruitment

Participants will be identified from:

1. NHS clinical settings, by poster, direct contact from researchers (face-to-face or distanced) and online, with adverts in NHS digital spaces.
2. Online, outside of NHS settings.

#### NHS settings

These can be hospital antenatal care, general practice, community midwifery or stop-smoking service settings. Researchers may identify potential participants from medical records, contacting them by letter, telephone, email or text before appointments, and including QR code/links to or paper copies of a Patient Information Sheet (PIS) (see online supplemental file). They may also approach pregnant people attending clinics asking them to complete an eligibility screening questionnaire or give them a summary leaflet that contains links/QR codes leading to the PIS. Depending on the setting, researchers may consent to those who are interested and eligible, or they may pass contact details to the trial team (see online supplemental file) to enable consent to be received by them.

10.1136/bmjopen-2024-087175.supp1 Supplementary data

Posters describing the trial will be displayed in clinical areas or appropriate NHS digital spaces; these will include QR codes or links leading to the PIS and to an online version of the screening questionnaire, following which eligible and interested people will be invited to leave contact details in a secure RedCap database hosted by the University of Nottingham. The trial team can then access contact details and contact potential participants directly.

#### Online, outside of NHS settings

Google or social media (eg, Facebook, Instagram,) adverts will be targeted at those who smoke and are pregnant. Embedded links will lead to a study information webpage and an eligibility questionnaire, and those who are potentially eligible will be asked to enter contact details, as above.

Interested, eligible potential participants will be given at least 24 hours to consider the PIS before discussion with a researcher and informed consent is received. Discussion and documentation of consent could be by face-to-face (using ‘wet ink’) or ‘distanced’ using either an online form or by telephone (see online supplemental file). If the online form is used, a link is sent to eligible potential participants and then they will fill out the form during a telephone conversation with a researcher. Consent via telephone is similar, but in this case, the consent form will be generated by the researcher from the research database, following a strict protocol, and then signed copies will be shared with the participant. For all consent methods, a letter will be sent to the participant’s GP informing them of the enrolment.

### Randomisation and blinding

After obtaining informed consent, participants’ baseline data will be collected before randomising them to either study arm with the York Trials Unit’s web-based system. The randomisation schedule will be computer-generated, with pseudo-random code using random permuted blocks of randomly varying sizes and stratified by gestational age (<14 or ≥14 weeks). Immediately afterward, the trial office receives email confirmation of treatment allocation. Participants and those delivering interventions will be aware of treatment allocations, but researchers who collect data will be blinded. To prevent BMN components from being inadvertently delivered to the usual care group, two separate teams of SSPs will be used to deliver smoking cessation support.

### Interventions

#### Control

The usual care for smoking cessation, following the National Centre for Smoking Cessation Training (NCSCT) standard treatment programme,^28^ comprises:

1. Helping set a QD.
2. Conducting up to six telephone or video call counselling sessions.
3. Offering NRT as a patch, short-acting NRT or combined (‘dual NRT’).^9 10^

##### Before QD

SSPs assess participants’ suitability for NRT in terms of cautions or contraindications, other prescription medications and health issues and counsel participants on how best to use NRT as per the NCSCT guidelines. Guidelines advise the ‘not a puff’ rule where NRT should only be used when not smoking. If there is doubt about NRT safety, participants’ GPs are consulted to assess their medical suitability for using NRT products. Participants are mailed a 14-day supply of their chosen NRT product(s) and are instructed to start this on the QD.

##### After the QD

Practitioners offer counselling appointments between the QD and Day 3 and on Days 7, 14, 21 and 28 after the QD. SSPs ask about the withdrawal, use of NRT and experience of nicotine side effects and advice on effective NRT use. On Day 14, participants still using NRT are offered a further 14-day supply.

##### NRT

Advice on the use of dual therapy (one long-acting and one short-acting product) with a dose titrated to the number of cigarettes smoked per day was given. For those who cannot tolerate patches, two short-acting products can be substituted with advice on how to ensure round-the-clock coverage. Participants may choose from the following products supplied in their original packaging; all have UK licenses for use in pregnancy:

##### Patches

Daily Nicorette 16 hours (15 mg or 25 mg) or NiQuitin clear 24 hours (14 mg or 21 mg).

##### Short-acting NRT

Nicorette inhaler 15 mg (max six cartridges/day), Nicorette Cools lozenges 2 mg or 4 mg (max 8–12 lozenges/day), Nicorette QuickMist mouth spray 1 mg/spray (max two sprays at a time; four sprays/hour; 64 sprays/day).

For both trial groups, support beyond 28 days during which trial interventions are delivered is provided by locally available UC NHS support. Participants were encouraged to attend all counselling sessions with reminder texts sent prior to appointments and up to three follow-up scheduled texts if they did not attend.

#### Intervention

The BMN intervention is offered alongside UC (described above) and integrated into an identical schedule. BMN is described in detail elsewhere^26^ and comprises tailored behavioural support designed to encourage adherence to NRT and increase quit rates during pregnancy. The main components are as follows:

##### Counselling

Participants are asked to complete a short questionnaire to assess their concerns and necessity beliefs regarding the use of NRT in pregnancy^29^; the latter are views on how worthwhile NRT might be to participants. The number of counselling sessions mirrors that of UC, but the content addresses individual concerns and beliefs about the safety of nicotine and the efficacy of nicotine replacement products. The first counselling session is on average 10 min longer than those delivered in UC, is delivered via video call where possible and addresses individual concerns and beliefs about the safety of nicotine. To ensure advice is as personalised as possible, SSPs respond to key concerns and necessary beliefs recorded on questionnaires. Participants are advised, if needed, to use a patch and short-acting NRT preparation until childbirth and during brief lapses to smoking (of up to 14 days), provided they still try to quit. To avoid morning cravings, 24-hour NRT patches may be left on overnight, and support is aimed at maintaining adherence to NRT. Follow-up calls are mainly by telephone; these again focus on addressing concerns about nicotine, using sufficient NRT and not stopping this during brief smoking lapses, in addition to the UC advice.

##### Leaflet and website

These reinforce key NRT adherence-enhancing messages using video animations and careful wording. Additionally, there are video clips from experts and/or written experiences with NRT from other pregnant women.

##### Text messages

For up to 30 days, we send personally tailored, automated texts, based on participants’ smoking behaviours and NRT-related beliefs. These aim to support participants’ abstinence; encourage using sufficient NRT to control withdrawal symptoms and cravings; counter intentional non-adherence to NRT (eg, due to nicotine-related concerns) and provide prompts or reminders to prevent non-intentional non-adherence to NRT (eg, forgetting).

#### Staff delivering interventions

All SSPs delivering support are trained to the recognised NCSCT standard required for delivering UC stop smoking support in the UK NHS.^26^ All intervention group participants are counselled by specially trained SSPs working within the research team, who deliver the BMN components integrated into UC. Control group counselling is provided by either a separate group of research team SSPs or by NHS providers responsible for providing locally available UC stop-smoking support.

### Data collection

**Figure 1:**
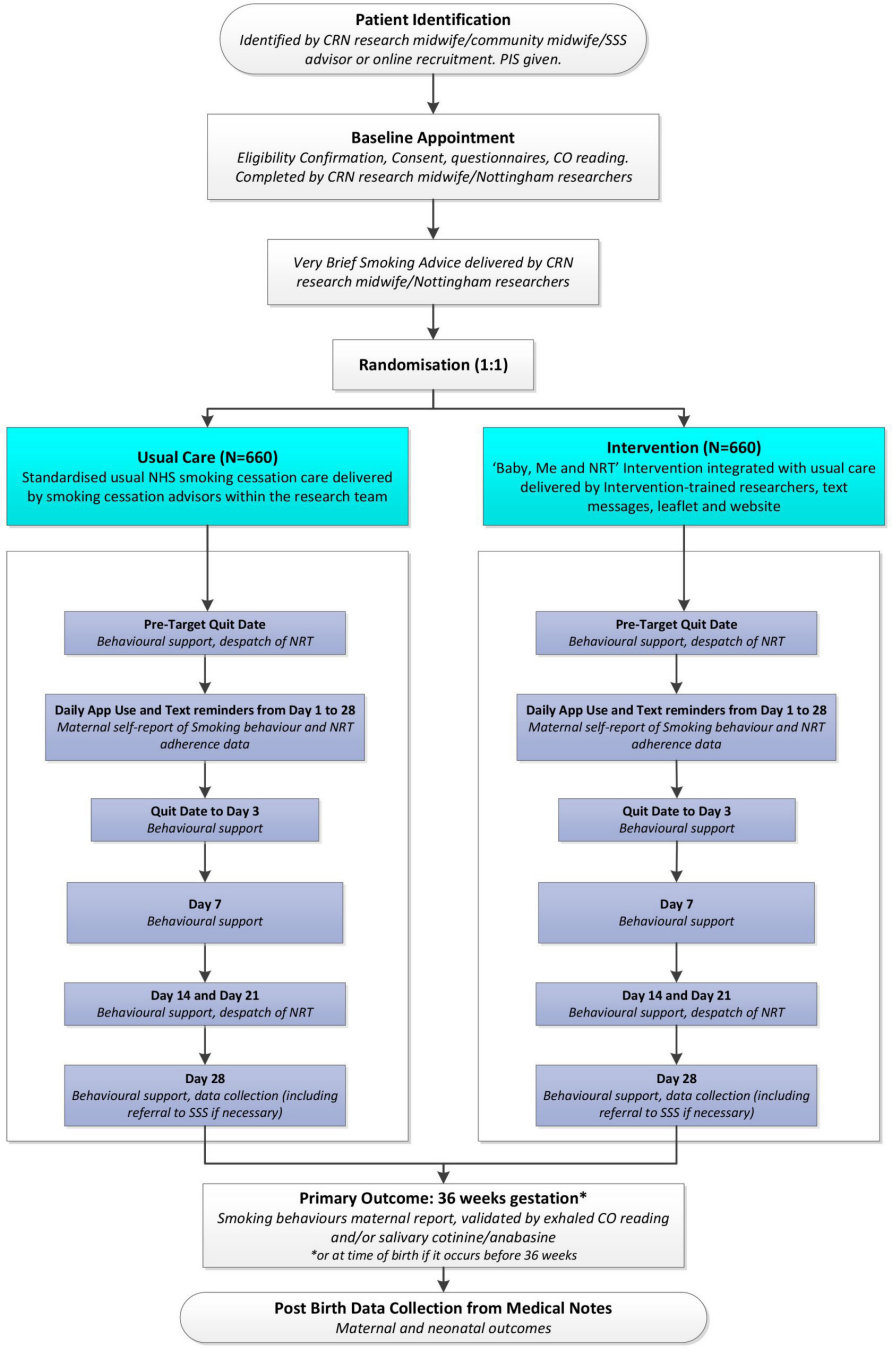
Study flowchart. CO, carbon monoxide; NHS, National Health Service; NRT, Nicotine Replacement Therapy; PIS, Patient Information Sheet.

Table 1 shows all participant data collection at time points outlined below and indicates how intervention delivery fits with this. Figure 1 is a study flow diagram. We indicate which measures will be used for research purposes in the pilot only.

**Table 1:** Schedule of data collection and intervention delivery time points

Data collected	Time point								
	Pre-baseline (consent)	Baseline*	Pre-Quit Date	Days 1–3	Day 7	Day 14	Day 21	Day 28	Delivery (Week 36 gestation)
Informed consent	X†								
Smoking status/ CPD/use of ecigs		X			P‡			X	X
Cravings & tobacco withdrawal		P			P			P	
NRT concerns & necessity beliefs		P						P	
Saliva samples		P			P				X
Exhaled CO		X							X
NRT adherence data					P			X	X
Reported engagement with intervention								X	
NicUse app data collection				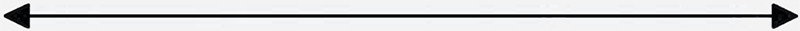					
Medical records data									X
Intervention Delivery (both trial arms)									
Counselling from SSPs offered			X	X	X	X	X	X	
NRT dispatch			X		X	X	X	X	

*Randomisation follows baseline data collection

†X=data collected in both pilot and full trial phase

‡P=data collected only in the pilot phase

#### Baseline

We will ask participants about demographics, gestation, estimated date of delivery, partner smoking status, whether they smoked in previous pregnancies, nicotine dependence,^30^ current and pre-pregnancy smoking behaviours, experience using NRT, smoking beliefs, urges to smoke (cravings) and tobacco withdrawal symptoms.^31^ Where possible, those recruited in person will also provide an expired air CO reading at baseline. Researchers will also help participants to instal the ‘NicUse’ smartphone app from Google Play and Apple Store,^32^ a bespoke app developed for SNAP2 on which applicants record daily smoking, e-cigarette and NRT use (see below) during the 28-day intervention period. We will seek contact details for participants’ GPs and check with them if there is any reason why a participant should not be enrolled.

##### Pilot phase only

All participants were sent an online questionnaire measuring concerns and necessaryu beliefs about NRT.^29^ Those recruited in person were asked (at baseline) for a saliva sample and, if possible, a CO reading given a self-return kit for collection and return of another saliva sample on Day 7 of their quit attempt. Those recruited online or remotely were mailed two self-collection kits with instructions for returning one saliva sample immediately, and another on Day 7 of their quit attempt. Before the sample collection, we asked participants, verbally or via a questionnaire in the postal return kit, when they last smoked and used an e-cigarette and if they were using NRT, which type(s) and when this was last used.

#### Follow-up

After baseline, apart from data collection by app and routine sources, the primary data collection mode is by online questionnaires, with links texted or emailed to participants. If there is no response, a reminder text and/or email will be sent first, then participants will be called and hard copy questionnaires with reply paid envelopes will be posted as a final option.

#### Daily NicUse recording of behaviours

NicUse works on Android or Apple smartphones. Participants will be asked to use the app daily to record smoking behaviour, NRT and/or e-cigarette use. If participants miss reporting for 1 or 2 days, they can record these data retrospectively. Participants receive text messages to prompt completion of the app. The survey on NRT will ask participants to record patch and short-acting NRT use, and the number of units of short-acting NRT consumed, which allow us to calculate their daily nicotine dose.^33^ Compared with questionnaires, NicUse facilitates a more complete collection of NRT adherence data, with more robust face validity, and is less likely to overestimate adherence than questionnaires.^33^

#### Day 7 after the QD (pilot phase only)

We were asked if a quit attempt was made; if any NRT had been used since the QD and, if so, how many days, which types; and if short-acting NRT is used, how many lozenges, cartridges or sprays were used. We also asked about current smoking, e-cigarette use, urges to smoke (cravings) and tobacco withdrawal symptoms. As soon as possible after Day 7, saliva samples (see above) were collected from participants. After the pilot, we discontinued data collection at this time point, as this information was only required for pilot phase outcomes.

#### Day 28 after the QD

We will use the same questions as in the pilot phase-only Day 7 follow-up but will ask about the previous 28 (not 7) days, and we will ask the intervention group about BMN components (eg, texts and website). Participants who do not provide information on smoking status or NRT use by app or questionnaire methods will be sent a text about NRT use since the QD and asked to reply directly.

##### Pilot phase only

Participants were asked to re-complete the NRT concerns and necessity belief measures they first completed at baseline.^29^

#### 36 weeks of gestation or delivery (if earlier)

We will ask participants about smoking, adherence to NRT since 28-day follow-up and use of NHS stop smoking support. We will send non-responders a direct-reply text message asking about smoking in the previous 7 days.

#### Routine data collected from medical records

NHS hospitals collect smoking status and exhaled CO from every woman from week 36 of gestation onwards, and we will collect these data from NHS records. We will also extract maternal and fetal pregnancy outcome data from medical records. In NHS hospitals, research staff will collect these data; otherwise, the research team contact relevant NHS staff to ask for this information. Where birth outcome data cannot be obtained from records, using methods outlined above, we will ask participants to provide birth weight, gestation at delivery, whether they underwent caesarean section or the baby was admitted to special care or had any congenital abnormality and whether they were a smoker or non-smoker at delivery.

#### Validation of smoking abstinence

For participants who report 7-day smoking abstinence at 36 weeks or later, we will collect saliva samples as they attend hospital, at home visits or postally (see above). Before giving samples, participants will be asked about any recent smoking, vaping or NRT use.

#### Financial incentives encouraging data return

To recognise time taken for study participation, participants will receive up to £50 in ‘Love to Shop’ gift cards, which cannot be redeemed for tobacco or alcohol. To receive maximum remuneration, participants will need to submit all adherence app data; they will receive 50p for each daily app report, and an additional £1.50 for supplying a continuous full week’s reports, plus an additional £5 if they report for all 28 days after their QD (maximum total £25). Further gift cards will be given to women if they provide requested questionnaire data and validation saliva samples.

#### Fidelity assessment (pilot phase only)

We will audio-record all initial intervention group consultations, selecting a random sample for further scrutiny. Two researchers will listen to the selected audio recordings, independently rating the completeness of intervention delivery against a fidelity checklist that lists key components of the BMN intervention, and inter-rater reliability between researchers will be determined. We will store recordings on a secure University of Nottingham server for a maximum of 7 years.

#### Data management

Each participant will be assigned a unique study identification number allocated at consent to identify their data and biological samples. Personal identifiers (name, email address and phone number) will be stored in a password-protected computer database accessible only by the researchers. Data will be entered into a REDCap database where possible, but paper case report forms may be used as source data and entered by researchers into the database. Information submitted by participants via the NicUse app is stored as pseudonymised data on the Amazon cloud.

All electronic data will be securely stored at the University of Nottingham for 15 years after which it will be destroyed. Data management will be led by the York Trials Unit, with the support of the Trial Manager at the University of Nottingham as detailed in the Data Management Plan.

The Trial Management Group is responsible for the day-to-day running of the trial, meeting regularly and is supported and reportedby the Trial Steering Committee (TSC) (see online supplemental file).

#### Saliva samples

Saliva samples will be collected by researchers or by participants and sent by post directly to ACM labs for storage and analysis at the end of the trial. The laboratory will quantify salivary cotinine concentrations and the presence or absence anabasine using a quantitative enzyme immunoassay technique. Once the analysis has been completed, the saliva samples will be destroyed in accordance with the Human Tissue Act 2004.

### Outcomes

#### Primary outcome

Reported smoking abstinence in late pregnancy or around childbirth with appropriate biochemical validation.

#### Secondary outcomes

1. Reported smoking abstinence at *both* 28 days and in late pregnancy or at childbirth, with and without appropriate biochemical validation in late pregnancy.
2. Reported smoking abstinence for 24 hours and 7 days at 28 days.
3. Reported number of days NRT is used in the first 28 days following a QD.
4. Reported mean daily nicotine dose in the first 7 days of quitting (‘intensity’ of NRT use).
5. Engagement with BMN intervention components.

#### Pilot phase outcomes

1. Urges to smoke, ‘cravings’, and tobacco withdrawal symptoms.
2. NRT concerns and necessity beliefs at baseline and Day 28.
3. Fidelity of intervention delivery as measured against fidelity checklist.

#### Other outcomes

1. Saliva cotinine concentration.
2. Number of days NRT use between a QD and the end of pregnancy.
3. Exhaled CO concentration.
4. Birth weight.
5. Low birth weight (<2500 g).
6. Gestational age at birth.
7. Maternal or fetal death (stillbirth or miscarriage).
8. Caesarean section delivery.
9. Neonatal intensive care admission.
10. Congenital anomaly.

### Sample size and justification

#### Design changes

Originally, we planned SNAP2 as an RCT to test the extent to which BMN did or did not increase adherence to NRT; adherence to NRT was intended as the primary outcome and smoking outcomes as secondary. If study findings gave a sufficiently positive ‘signal’ for an effect on NRT adherence, we planned a second RCT to test whether BMN had positive effects on smoking cessation. However, due to the COVID-19 pandemic and NHS service provision changes, the funder agreed that BMN efficacy for smoking abstinence would be better investigated instead of simply using a measure of smoking behaviour as the trial primary outcome and increasing the SNAP2 sample size. This was dependent on a successful pilot phase of the trial in which (a) BMN demonstrated a sufficiently large ‘signal’ that impacts the adherence to NRT and (b) explanatory trial outcome data were collected before being discontinued in the more pragmatic ‘full’ trial. Below we detail how, in the SNAP2 pilot phase, the impact of BNM on adherence to NRT was assessed, and progression decided on.

#### Assessment of BMN’s potential effect on NRT adherence’

For the original SNAP2 RCT, we defined a clinically important effect as BMN increasing NRT use by 21%. Data from a previous study indicated that if the control group was offered NRT for 28 days, they were likely to use this on a mean of 7 days,^34^ so a 21% increase represented an extra 1–2 days of NRT use.

We used a one-sided CI approach^35^ to assess whether or not the pilot phase SNAP2 trial ‘signal’ for the impact of BMN on NRT adherence was consistent with assumed effects. Using a CI approach, we calculated that for the checkpoint analysis to produce an upper limit of a one-sided 80% or 90% CI that excludes the estimate of effect, assuming the treatment estimate from the checkpoint assessment was zero or less would require 34 or 54 in the analysis. A trial statistician estimated BMN efficacy using data from 49 participants followed up to the primary outcome point. The point estimate and the upper confidence limit (whether 80% or 90%) were greater than the pre-specified clinically relevant effect size of 1.21. The funder deemed this sufficient demonstration of potential efficacy for the pilot to progress to a full trial.

#### Full trial sample size estimate

To determine the sample size for the full trial, we assume a control group quit rate of 10%, consistent with similar UK studies,^34 36 37^ and seek to detect an absolute increase in the risk of abstinence of 6% (corresponding to a risk ratio (RR) of 1.6). We think this is a reasonable effect size to seek as all previous trials of NRT used by pregnant women tested the use of only one NRT product and these show a relatively weak, imprecise effect (RR 1.37, 95% CI 1.08 to 1.74),^13^ with smaller point estimates seen in the least biased studies (RR 1.21, 95% CI 0.95 to 1.55).^13^ However, in non-pregnant smokers where adherence to NRT is much stronger, depending on the type of NRT used, Cochrane review point estimates for NRT efficacy range between 1.49 (for gum) and 2.02 (inhaler),^38^ and dual NRT (patch+fast acting) is more effective than single product use, with an OR for abstinence with NRT of 1.25 (dual vs single product NRT use).^12^ We aim to recruit 1320 participants, providing a minimum of 90% power for a two-sided test of size 5%, assuming the control group’s quit rate is at least 10%.

### Analyses

Statistical analysis will be conducted when the trial ends using Stata/MP v18 or later unless specified otherwise. All analysis software used including any community-contributed software will be explicitly cited in any publication of the trial results. Significance tests will be two-sided at the 5% level unless specified otherwise. Point estimates will be presented with their associated 95% CIs. Full analyses will be detailed in a statistical analysis plan (SAP), finalised before the end of data collection, and which will be reviewed by the TSC. A CONSORT flow diagram will be provided to display the flow of participants through the study. Baseline data will be summarised descriptively by group, for all randomised and for all those who are included in the primary analysis. No formal statistical comparisons of group differences at baseline will be conducted. Continuous measures will be reported as means and SD, while categorical data will be reported as counts and percentages.

#### Assessment of efficacy

The primary efficacy endpoint is reported abstinence in late pregnancy or around childbirth, validated by appropriate biochemical measures (binary—abstinent or non-abstinent). The primary analysis model will include all randomised participants as part of the groups to which they were allocated, with any missing primary endpoints imputed as being non-abstinent. We will compare abstinence between treatment groups using logistic regression, with fixed effects of the treatment group, gestation at baseline (the stratification factor) and other predictive baseline covariates (pre-specified in the SAP). In addition to the point and interval estimate of the OR for allocation, we will use the fitted model to obtain estimates of treatment effects on both the relative risk and risk difference scales. Binary secondary outcomes (eg, other abstinence outcomes) will be analysed in a broadly similar manner to the analysis of the primary outcome. Secondary outcomes including adherence to NRT, NRT adherence intensity, cotinine (smoking exposure), necessities/concerns and birth outcomes will be compared between arms using appropriate generalised linear models. All other outcomes will be summarised descriptively by a randomised arm. If the relevant data are sufficiently complete, we will perform exploratory analyses to decompose the total effect of allocation on the primary outcome into indirect effects (ie, those mediated by improved NRT adherence/usage) and direct effects (ie, those not mediated by improved NRT adherence/usage).

#### Procedures for missing, unused and spurious data

We will assume that participants with missing smoking status are smoking, in line with the Russell standard, meaning people who do not provide data are assumed to be smoking.^39^ Similarly, participants not providing a NRT usage response(s) will be assumed to be not using NRT in the corresponding period. We will investigate the sensitivity of results from the main analyses to departures from these strict missing not at random (MNAR) assumptions, by imputing these missing data under a range of missing at random (MAR) and MNAR scenarios.

#### Safety

The study tests a behavioural intervention aimed at optimising NRT use.^26^ As this is a standard NHS treatment, we do not anticipate any harm being caused, and there is no adverse event monitoring.

#### Economic analysis

The economic analysis will determine the cost-effectiveness using a lifetime time horizon. To estimate long-term benefits, costs and cost-effectiveness, and potential longer-term cost savings, we will use the Economics of Smoking in Pregnancy (ESIP) model, a bespoke, dynamic economic model designed specifically for valuing smoking cessation in pregnancy in economic terms.^40 41^ Smoking cessation rates from the trial and costs of intervention delivery and usual care will be used as ESIP inputs. As SNAP 2 has a short follow-up period, we will not use measures of participants’ quality of life (QoL) as one would not expect changes in response to the SNAP2 intervention within the short study timeframe, and any QoL changes resulting from smoking cessation would likely be variation in QoL reflecting physiological changes in pregnancy.

### Patient and public involvement

A user and public involvement advisory group has been set up for our N-READY research programme; the current trial is part of this programme. This advisory group consists of women who currently smoke, who have smoked at any point during pregnancy, who are of childbearing age or who are smokers/ex-smokers. This advisory group contributes to all stages of the study, from reviewing study-related documentation and materials to dissemination of research findings. Intervention development, via previous workstreams of the N-READY programme, has already been heavily informed by this group and by interviews with women who smoke (or have smoked) during pregnancy.

## Ethics and dissemination

Ethics approval has been granted by the Bloomsbury NHS Research Ethics Committee (REC reference: 21/LO/0123; Protocol number: 20074. IRAS project ID: 287771). Written informed consent will be obtained from all participants. The findings will be disseminated to the public, funders, relevant practice and policy representatives and other researchers. A data-sharing agreement has been published; once the trial has finished and the main trial paper has been published, a fully anonymised trial data set will be available on reasonable request from York Trials Unit.

### Trial and recruitment status

Recruitment began in June 2021. We completed the recruitment of 264 participants, 100% of the target sample size, to the pilot study in October 2022. The main study aims to recruit 1320 participants by December 2025 with current recruitment on track to achieve this, and follow-up to continue for another 10 months with the participant last visit due in October 2026.

Due to the pandemic, recruitment via traditional routes became problematic. In response to these difficulties, we have successfully recruited via paid social media advertising. Of the first 265 participants, 185 (69.8%) were recruited via hospital antenatal settings and 80 (30.2%) via online recruitment routes.

10.1136/bmjopen-2024-087175.supp2 Supplementary data

10.1136/bmjopen-2024-087175.supp3 Supplementary data

## Supplementary Material

Reviewer comments

Author's manuscript

## References

[1] NHS Digital . Statistics on women’s smoking status at time of delivery: England. Quarter 2024;2:2023–4. Available: https://digital.nhs.uk/data-and-information/publications/statistical/statistics-on-women-s-smoking-status-at-time-of-delivery-england/statistics-on-womens-smoking-status-at-time-of-delivery-england-quarter-2-2023-24

[2] Chamberlain C , O’Mara-Eves A , Oliver S , et al . Psychosocial interventions for supporting women to stop smoking in pregnancy. Cochrane Database Syst Rev 2013;10:CD001055. 10.1002/14651858.CD001055.pub4 24154953 PMC4022453

[3] Cnattingius S . The epidemiology of smoking during pregnancy: smoking prevalence, maternal characteristics, and pregnancy outcomes. Nicotine Tob Res 2004;6:S125–40. 10.1080/14622200410001669187 15203816

[4] Leonardi-Bee J , Jere ML , Britton J . Exposure to parental and Sibling smoking and the risk of smoking uptake in childhood and adolescence: a systematic review and meta-analysis. Thorax 2011;66:847–55. 10.1136/thx.2010.153379 21325144

[5] World Health Organization . Report on the Global Tobacco Epidemic 2008—the Mpower Package. Geneva: World Health Organization, 2008.

[6] Cooper S , Orton S , Leonardi-Bee J , et al . Smoking and quit attempts during pregnancy and postpartum: a longitudinal UK cohort. BMJ Open 2017;7:e018746. 10.1136/bmjopen-2017-018746 PMC5695489 29146659

[7] National Institute for Health and Clinical Excellence . Quitting smoking in pregnancy and following childbirth. NICE public health guidance 26. London; report no.: Ph26. 2010.

[8] National Institute for Clinical Excellence . Tobacco: preventing uptake, promoting quitting and treating dependence. NICE guideline [Ng209]. 2021. Available: https://www.nice.org.uk/guidance/ng209/chapter/Recommendations-on-treating-tobacco-dependence-in-pregnant-women#providing-support-to-stop-smoking 38598649

[9] Fahy SJ , Cooper S , Coleman T , et al . Provision of smoking cessation support for pregnant women in England: results from an online survey of NHS stop smoking services for pregnant women. BMC Health Serv Res 2014;14:107. 10.1186/1472-6963-14-107 24593130 PMC3975862

[10] Cooper S , Orton S , Campbell KA , et al . Attitudes to E-cigarettes and cessation support for pregnant women from English stop smoking services: A mixed methods study. Int J Environ Res Public Health 2019;16:110. 10.3390/ijerph16010110 30609823 PMC6338976

[11] Dhalwani NN , Szatkowski L , Coleman T , et al . Prescribing of nicotine replacement therapy in and around pregnancy: a population-based study using primary care data. Br J Gen Pract 2014;64:e554–60. 10.3399/bjgp14X681361 25179069 PMC4141612

[12] Lindson N , Chepkin SC , Ye W , et al . Different doses, durations and modes of delivery of nicotine replacement therapy for smoking cessation. Cochrane Database Syst Rev 2019;4:CD013308. 10.1002/14651858.CD013308 30997928 PMC6470854

[13] Claire R , Chamberlain C , Davey M-A , et al . Pharmacological interventions for promoting smoking cessation during pregnancy. Cochrane Database Syst Rev 2020;3:CD010078. 10.1002/14651858.CD010078.pub3 32129504 PMC7059898

[14] Hollands GJ , Sutton S , McDermott MS , et al . Adherence to and consumption of nicotine replacement therapy and the relationship with abstinence within a smoking cessation trial in primary care. Nicotine Tob Res 2013;15:1537–44. 10.1093/ntr/ntt010 23430709 PMC3741059

[15] Bowker K , Lewis S , Coleman T , et al . Changes in the rate of nicotine metabolism across pregnancy: a longitudinal study. Addiction 2015;110:1827–32. 10.1111/add.13029 26119134 PMC5014174

[16] Dempsey D , Jacob P , Benowitz NL . Accelerated metabolism of nicotine and Cotinine in pregnant Smokers. J Pharmacol Exp Ther 2002;301:594–8. 10.1124/jpet.301.2.594 11961061

[17] Bowker K , Campbell KA , Coleman T , et al . Understanding pregnant Smokers’ adherence to nicotine replacement therapy during a quit attempt: a qualitative study. Nicotine Tob Res 2016;18:906–12. 10.1093/ntr/ntv205 26391578 PMC5942617

[18] Campbell K , Coleman-Haynes T , Bowker K , et al . Factors influencing the uptake and use of nicotine replacement therapy and E-cigarettes in pregnant women who smoke: a qualitative evidence synthesis. Cochrane Database Syst Rev 2020;5:CD013629. 10.1002/14651858.CD013629 32441810 PMC7387757

[19] Thomson R , McDaid L , Emery J , et al . Knowledge and education as barriers and Facilitators to nicotine replacement therapy use for smoking cessation in pregnancy: A qualitative study with health care professionals. Int J Environ Res Public Health 2019;16:1814. 10.3390/ijerph16101814 31121850 PMC6571581

[20] Fergie L , Campbell KA , Coleman-Haynes T , et al . Stop smoking practitioner consensus on barriers and Facilitators to smoking cessation in pregnancy and how to address these: A modified Delphi survey. Addict Behav Rep 2019;9:100164. 10.1016/j.abrep.2019.100164 31193880 PMC6543497

[21] Fergie L , Coleman T , Ussher M , et al . Pregnant Smokers’ experiences and opinions of techniques aimed to address barriers and Facilitators to smoking cessation: A qualitative study. Int J Environ Res Public Health 2019;16:2772. 10.3390/ijerph16152772 31382531 PMC6695602

[22] Benowitz NL . The use of Pharmacotherapies for smoking cessation during pregnancy. Tob Control 2000;9:91iii–94. 10.1136/tc.9.suppl_3.iii91 PMC1766294 10982920

[23] Taylor L , Claire R , Campbell K , et al . Fetal safety of nicotine replacement therapy in pregnancy: systematic review and meta-analysis. Addiction 2021;116:239–77. 10.1111/add.15185 32621526

[24] Coleman T , Cooper S , Thornton JG , et al . A randomized trial of nicotine-replacement therapy patches in pregnancy. N Engl J Med 2012;366:808–18. 10.1056/NEJMoa1109582 22375972

[25] Cooper S , Taggar J , Lewis S , et al . Effect of nicotine patches in pregnancy on infant and maternal outcomes at 2 years: follow-up from the randomised, double-blind, placebo-controlled SNAP trial. Lancet Respir Med 2014;2:728–37. 10.1016/S2213-2600(14)70157-2 25127405

[26] McDaid L , Emery J , Thomson R , et al . A behavioral intervention to improve the effectiveness of nicotine replacement therapy in pregnancy. Nicotine Tob Res 2023;25:1770–80. 10.1093/ntr/ntad102 37349134 PMC10475605

[27] Chan AW , Tetzlaff Jm Fau - Gøtzsche PC , Gøtzsche Pc Fau - Altman DG , et al . n.d. SPIRIT 2013 explanation and elaboration: guidance for protocols of clinical trials. (1756-1833 (electronic)). 10.1136/bmj.e7586 PMC3541470 23303884

[28] Papadakis S , Hermon Y , McEwan A . Standard Treatment Programme for Pregnant Women: A guide to behavioural support for smoking cessation during pregnancy and the post-partum period: National Centre for Smoking Cessation and Training (NCSCT), 2019. Available: https://www.ncsct.co.uk/usr/pub/NCSCT%20Standard%20Treatment%20Programme%20for%20Pregnant%20Women.pdf

[29] Emery J , McDaid L , Coleman T , et al . Development and content validation of a questionnaire for measuring beliefs about using nicotine replacement therapy for smoking cessation in pregnancy. Nicotine Tob Res 2023;25:1310–8. 10.1093/ntr/ntad030 36861351 PMC10256886

[30] Heatherton TF , Kozlowski LT , Frecker RC , et al . Measuring the heaviness of smoking: using self-reported time to the first cigarette of the day and number of cigarettes smoked per day. Br J Addict 1989;84:791–9. 10.1111/j.1360-0443.1989.tb03059.x 2758152

[31] West R , Hajek P . Evaluation of the mood and physical symptoms scale (MPSS) to assess cigarette withdrawal. Psychopharmacology (Berl) 2004;177:195–9. 10.1007/s00213-004-1923-6 15179542

[32] Huang Y , Emery J , Naughton F , et al . The development and acceptability testing of an App-based smart survey system to record smoking behaviour, use of nicotine replacement therapy (NRT) and E-cigarettes. BMC Res Notes 2022;15:100. 10.1186/s13104-022-05983-8 35272684 PMC8908557

[33] Emery J , Huang Y , Naughton F , et al . Comparison of a daily Smartphone App and retrospective questionnaire measures of adherence to nicotine replacement therapy among pregnant women: observational study. JMIR Form Res 2023;7:e35045. 10.2196/35045 36881452 PMC10031440

[34] Coleman T , Cooper S , Thornton JG , et al . A randomized trial of nicotine-replacement therapy patches in pregnancy 8. N Engl J Med 2012;366:808–18. 10.1056/NEJMoa1109582 22375972

[35] Cocks K , Torgerson DJ . Sample size calculations for pilot randomized trials: a confidence interval approach. J Clin Epidemiol 2013;66:197–201. 10.1016/j.jclinepi.2012.09.002 23195919

[36] Ussher M , Lewis S , Aveyard P , et al . Physical activity for smoking cessation in pregnancy: randomised controlled trial. BMJ 2015;350:h2145. 10.1136/bmj.h2145 25976288 PMC4431606

[37] Tappin D , Bauld L , Purves D , et al . Financial incentives for smoking cessation in pregnancy: randomised controlled trial. BMJ 2015;350:h134. 10.1136/bmj.h134 25627664

[38] Hartmann-Boyce J , Chepkin SC , Ye W , et al . Nicotine replacement therapy versus control for smoking cessation. Cochrane Database Syst Rev 2018;5:CD000146. 10.1002/14651858.CD000146.pub5 29852054 PMC6353172

[39] West R , Hajek P , Stead L , et al . Outcome criteria in smoking cessation trials: proposal for a common standard. Addiction 2005;100:299–303. 10.1111/j.1360-0443.2004.00995.x 15733243

[40] Jones M , Smith M , Lewis S , et al . A dynamic, Modifiable model for estimating cost-effectiveness of smoking cessation interventions in pregnancy: application to an RCT of self-help delivered by text message. Addiction 2019;114:353–65. 10.1111/add.14476 30347119 PMC6519118

[41] Coleman T , Clark M , Welch C , et al . Effectiveness of offering tailored text message, self-help smoking cessation support to pregnant women who want information on stopping smoking: Miquit3 randomised controlled trial and meta-analysis. Addiction 2022;117:1079–94. 10.1111/add.15715 34636086

