# Supplementary material for: Smoking, nicotine and pregnancy 2 (SNAP2) trial: protocol for a randomised controlled trial of an intervention to improve adherence to nicotine replacement therapy during pregnancy
Source: BMJ Open. 2024 May 28;14(5):e087175. doi: 10.1136/bmjopen-2024-087175 (PMC11138292; doi:10.1136/bmjopen-2024-087175)
Supplement: Supplementary data [file bmjopen-2024-087175supp001.pdf]

Roles and responsibilities

These membership lists are correct at the time of writing:

Roles of trial sponsor and funders

Name	Affiliation	Role
Alison Thorpe	University of Nottingham ('Sponsor')	Representative of the sponsor
Thomas Hutchinson	NIHR ('Funder')	Representative of the funder

Trial Team

Name	Affiliation	Role
Tim Coleman	University of Nottingham	Chief Investigator
Sue Cooper	University of Nottingham	Programme lead for Workstream 3.
Miranda Clark	University of Nottingham	Senior Trial Manager, trial management oversight
Kate Bowker	University of Nottingham	Trial Manager, day to day running of the trial
Lucy Phillips	University of Nottingham	Trial Manager, day to day running of the trial
Karen Daykin/ Nicki Stockdale	University of Nottingham	Trial Manager/Trial Coordinator, day to day running of the trial
Anne Dickinson	University of Nottingham	Researcher, trial intervention delivery and management of the delivery team
Daniel Robertson	University of Nottingham	Trial Coordinator, day to day running of the trial and participant follow up
Kasia Kowalewska	University of Nottingham	Trial Coordinator, day to day running of the trial and participant follow up
Amy Morton	University of Nottingham	Trial administration, participant follow up
Eleanor Holmes	University of Nottingham	Trial administration, participant follow up
Katie Zhoya	University of Nottingham	Trial administration, participant follow up
Michelle Rawding	University of Nottingham	Trial administration, participant follow up
Daniel Simpkins		Senior Data Manager responsible for management of the database
Sarah Gardner	University of York	Database design and build
Matthew Bailey	University of York	Trial database set up and randomisation system build
Ross Thompson	University of Nottingham	Researcher, participant recruitment/consent
Lisa McDaid	University of East Anglia	Researcher, development of the intervention, participant recruitment/consent
Jo Emery	University of East Anglia	Researcher, development of the intervention, participant recruitment/consent
Felix Naughton	University of East Anglia	Programme lead for Workstream 1 and 2 of the NREADY programme

Trial Management Group

Name	Affiliation	Role
Tim Coleman	University of Nottingham	Chief Investigator
Sue Cooper	University of Nottingham	Programme manager
Miranda Clark	University of Nottingham	Senior Trial Manager
Kate Bowker	University of Nottingham	Trial Manager, day to day running of the trial
Lucy Phillips	University of Nottingham	Trial Manager, day to day running of the trial
Karen Daykin/ Nicki Stockdale	University of Nottingham	Trial Manager/Trial Coordinator, day to day running of the trial
Anne Dickinson	University of Nottingham	Researcher, trial intervention delivery and management of the delivery team
Ross Thomson	University of Nottingham	Researcher, participant recruitment/consent

Catherine Hewitt	University of York	Lead Trial statistician
Charlie Welch	University of York	Trial statistician
Gill Parkinson	University of York	Trial statistician
David Torgeson	University of York	Director of the York Trials Unit
Michael Ussher	St Georges, University of London	Population Health Science, and Social Marketing and Health
Sarah Lewis	University of Nottingham	Independent statistician

Trial Steering Committee

**Name**	**Affiliation**	**Role**
Peter Hajek	Queen Mary University London	Independent Chair
Martyn Willmore	Public Health England	Independent member
Jo Locker	Public Health England	Independent member
Donna Wilkes	PPI	Independent PPI Representative
Nikki Totton	University of Sheffield	Medical Independent statistician
Alison Thorpe	University of Nottingham (‘Sponsor’)	Observer (sponsor)
Thomas Hutchinson	NIHR	Observer (funder)
Catherine Hewitt	Lead Trial statistician (York Trials Unit)	Observer
Charlie Welch	Trial statistician (York Trials Unit)	Observer
Gill Parkinson	Trial statistician (York Trials Unit)	Observer
Tim Coleman	University of Nottingham	Non-independent member
Sue Cooper	University of Nottingham	Observer
Miranda Clark	University of Nottingham	Observer
Trial Manager	University of Nottingham	Observer
Felix Naughton	University of East Anglia	Observer
