# Supplementary material for: Smoking, nicotine and pregnancy 2 (SNAP2) trial: protocol for a randomised controlled trial of an intervention to improve adherence to nicotine replacement therapy during pregnancy
Source: BMJ Open. 2024 May 28;14(5):e087175. doi: 10.1136/bmjopen-2024-087175 (PMC11138292; doi:10.1136/bmjopen-2024-087175)
Supplement: Supplementary data [file bmjopen-2024-087175supp002.pdf]

Participant Information Sheet
(Version 2.1 Date 12.09.22)

IRAS Project ID: 287771

Title of Study: **Smoking, Nicotine and Pregnancy 2 Trial (SNAP 2)**

Name of Chief Investigator: Tim Coleman

We would like to invite you to take part in our research study. Before you decide we would like you to understand why the research is being done and what it would involve for you. Please read the information below carefully. One of our team will go through the information sheet with you before you decide to take part and answer any questions you have. Talk to family, friends, or others about the study if you wish. Please ask us if there is anything that is not clear.

What is the purpose of the study?

- We want to improve the support that pregnant women receive to help them stop smoking.
- Pregnant women can use Nicotine Replacement Therapy (NRT) to help them stop smoking and the NHS prescribes this to them for free.
- However, pregnant women often do not use NRT in the best possible way and this can make it less effective than it could be.
- Therefore, in this study, we are testing a package of support which we hope will help pregnant women make better use of NRT so, it will have a better chance of helping them to stop smoking.

Why have I been invited?
We are inviting you to take part because you have told us that you are less than 25 weeks pregnant, smoke and are interested in getting help to quit, like NRT.

Do I have to take part?
It is up to you to decide whether or not to take part. If you do take part you will be given this information sheet to keep and will be asked to complete a consent form on paper, online or by telephone. If you join the study, you will be free to withdraw at any time without giving a reason. This would not affect your legal rights.

What will happen to me if I take part?
A computer will randomly place you into one of two groups with an equal chance of being in either.

Group One will receive support to stop smoking, which is the same as usual NHS support. You will be offered NRT as a patch, short acting NRT such as lozenges, inhalator or mouth spray or both together (‘dual NRT’). You will receive up to six support sessions with a stop smoking practitioner (SSP). The first session will take place just before the day on which you stop smoking (quit date) and will last approximately *30 minutes*. Further consultations will be offered on or around *Day 3, Day 7, Day 14, Day 21, and Day 28* after your quit date. These will take place by telephone or video call and will last approximately *15 minutes* each.

Group Two will receive the same usual NHS stop smoking support that women in Group One receive, plus an intervention to help them make better use of NRT. This includes special support from a stop smoking practitioner, a leaflet, text messages, and a website. The first session will be just before the quit date and will last no longer than *45 minutes* and follow up consultations will last approximately *15 minutes* each and take place by telephone or video call.

Clark MM, *et al. BMJ Open* 2024; 14:e087175. doi: 10.1136/bmjopen-2024-087175

Having two groups is a very important because it allows us to compare them and to learn about any benefits or disadvantages of the support we are testing. Joining the study will not affect your usual care and, should you decide not to participate, you will be offered the usual NHS support for stopping smoking which is available to you locally.

What would we expect from everyone taking part?

We will contact you by telephone, videocall, email, text or post. For some of the research information, we will send you a link by email or text asking you to complete a short questionnaire online:

- **When you first join the study.**

  We will ask questions about smoking and NRT. This should take no longer than *10 minutes*.

  We may ask you to provide a breath sample to measure your smoking. If you are selected to provide a breath test, we may send you a carbon monoxide meter and we will help you to set up an app on your mobile phone which helps you to record a carbon monoxide reading by blowing into this . Providing breath samples should take no longer than *5 minutes*.

  We will help you to download the NicUse app to your mobile phone. For each of the 28 days after your quit date, we will ask you to tell the app about your NRT use and any smoking and / or e-cigarette use. If you do not answer app questions for 2 days in a row, we will send a text message reminder.

- **Day 28 after your quit date**. We will ask about smoking and NRT and how you have got on providing a breath sample and using the app. If you are in Group 2, we will ask you some additional questions about your experiences of using the website, leaflet and receiving text messages; this should take no longer than *10 minutes*.

- **Towards the end of your pregnancy.** At around 36 weeks, we will ask you some questions about your smoking and NRT use. We may also ask you to provide saliva and breath samples. This should take no longer than *10 minutes*.

We will liaise with the hospital you are booked to deliver your baby to check how your pregnancy is progressing at approximately 34 weeks into your pregnancy, and again at a later date to find out details about the birth of your baby and your smoking status around delivery if required. We may contact you to ask you for information about the birth of your baby and your smoking status around delivery.

We will ask your permission to audio record some of your consultations with the stop smoking practitioner; this will help us monitor the quality of the support that we provide. This is optional. You will be able to take part in the study without agreeing to being recorded but, it would be helpful to the study if you were to agree. Audio recordings of consultations will be kept confidential. Only members of the research team will have access to these.

Below is a summary of what we would expect from you if you decided to take part in this study.

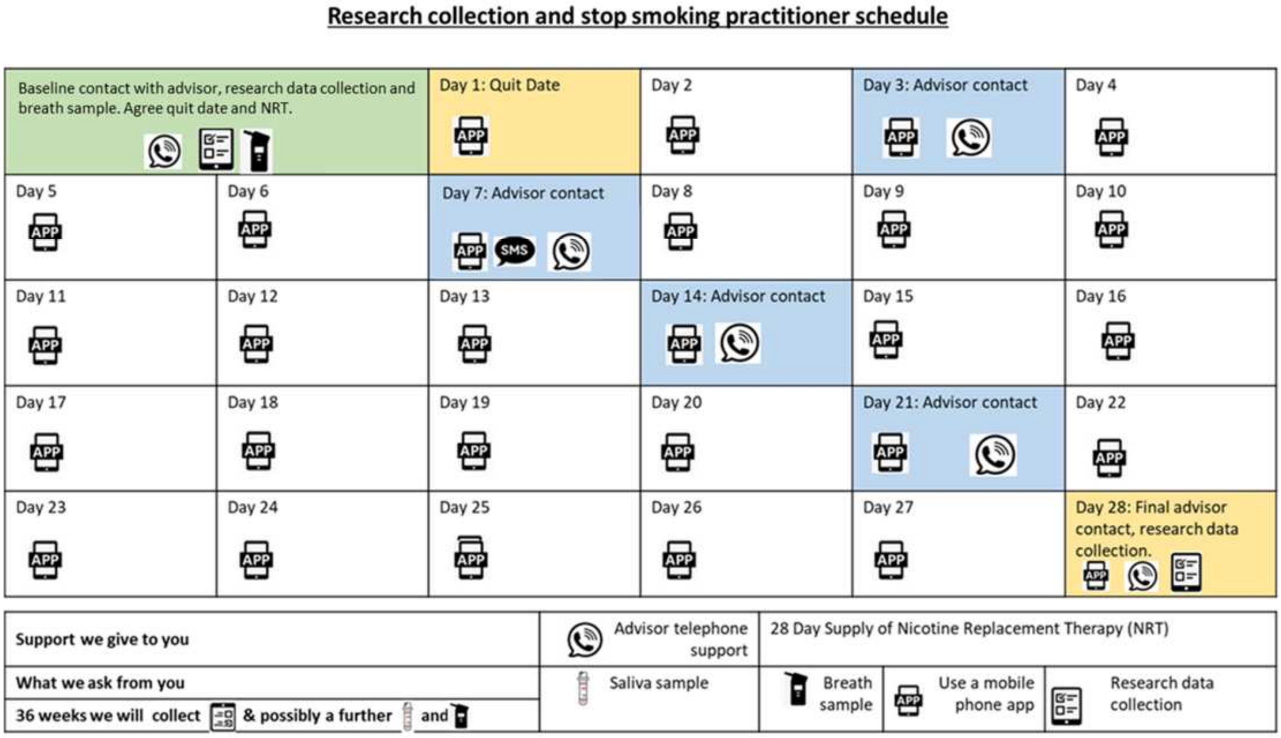

Research collection and stop smoking practitioner schedule

Baseline contact with advisor, research data collection and breath sample. Agree quit date and NRT.		Day 1: Quit Date	Day 2	Day 3: Advisor contact	Day 4
Day 5	Day 6	Day 7: Advisor contact	Day 8	Day 9	Day 10
Day 11	Day 12	Day 13	Day 14: Advisor contact	Day 15	Day 16
Day 17	Day 18	Day 19	Day 20	Day 21: Advisor contact	Day 22
Day 23	Day 24	Day 25	Day 26	Day 27	Day 28: Final advisor contact, research data collection.

Support we give to you	Advisor telephone support	28 Day Supply of Nicotine Replacement Therapy (NRT)		
What we ask from you	Saliva sample	Breath sample	Use a mobile phone app	Research data collection
36 weeks we will collect & possibly a further and				

Expenses

All texts we send you are free, but texts you send to us will be the same as texting from a UK mobile number. Please check with your mobile phone provider about text messaging charges.

What are the possible disadvantages and risks of taking part?

We do not foresee there being any risks from taking part in this study. However, we appreciate that taking part will use your time and may therefore be inconvenient. Also, if you are likely to be upset by receiving some basic information about the risks of smoking in pregnancy then it is best not to take part.

What are the possible benefits of taking part?

We cannot promise the study will help you, but all participants will receive support to stop smoking based on the best NHS standards of practice. The information you provide to us during the study will be invaluable in helping us devise ways of supporting women like you who want to stop smoking during their pregnancy.

What happens when the research study stops?

Once your involvement in the study ends you will continue to receive routine stop smoking support available to NHS patients in the locality, unless you choose not to. We can assist you with this. If you are interested in reading the findings from this study, you can agree for us to keep your contact details after the end of the study, so that we can share the overall results with you once these are available.

What if there is a problem?

If you have a concern about any aspect of the study, you should ask to speak with the study team in Nottingham who will do their best to answer your questions. If you remain unhappy and wish to complain formally, you can do this through the NHS Complaints Procedure. For advice on making a complaint, contact your local Patient Advice and Liaison Service (PALS) at your local hospital.

PALS offers confidential advice, support and information on health-related matters and can provide patients with more information about the complaints procedure and the Independent Complaints Advocacy Service (ICAS).

Will my taking part in the study be kept confidential?

We will follow ethical and legal practice and all information about you will be handled in confidence.
If you join the study, we will use information collected from you and your medical notes during the course of the research. All data will be kept **strictly confidential**, stored in a secure and locked office, and on a password protected database at the University of Nottingham or with trial colleagues at the University of Cambridge. Research data shared with individuals from other Universities who are working within our research team will not have access to identifiable data. Any information shared will use a unique personalised participant study number. Under UK Data Protection laws the University is the Data Controller (legally responsible for the data security) and the Chief Investigator of this study (named at the start of this document) is the Data Custodian (manages access to the data). The University of Nottingham is the data controller for the study. This means we are responsible for looking after your information and using it properly. Your rights to access, change or move your information are limited, as we need to manage your information in specific ways to comply with certain laws and for the research to be reliable and accurate. To safeguard your rights we will use the minimum personally-identifiable information possible.

You can find out more about how we use your information and to read our privacy notice at: https://www.nottingham.ac.uk/utilities/privacy.aspx.

The data collected for the study will be looked at and stored by authorised persons from the research team. They may also be looked at by authorised people from regulatory organisations to check that the study is being carried out correctly. All will have a duty of confidentiality to you as a research participant and we will do our best to meet this duty. Audio recordings will be anonymised and will be accessed by members of the research team. Only members of the research team will have access to any audio recordings where you could be identified. Anonymised transcripts and personal details will be stored separately on a secure network.

If you consent, your contact information will be kept by the University of Nottingham for up to 3 years after the end of the study so that we are able to contact you about the findings of the study and possible follow-up studies. This information will be kept separately from the research data collected and only those who need to will have access to it. All research data will be kept securely for 7 years or longer if required. After this time your data will be disposed of securely. During this time all precautions will be taken by all those involved to maintain your confidentiality, only members of the research team given permission by the data custodian will have access to your personal data.

In order for you to receive the text messaging service, your mobile phone number will be shared with a text carrier called FastSMS and/or Esendex, after the study is completed your confidential information will permanently deleted from these carriers. Their full information security statement can be found here: https://fastsms.co.uk/downloads/fastsms-privacy-policy.pdf and https://www.esendex.co.uk/knowledge-hub/faqs/.

In accordance with the University of Nottingham's, the Government's and our funders' policies we may share our research data with researchers in other Universities and organisations, including those in other countries, for research in health and social care. Sharing research data is important to allow peer scrutiny, re-use (and

therefore avoiding duplication of research) and to understand the bigger picture in particular areas of research. Data sharing in this way is usually anonymised (so that you could not be identified) but if we need to share identifiable information we will seek your consent for this and ensure it is secure. You will be made aware then if the data is to be shared with countries whose data protection laws differ to those of the UK and how we will protect your confidentiality.

Although what you say in the consultations with your stop smoking practitioner is confidential, should you disclose anything to us which we feel puts you or anyone else at any risk, we may feel it necessary to report this to the appropriate persons.

What will happen if I don't want to carry on with the study?

Your participation is voluntary, and you are free to withdraw at any time, without giving any reason, and without your legal rights being affected. If you want to withdraw from the study, you can do so at any time by texting 07537404542, calling us on 0115 7486681, or by emailing snap2study@nottingham.ac.uk

If you withdraw from the study, we will no longer collect any information about you or from you but we will keep the information about you that we have already obtained. This information may have already been used in some analyses and may still be used in the final study analyses. To safeguard your rights, we will use the minimum personally identifiable information possible.

Involvement of the General Practitioner/Family doctor (GP) and hospital

We tell your GP and the hospital where you plan to deliver your baby, that you are taking part in the study and what this involves (with your consent). We will ask your GP if there is any reason why you might not be suitable to take part in this study.

What will happen to any samples I give?

Only members of the research team, relevant regulatory authorities and the University-approved laboratory who test the saliva will have access to the results of your samples. The saliva samples will be tested for the amount of cotinine and / or anabasine in them. Cotinine is a chemical that is produced when nicotine (from cigarettes) is broken down by the body, present in both NRT and tobacco smoke and anabasine is present in tobacco smoke. All samples will be stored in a monitored freezer at the University-approved laboratory that will carry out their testing.

If the saliva sample you provide us is taken by a researcher then they will post this sample to a University-approved laboratory to be tested on the day it was taken from you. We may ask you to post your samples directly to the University-approved laboratory, if samples are taken by yourself at home (using a pre-paid, stamped addressed envelope we will provide you). The sample will have a study number, initials, whether it is your first or subsequent sample, and date of sample, for identification so only the research team will be able to link your sample to you. Once the laboratory has analysed your sample and we have checked the results, the sample will be destroyed in accordance with the Human Tissue Act 2004.

What will happen to the results of the research study?

The results of the study may be presented to other researchers, at conferences and through publication in scientific and medical journals. No names will be used in the results and individuals will not be identifiable in any written reports or presentations. It is also intended that the findings will be used to design new techniques that stop smoking practitioners can use to support women to stop smoking during their pregnancy.

Who is organising and funding the research?

This research is being organised by the University of Nottingham and is being funded the National Institute for Health Research (NIHR), Programmes for Applied Health Research.

Clark MM, *et al. BMJ Open* 2024; 14:e087175. doi: 10.1136/bmjopen-2024-087175

Who has reviewed the study?

All research in the NHS is looked at by an independent group of people, called a Research Ethics Committee, to protect your interests. This study has been reviewed and given favourable opinion by London Bloomsbury Research Ethics Committee.

Further information and contact details

**Chief investigator:**	**Professor Tim Coleman**<br>University of Nottingham<br>Nottingham<br>NG7 2RD<br>Phone: 0115 8230204<br>Email: tim.coleman@nottingham.ac.uk
**Senior Trial Manager**:	**Miranda Clark**<br>University of Nottingham<br>Nottingham<br>NG7 2RD<br>**Email**: miranda.clark@nottingham.ac.uk<br>**Telephone**: 0115 7486681
**General trial:**	**Email:** snap2study@nottingham.ac.uk<br>**Telephone**: 0115 7486681

Clark MM, *et al. BMJ Open* 2024; 14:e087175. doi: 10.1136/bmjopen-2024-087175
