# Supplementary material for: Smoking, nicotine and pregnancy 2 (SNAP2) trial: protocol for a randomised controlled trial of an intervention to improve adherence to nicotine replacement therapy during pregnancy
Source: BMJ Open. 2024 May 28;14(5):e087175. doi: 10.1136/bmjopen-2024-087175 (PMC11138292; doi:10.1136/bmjopen-2024-087175)
Supplement: Supplementary data [file bmjopen-2024-087175supp003.pdf]

# CONSENT FORM

**(Final Version 2.1 12.09.22)**

Title of Study: Smoking, Nicotine and Pregnancy 2 Trial (SNAP 2)

Chief Investigator: Professor Tim Coleman
Principal Investigator:
Participant Name:
Participant Number:

Site Number:
REC ID: 21/LO/0123
IRAS ID: 287771

**Please initial box**

1. I confirm that I have read and understand the Participant Information Leaflet version number 2.1 12.09.22 for the above study and have had the opportunity to ask questions. ☐
2. I understand that my participation is voluntary and that I am free to withdraw at any time, without giving any reason, and without my medical care or legal rights being affected. I understand that should I withdraw then the information collected so far cannot be erased and that this information may still be used in the project analysis. ☐
3. I understand that relevant sections of my medical notes and data collected in the study may be looked at by authorised individuals from the University of Nottingham, the research group (University of East Anglia and University of York) and regulatory authorities where it is relevant to my taking part in this study. I give permission for these individuals, where it is relevant, to collect, store, analyse and publish information obtained from my participation in this study. I understand that my personal details will be kept confidential. ☐
4. I understand and agree that breath samples and/or saliva samples will be requested to measure my carbon monoxide and nicotine levels. ☐
5. I agree to being followed-up by the research team during the study by telephone/video call, text, email, post, or face-to-face appointments. ☐
6. I agree to my GP and hospital where I will deliver my baby being informed of my participation in this study, and to my GP being asked to provide information if there are any reasons that I should not take part. ☐
7. I agree to my mobile phone number being used in this study and consent to it being passed to the University of Cambridge and their nominated text carrier (FastSMS), and/or a different text carrier (Esendex), so that I can receive study related text messages. I understand that my mobile number will only be used for this study. ☐
8. I understand that my anonymised data collected in the study may be used to support other research in the future and may be shared with other researchers. ☐
9. **(Not essential to study participation)** I agree to have my contact details kept after the end of the study (for a maximum of 3 years) so that I can be contacted about the findings of the study and informed of follow-up studies. **Yes** ☐ **No** ☐
10. **(Not essential to study participation)** I agree that the stop smoking consultation I receive can be audio recorded and that anonymous quotes from the consultation may be used in study reports. **Yes** ☐ **No** ☐
11. I agree to take part in the above study. ☐

| Name of Participant | Date | Signature |
|---|---|---|
| Name of Person Taking Consent | Date | Signature |

3 copies: 1 for participant, 1 for the project notes and 1 for the medical notes



Clark MM, *et al. BMJ Open* 2024; 14:e087175. doi: 10.1136/bmjopen-2024-087175
