# Supplementary material for: Smoking, nicotine and pregnancy 2 (SNAP2) trial: protocol for a randomised controlled trial of an intervention to improve adherence to nicotine replacement therapy during pregnancy
Source: BMJ Open. 2024 May 28;14(5):e087175. doi: 10.1136/bmjopen-2024-087175 (PMC11138292; doi:10.1136/bmjopen-2024-087175)
Supplement: Author's manuscript [file bmjopen-2024-087175.draft_revisions.pdf]

BMJ Open is committed to open peer review. As part of this commitment we make the peer review history of every article we publish publicly available.

When an article is published we post the peer reviewers' comments and the authors' responses online. We also post the versions of the paper that were used during peer review. These are the versions that the peer review comments apply to.

The versions of the paper that follow are the versions that were submitted during the peer review process. They are not the versions of record or the final published versions. They should not be cited or distributed as the published version of this manuscript.

BMJ Open is an open access journal and the full, final, typeset and author-corrected version of record of the manuscript is available on our site with no access controls, subscription charges or pay-per-view fees (http://bmjopen.bmj.com).

If you have any questions on BMJ Open's open peer review process please email info.bmjopen@bmj.com

BMJ Open

Smoking, nicotine and pregnancy 2 (SNAP2) trial protocol: randomised controlled trial of an intervention to improve adherence to nicotine replacement therapy in pregnancy

Journal:	*BMJ Open*
Manuscript ID	bmjopen-2024-087175
Article Type:	Protocol
Date Submitted by the Author:	02-Apr-2024

Complete List of Authors:	Clark, Miranda; University of Nottingham, Centre for Academic Primary Care, School of Medicine<br>Cooper, Sue; University of Nottingham, Centre for Academic Primary Care, School of Medicine<br>Naughton, Felix; University of East Anglia Faculty of Medicine and Health Sciences, School of Health Sciences; University of East Anglia<br>Ussher, Michael; St George's University of London, Division of Population Health Sciences and Education; University of Stirling, Institute of Social Marketing and Health<br>Emery, Joanne; University of East Anglia School of Health Sciences,<br>McDaid, Lisa; University of East Anglia, School of Health Sciences<br>Thomson, Ross; University of Nottingham School of Medicine, Centre for Academic Primary Care<br>Phillips, Lucy; University of Nottingham School of Medicine, Centre for Academic Primary Care<br>Bauld, Linda; University of Edinburgh Division of Medical and Radiological Sciences, Usher Institute of Population Health Sciences and Informatics<br>Aveyard, Paul; University of Oxford Division of Public Health and Primary Health Care, Primary Care Health Sciences<br>Torgerson, David; University of York, York Trials Unit<br>Berlin, Ivan; Hopital Pitie-Salpetriere Service de Pharmacologie, Pharmacology; Université de Lausanne Faculté de biologie et médecine,<br>Lewis, Sarah; University of Nottingham,<br>Parrott, Steve; University of York, Department of Health Sciences<br>Hewitt, Catherine; University of York, Department of Health Sciences<br>Welch, Charlie; University of York, Department of Health Sciences<br>Parkinson, Gill; University of York, York Trials Unit, Department of Health Sciences<br>Dickinson, Anne; University of Nottingham School of Medicine, Centre for Academic Primary Care<br>Sutton, Stephen; University of Cambridge, Department of Public Health and Primary Care<br>Brimicombe, James; University of Cambridge, Primary Care Unit, Department of Public Health and Primary Care; University of Cambridge, Department of Public Health and Primary Care<br>Bowker, Katharine; University of Nottingham School of Medicine, Centre for Academic Primary Care<br>McEwen, Andrew; University College London, Department of Behavioural Science and Health

	Vedhara, Kavita; University of Nottingham School of Medicine, Centre for Academic Primary Care; Cardiff University, School of Psychology<br>Coleman, Tim; University of Nottingham, Division of General Practice
Keywords:	PUBLIC HEALTH, Smoking Reduction, Maternal medicine < OBSTETRICS, RESPIRATORY MEDICINE (see Thoracic Medicine)

SCHOLARONE™
Manuscripts

Smoking, nicotine and pregnancy 2 (SNAP2) trial protocol: randomised controlled trial of an intervention to improve adherence to nicotine replacement therapy in pregnancy

Miranda Clark [1], Sue Cooper [1], Felix Naughton [2], Michael Ussher [3], Joanne Emery [2], Lisa McDaid [2], Ross Thomson [1], Lucy Phillips [1], Linda Bauld [4], Paul Aveyard [5], David Torgerson [6], Ivan Berlin [7], Sarah Lewis [8], Steve Parrott [9], Catherine Hewitt [6], Charlie Welch [6], Gill Parkinson [6], Anne Dickinson [1], Stephen Sutton [10], James Brimicombe [10], Kate Bowker [11], Andy McEwen [12], Kavita Vedhara [13], Tim Coleman [1].

1. Centre for Academic Primary Care, School of Medicine, University of Nottingham, Nottingham, UK
2. Behavioural and Implementation Science Group, School of Health Sciences, University of East Anglia, Norwich, UK
3. Division of Population Health Sciences and Education, St. George's, University of London and Institute of Social Marketing and Health, University of Stirling, Stirling, UK
4. Usher Institute and SPECTRUM Consortium, University of Edinburgh, Edinburgh, UK
5. Nuffield Department of Primary Care Health Sciences, University of Oxford, Oxford, UK
6. York Trials Unit, Department of Health Sciences, University of York, York, UK
7. Department of Medical Pharmacology, Pitié Salpêtrière Hospital-Sorbonne Université, Paris, France
8. Faculty of Medicine and Health Sciences, University of Nottingham, Nottingham, UK
9. Institute of Public Health, University of Cambridge, Cambridge, UK
10. Behavioural Science Group, University of Cambridge, Cambridge, UK
11. Nottingham Clinical Trials Unit, School of Medicine, University of Nottingham, Nottingham, UK
12. Department of Behavioural Science and Health, University College London, London, UK
13. School of Psychology, Cardiff University, Cardiff, UK

For peer review only

For peer review only - http://bmjopen.bmj.com/site/about/guidelines.xhtml

Abstract

Introduction: Smoking in pregnancy is harmful for unborn babies, infants, and women. Nicotine replacement therapy (NRT) is offered as usual stop smoking support in the UK. However, this is often used in insufficient doses, intermittently, or for too short a time to be effective. This randomised controlled trial (RCT) explores whether a bespoke intervention, delivered in pregnancy, improves adherence to NRT and is effective and cost effective for promoting smoking cessation.

Methods and analysis: A two-arm parallel-group RCT for pregnant women aged ≥ 16 years and who smoke ≥1 daily cigarette (pre-pregnancy smoked ≥5), and who agree to use NRT in a quit attempt. Recruitment is from antenatal care settings and via social media adverts. Participants are randomised using blocked randomisation with varying block sizes, stratified by gestational age (<14 weeks or ≥14 weeks) to receive: i) usual care stop smoking support (UC) or ii) UC plus an intervention to increase adherence to NRT, called "Baby, Me and NRT", comprising adherence counselling, automated tailored text messages, a leaflet and website.

The primary outcome is biochemically validated smoking abstinence at or around childbirth, measured from 36 weeks gestation. Secondary outcomes include NRT adherence; other smoking measures; and birth outcomes. Questionnaires collect follow up data which are augmented by medical records information. We anticipate quit rates of 10% and 16% in control and intervention groups respectively (RR = 1.6). By recruiting 1320 participants the trial should have 90% power (alpha = 5%) to detect this intervention effect. An economic analysis will use the Economics of Smoking in Pregnancy (ESIP) model to determine cost-effectiveness.

Ethics and dissemination: Ethics approval was granted by Bloomsbury NHS Research Ethics Committee (21/LO/0123). Findings will be disseminated to the public, funders, relevant practice / policy representatives, researchers, and participants.

Trial registration: ISRCTN16830506

Protocol version 5.0, 10 Oct 2023

For peer review only

Strengths and limitations

- This is the first RCT testing an intervention to increase uptake and adherence to NRT in pregnancy.
- The trial design is explanatory and pragmatic, and so will show whether changes in smoking are due to altered adherence to NRT.
- We report the design of the RCT according to the SPIRIT guidelines.
- Participants are not blind to the treatments and this could cause bias, which is limited by using biochemical verification of abstinence as the primary outcome.
- Obtaining data on smoking abstinence from pregnant people who smoke is difficult; using routine data may ameliorate this.

For peer review only

Introduction

Smoking in pregnancy is still a prevalent public health issue worldwide. For example in the UK, around 7.5% of UK women smoke at childbirth[1]. Smoking in pregnancy is associated with negative outcomes for both women and babies, but is preventable, and women who stop smoking in pregnancy are less likely to have premature or low birth weight infants.[2] Compared with mothers who do not smoke, those who continue smoking in pregnancy have heightened risks of placental abruption, miscarriage, still birth and ectopic pregnancy.[3] Children born to parents who smoke are more likely to start smoking themselves[4] and tobacco smoking is a major risk factor for six of the eight leading causes of death worldwide.[5]

Pregnancy is probably a life event that most motivates people to try stopping smoking; around 50% of women who smoke stop during gestation; many others try but fail.[6] A key reason is that nicotine withdrawal symptoms and smoking urges experienced on stopping smoking are difficult to tolerate. Nicotine Replacement Therapy (NRT) provides nicotine without exposing users to toxins like tar, cyanide and carbon monoxide, and so safely helps ameliorate withdrawal and smoking urges. In the UK, National Institute for Health and Care Excellence (NICE) recommended NRT use in pregnancy since 2010[7,8] and is now a central component of routine clinical practice there.[9,10,11] However, although NRT is effective in general[12], it appears to work less well in pregnancy[13], probably because pregnant women do not use it consistently, for long enough or in sufficient doses. In trials enrolling pregnant smokers, only 7% to 30% finished courses of NRT[13], and of pregnant smokers prescribed NRT by UK GPs, only 30% were supplied it for longer than 2 weeks[11] and such short NRT courses are ineffective. In contrast, non-pregnant smokers enrolled in smoking cessation trials report up to 94% adherence levels.[14]

It is very likely that for NRT to work in pregnancy, higher nicotine doses and dosing consistency are needed than are currently used. In pregnancy nicotine metabolism accelerates[15,16] resulting in NRT generating lower blood nicotine concentrations. Additionally, research suggests that many pregnant women struggle to use NRT consistently or for sufficiently long, due to concerns about nicotine safety and a lack of belief in the need of NRT to quit smoking. These are influenced by erroneous lay beliefs which contribute to idiosyncratic NRT usage patterns and NRT not being used as advised [17,18] Such erratic NRT use can be compounded by inaccurate advice on nicotine safety from friends, family, and even health professionals, exacerbating women's uncertainties about whether and how to use NRT.[19] Both stop smoking practitioners[20] and pregnant women[21] believe that by consistently countering such misinformation, improvements could be made in the number of successful quit attempts. Poor adherence to, and intermittent use of NRT, very likely reduces the chances of smoking cessation in pregnancy, limiting the health benefits that could accrue from optimal use of this treatment.

If better adherence to NRT is not more harmful to the fetus than smoking and helps more pregnancies become smokefree whilst avoiding smoking-related harms, encouraging adherence to NRT would clearly be ethical. There is no biological rationale for suspecting that NRT could be more harmful than smoking in pregnancy. Throughout the 2000s, based on the logical belief that smoking-related harms in pregnancy are unlikely to be due solely to nicotine, there has been expert consensus for using NRT to stop smoking in pregnancy.[22] NRT in pregnancy is not recommended for 'never smokers', but NRT used *instead* of smoking is very likely to be safer. In the unlikely event of unexpected nicotine-attributable fetal harm(s) occurring, one would expect these to be vastly outweighed by benefits from smoking cessation following effective NRT use. A Cochrane review found no evidence that,

For peer review only - http://bmjopen.bmj.com/site/about/guidelines.xhtml

for pregnancy outcomes, NRT harms either women or their babies, although analyses were generally underpowered to detect moderate sized effects[13] and observational studies are not sufficiently robust to add to these findings.[23] However, compared to smoking, NRT has an apparently protective effect on infant development; at 2 years old, infants in the largest RCT of NRT in pregnancy[24], born to women randomised to NRT rather than placebo, were more likely to have unimpaired development[25].

Rationale

In a National Institute for Health Research-funded programme, we developed '*Baby, Me & NRT*' (BMN), an intervention to improve adherence to NRT during pregnancy.[26] In cohort studies, we optimised and monitored impacts of BMN and this RCT explores whether BMN helps pregnant women stop smoking and increases adherence to NRT. In this paper we report the protocol of SNAP2 according to the SPIRIT guidelines.[27]

Methods and analysis

SNAP2 is a multi-centre, parallel group, individually randomised controlled trial of the 'Baby, Me & NRT' (BMN) intervention integrated with usual smoking cessation support during pregnancy versus usual smoking cessation support alone.

This RCT was originally envisaged solely as a 'proof of concept' study that aimed to detect whether BMN increased NRT adherence. If so, a separate RCT was planned to explore BMN effects on cessation. However, due to NHS service provision changes and the Covid pandemic, the funder accepted that, following demonstration of 'proof of concept' from a pilot phase, efficacy could be tested by recruiting sufficient participants to SNAP2. Below, we indicate which methodological features were used only in the pilot, and the sample size section explains the basis for progression from pilot to full trial.

Objectives

Primary objective

To determine whether, when added to usual NHS cessation support, the 'Baby, Me & NRT' intervention increases smoking abstinence during pregnancy, as measured in late pregnancy or at childbirth, with exhaled carbon monoxide and/or saliva samples used to validate self-reported abstinence.

Secondary objectives

In ***all*** participants, to compare between intervention and usual care groups:

i) Reported smoking abstinence at 28 days after a quit date

ii) Reported smoking abstinence at both 28 days after a quit date, and in late pregnancy / childbirth with and without validation in late pregnancy

iii) The number of days of NRT use in the first 28 days after a quit date.

iv) Mean daily nicotine dose in the first 7 days after a quit date ('intensity' of NRT use)

v) Adverse pregnancy outcome rates

In ***intervention group*** participants:

vi) To assess engagement with BMN intervention components.

For peer review only - http://bmjopen.bmj.com/site/about/guidelines.xhtml

Economic
vii) To investigate cost-effectiveness of the BMN intervention.

Pilot phase objectives
In ***all*** pilot phase participants, to compare between intervention and usual care groups:

viii) Urges to smoke and tobacco withdrawal symptoms at Day 7 after a quit date

ix) NRT concerns and necessity beliefs at Day 28.

In pilot phase ***intervention group*** participants and practitioners only:

x) To assess fidelity of BMN intervention delivery

Other objectives (pilot phase only)
To compare biochemically measured nicotine exposure before and after exposure to BMN intervention.

Inclusion criteria

People are eligible if aged ≥ 16; pregnant and <25 weeks' gestation; they smoked ≥ 5 daily cigarettes before pregnancy (currently smoking ≥1 daily cigarette) and are referred for or receiving antenatal care. Participants must have sufficient understanding of English to give informed consent; agree to try stopping smoking with NRT within 14 days, to receive and send SMS text messages, and install the trial's data collection app on their smartphone.
.

Exclusion criteria

They are ineligible if already using NRT or are enrolled in a smoking cessation study, NHS stop smoking support or a cessation-orientated text message service, or they intend to continue using e-cigarettes or have contraindications to NRT.

Recruitment

Participants will be identified from:

i) National Health Service (NHS) clinical settings, by poster, direct contact from researchers (face-to-face or distanced) and online, with adverts in NHS digital spaces.
ii) Online, outside of NHS settings

NHS settings
These can be hospital antenatal care, general practice, community midwifery or stop smoking service settings. Researchers may identify potential participants from medical records, contacting them by letter, telephone, email or text before appointments, and including QR code / links to or paper copies of a Patient Information Sheet (PIS). They may also approach pregnant people attending clinics asking them to complete an eligibility screening questionnaire or give them a summary leaflet which contains links / QR codes leading to the PIS. Depending on the setting, researchers may consent those who are interested and eligible, or they may pass contact details to the trial team to enable consent to be received by them.

Posters describing the trial will be displayed in clinical areas or in appropriate NHS digital spaces; these will include QR codes or links leading to the PIS, and to an online version of the screening questionnaire, following which eligible and interested people will be invited to

For peer review only - http://bmjopen.bmj.com/site/about/guidelines.xhtml

leave contact details in a secure RedCap database hosted by the University of Nottingham. The trial team can then access contact details and contact potential participants directly.

Online, outside of NHS settings

Google or social media (e.g. Facebook, Instagram,) adverts will be targeted at those who smoke and are pregnant. Embedded links will lead to a study information webpage and to an eligibility questionnaire, and those who are potentially eligible will be asked to enter contact details, as above.

Interested, eligible potential participants will be given at least 24 hours to consider the PIS before discussion with a researcher and informed consent received. Discussion and documentation of consent could be by face-to-face (using ‘wet ink’) or ‘distanced’ using either an online form or by telephone. If the online form is used, eligible potential participants will be sent this via a link and complete this during a telephone conversation with a researcher. Consent via telephone is similar but in this case the consent form will be generated by the researcher from the research database, following a strict protocol, then signed copies will be shared with the participant. For all consent methods, a letter will be sent to the participant’s GP informing them of the enrolment.

Randomisation and Blinding

After informed consent, participants’ baseline data will be collected before randomising them to either study arm with the York Trials Unit’s (YTU) web-based system. The randomisation schedule will be computer-generated, with pseudo-random code using random permuted blocks of randomly varying size and stratified by gestational age (<14 weeks or ≥14 weeks). Immediately afterward, the trial office receives email confirmation of treatment allocation. Participants and those delivering interventions will be aware of treatment allocations, but, researchers who collect data will be blinded. To prevent BMN components being inadvertently delivered to the usual care group, two separate teams of stop smoking practitioners (SSPs) will be used to deliver smoking cessation support.

Interventions

Control

Usual Care for smoking cessation, following the National Centre for Smoking Cessation Training (NCSCT) standard treatment programme[28] and which comprises:

i) Help setting a quit date
ii) Up to 6 telephone or video call counselling sessions
iii) An offer of NRT as patch, short-acting NRT or both together (‘dual NRT’). [9,10]

Before Quit Date (QD): SSPs assess participants’ suitability for NRT in terms of, cautions or contraindications, other prescription medications and health issues, and counsel participants on how best to use NRT as per the NCSCT guidelines. Guidelines advise the ‘not a puff’ rule where NRT should only be used when not smoking. If there is doubt about NRT safety, participants’ GPs are consulted to assess their medical suitability to using NRT products. Participants are mailed a 14-day supply of their chosen NRT product(s) and are instructed to start this on the QD.

After Quit Date: Practitioners offer counselling appointments between the QD and Day 3 and on Days 7, 14, 21 and 28 after the QD. SSPs ask about withdrawal, use of NRT and experience of nicotine side effects, and advise on effective NRT use. At Day 14, participants still using NRT are offered a further 14-day supply.

NRT: Advice given to use dual therapy (one long acting and one short acting product) with dose titrated to the number of cigarettes smoked per day. For those who cannot tolerate patches, 2 short acting products can be substituted with advice on how to ensure round the

For peer review only - http://bmjopen.bmj.com/site/about/guidelines.xhtml

clock coverage. Participants may choose from the following products supplied in their original packaging; all have UK licenses for use in pregnancy:

Patches: Daily Nicorette 16-hr (15mg or 25mg) or NiQuitin clear 24 hr (14mg or 21 mg).

Short acting NRT: Nicorette Inhalator 15mg (max 6 cartridges / day); Nicorette Cools Lozenges 2mg or 4 mg (max 8-12 lozenges / day); Nicorette QuickMist mouthspray 1mg/spray (Maximum: 2 sprays at a time; 4 sprays / hour; 64 sprays / day).

For both trial groups, support beyond the 28 days during which trial interventions are delivered, is provided by locally available UC NHS support.

Intervention

The 'Baby, Me & NRT' (BMN) intervention is offered alongside UC (described above) and integrated into an identical schedule. BMN is described in detail elsewhere[26] and comprises of tailored behavioural support designed to encourage adherence to NRT and increase quit rates during pregnancy. The main components are:

Counselling: Participants are asked to complete a short questionnaire to assess their concerns and necessity beliefs regarding use of NRT in pregnancy[29]; the latter are views on how worthwhile NRT might be to participants. The number of counselling sessions mirror usual care, but the content addresses individual concerns and beliefs about the safety of nicotine and the efficacy of nicotine replacement products The first counselling session is on average 10 minutes longer than those delivered in usual care, is delivered by video call where possible and addresses individual concerns and beliefs about the safety of nicotine. To ensure advice is as personalised as possible, SSPs respond to key concerns and necessity beliefs recorded on questionnaires. Participants are advised, if needed, to use a patch and short-acting NRT preparation until childbirth, and during brief lapses to smoking (of up to 14 days), provided they still try to quit. To avoid morning cravings, 24-hour NRT patches may be left on overnight, and support is aimed at maintaining adherence to NRT. Follow up calls are mainly by telephone; these again focus on addressing concerns about nicotine, using sufficient NRT and on not stopping this during brief smoking lapses, in addition to the UC advice.

Leaflet and website: These reinforce key NRT adherence-enhancing messages using video animations and careful wording. Additionally, there are video clips from experts and/or written experiences with NRT from other pregnant women.

Text messages: For up to 30 days, we send personally tailored, automated texts, based on participants' smoking behaviours and NRT-related beliefs. These aim to support participants' abstinence; encourage using sufficient NRT to control withdrawal symptoms and cravings; counter intentional non-adherence to NRT (e.g. due to nicotine-related concerns) and provide prompts or reminders to prevent non-intentional non-adherence to NRT (e.g., forgetting).

Staff delivering interventions

All SSPs delivering support are trained to the recognised NCSCT standard required for delivering UC stop smoking support in the UK NHS.[26] All intervention group participants are counselled by specially trained SSPs working within the research team, who deliver the Baby, Me and NRT, components integrated into usual care. Control group counselling is provided by either a separate group of research team SSPs or by NHS providers responsible for providing locally available Usual Care stop smoking support.

For peer review only - http://bmjopen.bmj.com/site/about/guidelines.xhtml

Data Collection

Table 1 shows all participant data collection at time points outlined below and indicates how intervention delivery fits with this. We indicate which measures will be used for research purposes in the pilot only. Figure 1 is a study flow diagram.

Table 1: Schedule of data collection and intervention delivery time points

	Time point								
**Data collected**	**Pre-baseline (consent)**	**Baseline[1]**	**Pre-Quit Date**	**Day 1-3**	**Day 7**	**Day 14**	**Day 21**	**Day 28**	**Delivery (Week 36 gestation)**
**Informed consent**	X[2]								
**Smoking status/ CPD/ use of ecigs**		X			P[3]			X	X
**Cravings & tobacco withdrawal**		P			P			P	
**NRT concerns & necessity beliefs**		P						P	
**Saliva samples**		P			P				X
**Exhaled CO**		X							X
**NRT adherence data**					P			X	X
**Reported engagement with intervention**								X	
**NicUse app data collection**				←	—	—	—	→	
**Medical records data**									X
**Intervention Delivery (both trial arms)**									
**Counselling from SSPs offered**			X	X	X	X	X	X	
**NRT dispatch**			X		X	X	X	X	

[1] Randomisation follows baseline data collection
[2] X = data collected in both pilot and full trial phase
[3] P = data collected only in pilot phase

For peer review only

Figure 1: Study Flowchart

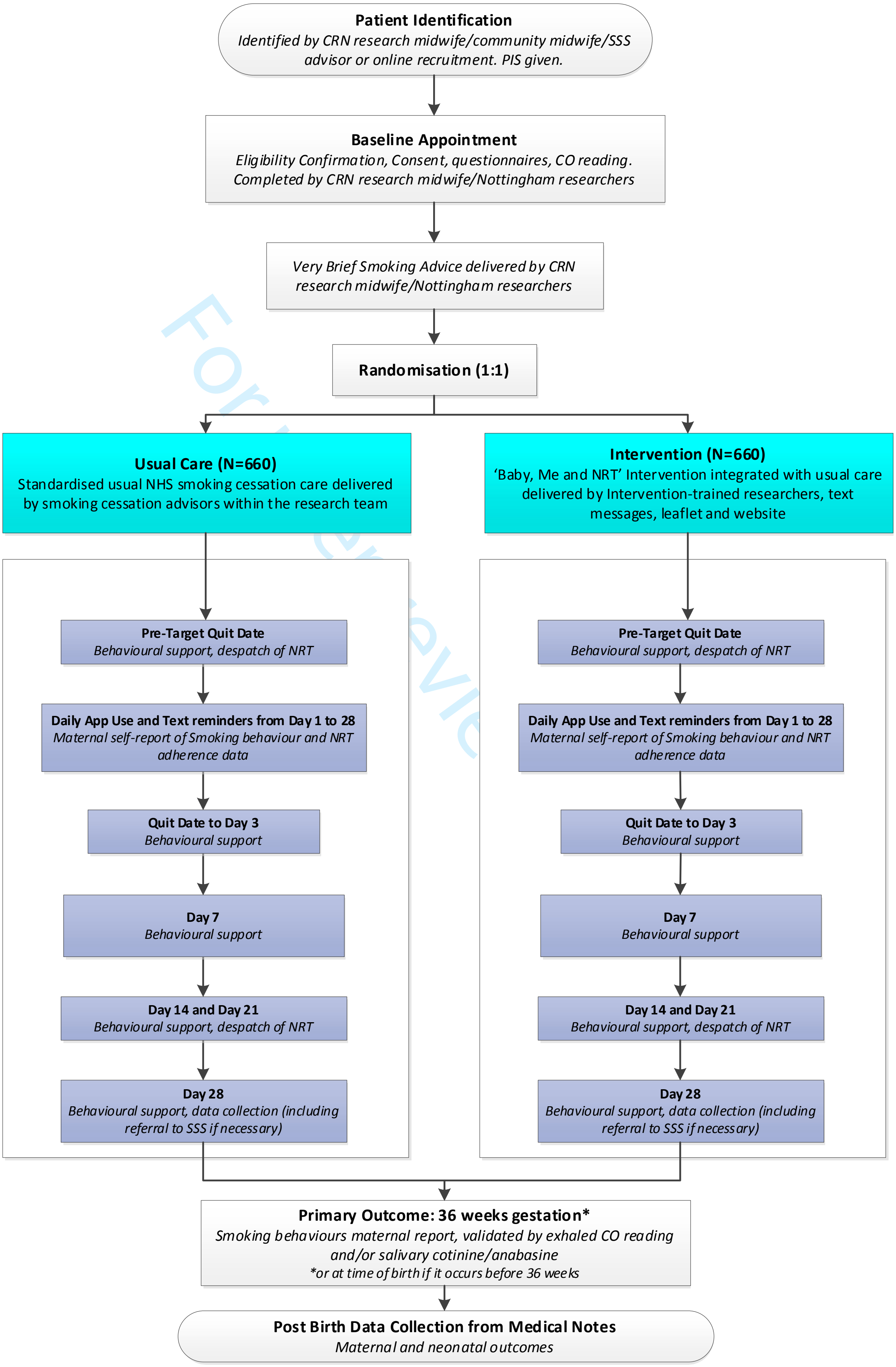

For peer review only - http://bmjopen.bmj.com/site/about/guidelines.xhtml

For peer review only

Baseline

We will ask participants about demographics, gestation, estimated date of delivery, partner smoking status, whether they smoked in previous pregnancies, nicotine dependence[30], current and pre-pregnancy smoking behaviours, prior experience using NRT, smoking beliefs, urges to smoke (cravings) and tobacco withdrawal symptoms.[31] Where possible, those recruited in person will also provide an expired air carbon monoxide (CO) reading at baseline. Researchers will also help participants to install the 'NicUse' smartphone app from Google Play and Apple Store [32], a bespoke app developed for SNAP2 on which applicants record daily smoking, e-cigarette and NRT use (see below) during the 28 -day intervention period. We will seek contact details for participants' GPs and check with them if there is any reason why a participant should not be enrolled.

Pilot phase only: All participants were sent an online questionnaire measuring concerns and necessity beliefs about NRT.[29] Those recruited in person were asked (at baseline) for a saliva sample and, if possible a CO reading given a self-return kit for collection and return of another saliva sample on Day 7 of their quit attempt. Those recruited online or remotely, were mailed two self-collection kits with instructions for returning one saliva sample immediately, and another on Day 7 of their quit attempt. Prior to collecting samples, we asked participants, verbally or by questionnaire in the postal return kit, when they last smoked, used an e-cigarette, if they are using NRT and which type(s) and, when this was last used.

Follow up

After baseline, apart from data collection by app and from routine sources, the primary data collection mode is by online questionnaires, with links texted or emailed to participants. If there is no response, first a reminder text and/or email will be sent, and then participants will be phoned, hard copy questionnaires with reply paid envelopes will be posted as a final option.

Daily NicUse recording of behaviours (between baseline & Day 28)

NicUse works on Android or Apple smartphones. Participants will be asked to use the app daily to record smoking behaviour, NRT and/or e-cigarette use. If participants miss reporting for one or two days, they can record these data retrospectively. Participants receive text messages to prompt completion of the app. The survey on NRT will ask participants to record patch and short-acting NRT use, and the number of units of short-acting NRT consumed, which allow us to calculate their daily nicotine dose[33]. Compared to questionnaires, NicUse facilitates more complete collection of NRT adherence data, with more robust face validity, and is less likely to overestimate adherence than questionnaires[33].

Day 7 after quit date (pilot phase only)

We were asked if a quit attempt was made, if any NRT had been used since the quit date (QD) and if so, on how many days, which types, and if short-acting NRT is used, how many lozenges, cartridges or sprays were used. We also asked about current smoking, e-cigarette use, urges to smoke (cravings) and tobacco withdrawal symptoms. As soon as possible after Day 7, saliva samples (see above) were collected from participants. After the pilot, we discontinued data collection at this time point, as this information was only required for pilot phase outcomes.

Day 28 after quit date

We will use the same questions as in the pilot phase-only Day 7 follow up but will ask about the previous 28 (not 7) days, and we will ask the intervention group about BMN components (e.g. texts and website). Participants who do not provide information on smoking status or NRT use by app or questionnaire methods will be sent a text about NRT use since the quit date, and asked to reply directly.

Pilot phase only: Participants were asked to re-complete the NRT concerns and necessity beliefs measure they first completed at baseline.[29]

For peer review only - http://bmjopen.bmj.com/site/about/guidelines.xhtml

36 weeks gestation or delivery (if earlier)
We will ask participants about smoking, adherence to NRT since 28-day follow up and use of NHS stop smoking support. We will send non-responders a direct-reply text message asking about smoking in the previous 7 days.

Routine data collected from medical records
NHS hospitals collect smoking status and exhaled carbon monoxide from every woman from week 36 of gestation onwards and we will collect these data from NHS records. We will also extract maternal and fetal pregnancy outcome data from medical records. In NHS hospitals which are sites, research staff will collect these data; otherwise, the research team contact relevant NHS staff to ask for this information. Where birth outcome data cannot be obtained from records, using methods outlined above, we will ask participants to provide birth weight, gestation at delivery, and whether they underwent caesarean section or the baby was admitted to special care or had any congenital abnormality, and whether they were a smoker or non-smoker at delivery.

Validation of smoking abstinence
For participants who report 7-day smoking abstinence at 36 weeks or later, we will collect saliva samples as they attend hospital, at home visits, or postally (see above). Before giving samples, participants will be asked about any recent smoking or vaping or NRT use.

Financial incentives encouraging data return
To recognise time taken for study participation, participants will receive up to £50 in 'Love to Shop' gift cards which cannot be redeemed for tobacco or alcohol. To receive maximum remuneration participants will need to submit all adherence app data; they will receive 50p for each daily app report, and an additional £1.50 for supplying a continuous full week's reports, plus an additional £5 if they report for all 28 days after their quit date (maximum total £25). Further gift cards will be given to women if they provide requested questionnaire data and validation saliva samples.

Fidelity assessment (pilot phase only)
We will audio record all initial intervention group consultations, selecting a random sample for further scrutiny. Two researchers will listen to the selected audio recordings, independently rating the completeness of intervention delivery against a fidelity checklist which lists key components of the BMN intervention, and inter-rater reliability between researchers will be determined. We will store recordings on a secure University of Nottingham server for a maximum of seven years.

Data Management
Each participant will be assigned a unique study identification number allocated at consent to identify their data and biological samples. Personal identifiers (name, email address and phone number) will be stored in a password-protected computer database accessible only by the researchers. Data will be entered into a REDCap database where possible, but paper CRFs may be used as source data and entered by researchers onto the database. Information submitted by participants via the NicUse app is stored as pseudonymised data on Amazon cloud. Saliva samples will be collected by researchers or by participants and send directly to ACM labs for storage and analysis at the end of the trial.

All electronic data will be securely stored at the University of Nottingham for 15 years after which it will be destroyed. Data Management will be lead by York Trials Unit, with the support of the Trial Manager in University of Nottingham as detailed in the Data Management Plan.

Outcomes

Primary outcome

Reported smoking abstinence in late pregnancy or around childbirth, with appropriate biochemical validation.

For peer review only - http://bmjopen.bmj.com/site/about/guidelines.xhtml

Secondary outcomes

1. Reported smoking abstinence at *both* 28 days and in late pregnancy or at childbirth, with and without appropriate biochemical validation in late pregnancy.
2. Reported smoking abstinence for 24hrs and 7 days at 28 days.
3. Reported number of days NRT is used in the first 28 days following a quit date.
4. Reported mean daily nicotine dose in the first 7 days of quitting ('intensity' of NRT use)
5. Engagement with BMN intervention components

Pilot phase outcomes

6. Urges to smoke, 'cravings', and tobacco withdrawal symptoms.
7. NRT concerns and necessity beliefs at baseline and Day 28.
8. Fidelity of intervention delivery as measured against fidelity checklist.

Other outcomes

9. Saliva cotinine concentration
10. Number of days NRT use between a quit date and the end of pregnancy.
11. Exhaled carbon monoxide (CO) concentration
12. Birth weight
13. Low birth weight (<2500g)
14. Gestational age at birth
15. Maternal or fetal death (stillbirth or miscarriage).
16. Caesarean section delivery
17. Neonatal intensive care admission
18. Congenital anomaly

Sample size and justification

Design changes: Originally, we planned SNAP2 as an RCT to test the extent to which 'Baby, Me and NRT' (BMN) did or did not increase adherence to NRT; adherence to NRT was intended as the primary outcome and smoking outcomes as secondary. If study findings gave a sufficiently positive 'signal' for an effect on NRT adherence, we planned a second RCT to test whether BMN had positive effects on smoking cessation. However, due to the Covid pandemic and NHS service provision changes, the funder agreed that BMN efficacy for smoking abstinence would be better investigated by instead simply using a measure of smoking behaviour as the trial primary outcome and increasing the SNAP2 sample size. This was dependant on a successful pilot phase of the trial in which i) BMN demonstrated a sufficiently large 'signal' that impacts on adherence to NRT, and ii) explanatory trial outcomes data were collected prior to being discontinued in the more pragmatic 'full' trial . Below we detail how, in the SNAP2 pilot phase, the impact of BNM on adherence to NRT assessed, and progression decided upon.

Assessment of BMN's potential effect on NRT adherence': For the original SNAP2 RCT, we had defined a clinically important effect as BMN increasing NRT use by 21%. Data from a previous study indicated that, if the control group was offered NRT for 28 days, they were likely to use this on a mean of 7 days[34], so a 21% increase represented an extra 1-2 days of NRT use.

We used a one-sided confidence interval approach[35] to assess whether or not the pilot phase SNAP2 trial 'signal' for the impact of BMN on NRT adherence was consistent with assumed effects. Using a confidence interval approach, we calculated that for the checkpoint analysis to produce an upper limit of a one-sided 80% or 90% confidence interval which excludes the estimate of effect, assuming the treatment estimate from the checkpoint assessment was zero or less would require 34 or 54 in the analysis. A trial statistician estimated BMN efficacy using data from the 49 participants followed up to the primary outcome point. The point estimate, and the upper confidence limit (whether 80% or 90%)

For peer review only - http://bmjopen.bmj.com/site/about/guidelines.xhtml

was greater than the pre-specified clinically relevant effect size of 1.21. The funder deemed this sufficient demonstration of potential efficacy for the pilot to progress to a full trial.

Full trial sample size estimate: To determine the sample size for the full trial, we assume a control group quit rate of 10%, consistent with similar UK studies[34;36,37], and seek to detect an absolute increase in risk of abstinence of 6% (corresponding to a risk ratio (RR) of 1.6). We think this is a reasonable effect size to seek as all previous trials of NRT used by pregnant women tested use of only one NRT product and these show a relatively weak, imprecise effect (RR 1.37, 95% CI 1.08 to 1.74),[13] with smaller point estimates seen in the least biased studies (RR 1.21, 95% CI 0.95 to 1.55).[13] However, in non-pregnant smokers where adherence to NRT is much stronger, depending on the type of NRT used, Cochrane review point estimates for NRT efficacy range between 1.49 (for gum) and 2.02 (inhalator)[38] and dual NRT (patch + fast acting) is more effective than single product use, with an OR for abstinence with NRT of 1.25 (dual versus single product NRT use).[12] We aim to recruit 1,320 participants, providing a minimum of 90% power for a two-sided test of size 5%, assuming the control group quit rate is at least 10%.

Analyses

Statistical analysis will be conducted when the trial ends using Stata/MP v18 or later unless specified otherwise. All analysis software used including any community contributed software will be explicitly cited in any publication of the trial results. Significance tests will be two-sided at the 5% level unless specified otherwise. Point estimates will be presented with their associated 95% confidence intervals. Full analyses will be detailed in a statistical analysis plan (SAP), finalised prior to the end of data collection, and which will be reviewed by the TSC. A CONSORT flow diagram will be provided to display the flow of participants through the study. Baseline data will be summarised descriptively by group, for all randomised and for all those who are included in the primary analysis. No formal statistical comparisons of group differences at baseline will be conducted. Continuous measures will be reported as means and standard deviation, while the categorical data will be reported as counts and percentages.

Assessment of efficacy

The primary efficacy endpoint is reported abstinence in late pregnancy or around childbirth, validated by appropriate biochemical measures (binary – abstinent or non-abstinent). The primary analysis model will include all randomised participants as part of the groups to which they were allocated, with any missing primary endpoints imputed as being non-abstinent. We will compare abstinence between treatment groups using logistic regression, with a fixed effects of treatment group, gestation at baseline (the stratification factor), and other predictive baseline covariates (pre-specified in the SAP). In addition to the point and interval estimate of the OR for allocation, we will use the fitted model to obtain estimates of treatment effects on both the relative risk and risk difference scales. Binary secondary outcomes (e.g. other abstinence outcomes) will be analysed in a broadly similar manner to the analysis of the primary outcome. Secondary outcomes including adherence to NRT, NRT adherence intensity, cotinine (smoking exposure), necessities/concerns and birth outcomes will be compared between arms using appropriate generalised linear models. All other outcomes will be summarised descriptively by randomised arm. If the relevant data are sufficiently complete, we will undertake exploratory analyses to decompose the total effect of allocation on the primary outcome into indirect effects (i.e. those mediated by improved NRT adherence/usage) and direct effects (i.e. those not mediated by improved NRT adherence/usage).

Procedures for missing, unused and spurious data

We will assume that participants with missing smoking status are smoking, in line with the Russell Standard, meaning people who do not provide data are assumed to be smoking[39]. Similarly, participants not providing a NRT usage response(s) will be assumed to be not using NRT in the corresponding time period. We will investigate the sensitivity of results from

For peer review only - http://bmjopen.bmj.com/site/about/guidelines.xhtml

the main analyses to departures from these strict missing not at random (MNAR) assumptions, by imputing these missing data under a range of missing at random (MAR) and MNAR scenarios.

Safety

The study tests a behavioural intervention aimed at optimising NRT use.[26] As this is a standard NHS treatment, we do not anticipate any harm being caused and there is no adverse event monitoring.

Economic Analysis

The economic analysis will determine the cost-effectiveness using a lifetime time horizon. To estimate long term benefits, costs and cost-effectiveness, and potential longer-term cost savings we will use the Economics of Smoking in Pregnancy (ESIP) model, a bespoke, dynamic economic model designed specifically for valuing smoking cessation in pregnancy in economic terms.[40;41] Smoking cessation rates from the trial and costs of intervention delivery and usual care will be used as ESIP inputs. As SNAP 2 has a short follow up period, we will not use measures of participants' Quality of Life as one would not expect changes in response to the SNAP2 intervention within the short study timeframe, and any QoL changes resulting from smoking cessation would likely be variation in QoL reflecting physiological changes in pregnancy.

Ethics and dissemination

Ethics approval has been granted by the Bloomsbury NHS Research Ethics Committee (21/LO/0123). The findings will be disseminated to the public, the funders, relevant practice and policy representatives and other researchers. A data sharing agreement has been published; once the trial has finished and the main trial paper has published, a fully anonymised trial data set will be available on reasonable request from York Trials Unit.

Discussion

We completed recruitment of 264 participants, 100% of target sample size, to the pilot study in October 2022. The main study aims to recruit 1320 participants by December 2025 with current recruitment on track to achieve this, and follow up to continue for a further 10 months with last participant last visit due in October 2026.

Due to the pandemic, recruitment via traditional routes became problematic. In response to these difficulties, we have successfully recruited via paid social media advertising. Of the first 265 participants 185 (69.8%) were recruited via hospital antenatal settings and 80 (30.2%) via online recruitment routes.

Patient and public involvement Statement

A User and Public Involvement Advisory Group has been set up to for our N-READY research programme; the current trial is part of this programme. This advisory group is made up of women who currently smoke, who have smoked at any point during pregnancy, or who are of childbearing age and smoke / are ex-smokers. This Advisory Group contributes to all stages of the study, from reviewing study related documentation and materials to dissemination of research findings. Intervention development, via previous workstreams of the N-READY programme, has already been heavily informed by this group as well as by interviews with women who smoke (or have smoked) during pregnancy.

For peer review only - http://bmjopen.bmj.com/site/about/guidelines.xhtml

References

1. NHS Digital. Statistics on Women's Smoking Status at Time of Delivery: England, Quarter 2, 2023-24. 2024; https://digital.nhs.uk/data-and-information/publications/statistical/statistics-on-women-s-smoking-status-at-time-of-delivery-england/statistics-on-womens-smoking-status-at-time-of-delivery-england-quarter-2-2023-24. Accessed 08/03/2024.
2. Chamberlain C, O'Mara-Eves A, Porter J, et al. Psychosocial interventions for supporting women to stop smoking in pregnancy. *Cochrane Database of Systematic Reviews.* 2017(2).
3. Cnattingius S. The epidemiology of smoking during pregnancy: smoking prevalence, maternal characteristics, and pregnancy outcomes. *Nicotine Tob Res.* 2004;6(S2):S125-S140.
4. Leonardi-Bee J, Jere ML, Britton J. Exposure to parental and sibling smoking and the risk of smoking uptake in childhood and adolescence: a systematic review and meta-analysis. *Thorax.* 2011;66(10):847-855.
5. World Health Organization. *Report on the Global Tobacco Epidemic 2008—the mpower package.* Geneva: World Health Organization;2008.
6. Cooper S, Orton S, Leonardi-Bee J, et al. Smoking and quit attempts during pregnancy and postpartum: a longitudinal UK cohort. *BMJ Open.* 2017;7(11).
7. National Institute for Health and Clinical Excellence. *Quitting smoking in pregnancy and following childbirth. NICE Public Health Guidance 26.* London2010. PH26.
8. National Institute for Clinical Excellence. Tobacco: preventing uptake, promoting quitting and treating dependence. NICE guideline [NG209]. https://www.nice.org.uk/guidance/ng209/chapter/Recommendations-on-treating-tobacco-dependence-in-pregnant-women#providing-support-to-stop-smoking. 2021.
9. Fahy SJ, Cooper S, Coleman T, Naughton F, Bauld L. Provision of smoking cessation support for pregnant women in England: results from an online survey of NHS stop smoking services for pregnant women. *BMC Health Services Research.* 2014;14(1):107.
10. Cooper S, Orton S, Campbell K, et al. Attitudes to E-Cigarettes and Cessation Support for Pregnant Women from English Stop Smoking Services: A Mixed Methods Study. *International Journal of Environmental Research and Public Health.* 2019;16(1):110.
11. Dhalwani NN, Szatkowski L, Coleman T, Fiaschi L, Tata LJ. Prescribing of nicotine replacement therapy in and around pregnancy: a population-based study using primary care data. *Br J Gen Pract.* 2014;64(626):e554-560.
12. Lindson N, Chepkin SC, Ye W, Fanshawe TR, Bullen C, Hartmann-Boyce J. Different doses, durations and modes of delivery of nicotine replacement therapy for smoking cessation. *Cochrane Database Syst Rev.* 2019;4:CD013308.
13. Claire R, Chamberlain C, Davey MA, et al. Pharmacological interventions for promoting smoking cessation during pregnancy. *Cochrane Database of Systematic Reviews.* 2020(3).
14. Hollands GJ, Sutton S, McDermott MS, Marteau TM, Aveyard P. Adherence to and consumption of nicotine replacement therapy and the relationship with abstinence within a smoking cessation trial in primary care. *Nicotine Tob Res.* 2013;15(9):1537-1544.
15. Bowker K, Lewis S, Coleman T, Cooper S. Changes in the rate of nicotine metabolism across pregnancy: a longitudinal study. *Addiction.* 2015;110(11):1827-1832.

For peer review only

16. Dempsey D, Jacob P, III., Benowitz NL. Accelerated metabolism of nicotine and cotinine in pregnant smokers. *Journal of Pharmacology & Experimental Therapeutics.* 2002;301(2):594-598.
17. Bowker K, Campbell KA, Coleman T, Lewis S, Naughton F, Cooper S. Understanding pregnant smokers' adherence to nicotine replacement therapy during a quit attempt: a qualitative study. *Nicotine & Tobacco Research.* 2015.
18. Campbell K, Coleman-Haynes T, Bowker K, Cooper SE, Connelly S, Coleman T. Factors influencing the uptake and use of nicotine replacement therapy and e-cigarettes in pregnant women who smoke: a qualitative evidence synthesis. *Cochrane Database Syst Rev.* 2020;5:CD013629.
19. Thomson R, McDaid L, Emery J, et al. Knowledge and Education as Barriers and Facilitators to Nicotine Replacement Therapy Use for Smoking Cessation in Pregnancy: A Qualitative Study with Health Care Professionals. *International Journal of Environmental Research and Public Health.* 2019;16(10):1814.
20. Fergie L, Campbell KA, Coleman-Haynes T, Ussher M, Cooper S, Coleman T. Stop smoking practitioner consensus on barriers and facilitators to smoking cessation in pregnancy and how to address these: A modified Delphi survey. *Addictive Behaviors Reports.* 2019;9:100164.
21. Fergie L, Coleman T, Ussher M, Cooper S, Campbell KA. Pregnant Smokers' Experiences and Opinions of Techniques Aimed to Address Barriers and Facilitators to Smoking Cessation: A Qualitative Study. *International Journal of Environmental Research and Public Health.* 2019;16(15):2772.
22. Benowitz NL, Dempsey DA, Goldenberg RL, et al. The use of pharmacotherapies for smoking cessation during pregnancy. *Tobacco Control.* 2000;9 Suppl 3:III91-III94.
23. Taylor L, Claire R, Campbell K, et al. Fetal safety of nicotine replacement therapy in pregnancy: systematic review and meta-analysis. *Addiction.* 2020.
24. Coleman T, Cooper S, Thornton JG, et al. A randomized trial of nicotine-replacement therapy patches in pregnancy. *New England Journal of Medicine.* 2012;366(9):808-818.
25. Cooper S, Taggar J, Lewis S, et al. Effect of nicotine patches in pregnancy on infant and maternal outcomes at 2 years: follow-up from the randomised, double-blind, placebo-controlled SNAP trial. *The Lancet Respiratory Medicine.* 2014;2(9):728-737.
26. McDaid L, Emery J, Thomson R, et al. Development of "Baby, Me, & NRT": A Behavioral Intervention to Improve the Effectiveness of Nicotine Replacement Therapy in Pregnancy. *Nicotine & tobacco research : official journal of the Society for Research on Nicotine and Tobacco.* 2023;25(11):1770-1780.
27. Chan AW, Tetzlaff Jm Fau - Gøtzsche PC, Gøtzsche Pc Fau - Altman DG, et al. SPIRIT 2013 explanation and elaboration: guidance for protocols of clinical trials. (1756-1833 (Electronic)).
28. Papadakis S, Hermon Y, McEwan A. Standard Treatment Programme for Pregnant Women: A guide to behavioural support for smoking cessation during pregnancy and the post-partum period. In: National Centre for Smoking Cessation and Training (NCSCT); 2019: https://www.ncsct.co.uk/usr/pub/NCSCT%20Standard%20Treatment%20Programme%20for%20Pregnant%20Women.pdf. Accessed 07/10/2019.
29. Emery J, McDaid L, Coleman T, et al. Development and Content Validation of a Questionnaire for Measuring Beliefs About Using Nicotine Replacement Therapy for Smoking Cessation in Pregnancy. *Nicotine Tob Res.* 2023;25(7):1310-1318.
30. Heatherton TF, Kozlowski LT, Frecker RC, Rickert W, Robinson J. Measuring the heaviness of smoking: using self-reported time to the first cigarette of the day and

For peer review only

number of cigarettes smoked per day. *British Journal of Addiction.* 1989;84(7):791-799.
31. West R, Hajek P. Evaluation of the mood and physical symptoms scale (MPSS) to assess cigarette withdrawal. *Psychopharmacology (Berl).* 2004;177(1-2):195-199.
32. Huang Y, Emery J, Naughton F, et al. The development and acceptability testing of an app-based smart survey system to record smoking behaviour, use of nicotine replacement therapy (NRT) and e-cigarettes. *BMC Res Notes.* 2022;15(1):100.
33. Emery J, Huang Y, Naughton F, et al. Comparison of a Daily Smartphone App and Retrospective Questionnaire Measures of Adherence to Nicotine Replacement Therapy Among Pregnant Women: Observational Study. *JMIR Form Res.* 2023;7:e35045.
34. Coleman T, Cooper S, Thornton JG, et al. A randomized trial of nicotine-replacement therapy patches in pregnancy 8. *NEnglJMed.* 2012;366(9):808-818.
35. Cocks K, Torgerson DJ. Sample size calculations for pilot randomized trials: a confidence interval approach. *J Clin Epidemiol.* 2013;66(2):197-201.
36. Ussher M, Lewis S, Aveyard P, et al. Physical activity for smoking cessation in pregnancy: randomised controlled trial. *British Medical Journal.* 2015;350.
37. Tappin D, Bauld L, Purves D, et al. Financial incentives for smoking cessation in pregnancy: randomised controlled trial. *BMJ.* 2015;350.
38. Hartmann-Boyce J, Chepkin SC, Ye W, Bullen C, Lancaster T. Nicotine replacement therapy versus control for smoking cessation. *Cochrane Database of Systematic Reviews.* 2018(5).
39. West R, Hajek P, Stead L, et al. Outcome criteria in smoking cessation trials: proposal for a common standard. *Addiction.* 2005;100(3):299-303.
40. Jones M, Smith M, Lewis S, Parrott S, Coleman T. A dynamic, modifiable model for estimating cost-effectiveness of smoking cessation interventions in pregnancy: application to an RCT of self-help delivered by text message. *Addiction.* 2018;0(0).
41. Coleman T, Clark M, Welch C, et al. Effectiveness of offering tailored text message, self-help smoking cessation support to pregnant women who want information on stopping smoking: MiQuit3 randomised controlled trial and meta-analysis. *Addiction.* 2021.

Author's contributions

All authors contributed to study conception and design, participated in protocol writing and contributed to the preparation and drafting of the manuscript, a process which MC led.

Funding statement

This paper presents independent research funded by the National Institute for Health Research (NIHR) under its Programme Grants for Applied Research [Grant Reference Number RP-PG-0615-20003]. The views expressed are those of the authors and not necessarily those of the NIHR or the Department of Health and Social Care. Tim Coleman is an NIHR Senior Investigator.

Competing interest statement

FN is an unpaid member of the scientific committee for the Smoke Free app, a smoking cessation app unrelated to this project. All other authors have declared that they have no competing interests.

For peer review only - http://bmjopen.bmj.com/site/about/guidelines.xhtml

Roles and responsibilities

These membership lists are correct at the time of writing:

Roles of trial sponsor and funders

Name	Affiliation	Role
Alison Thorpe	University of Nottingham ('Sponsor')	Representative of the sponsor
Thomas Hutchinson	NIHR ('Funder')	Representative of the funder

Trial Team

Name	Affiliation	Role
Tim Coleman	University of Nottingham	Chief Investigator
Sue Cooper	University of Nottingham	Programme lead for Workstream 3.
Miranda Clark	University of Nottingham	Senior Trial Manager, trial management oversight
Kate Bowker	University of Nottingham	Trial Manager, day to day running of the trial
Lucy Phillips	University of Nottingham	Trial Manager, day to day running of the trial
Karen Daykin/ Nicki Stockdale	University of Nottingham	Trial Manager/Trial Coordinator, day to day running of the trial
Anne Dickinson	University of Nottingham	Researcher, trial intervention delivery and management of the delivery team
Daniel Robertson	University of Nottingham	Trial Coordinator, day to day running of the trial and participant follow up
Kasia Kowalewska	University of Nottingham	Trial Coordinator, day to day running of the trial and participant follow up
Anne Dickinson	University of Nottingham	Researcher, trial intervention delivery and management of the delivery team
Amy Morton	University of Nottingham	Trial administration, participant follow up
Eleanor Holmes	University of Nottingham	Trial administration, participant follow up
Katie Zhoya	University of Nottingham	Trial administration, participant follow up
Michelle Rawding	University of Nottingham	Trial administration, participant follow up
Daniel Simpkins		Senior Data Manager responsible for management of the database
Sarah Gardner	University of York	Database design and build
Matthew Bailey	University of York	Trial database set up and randomisation system build
Ross Thompson	University of Nottingham	Researcher, participant recruitment/consent
Lisa McDaid	University of East Anglia	Researcher, development of the intervention, participant recruitment/consent
Jo Emery	University of East Anglia	Researcher, development of the intervention, participant recruitment/consent
Felix Naughton	University of East Anglia	Programme lead for Workstream 1 and 2 of the NREADY programme

Trial Management Group

Name	Affiliation	Role
Tim Coleman	University of Nottingham	Chief Investigator
Sue Cooper	University of Nottingham	Programme manager
Miranda Clark	University of Nottingham	Senior Trial Manager
Kate Bowker	University of Nottingham	Trial Manager, day to day running of the trial
Lucy Phillips	University of Nottingham	Trial Manager, day to day running of the trial
Karen Daykin/ Nicki Stockdale	University of Nottingham	Trial Manager/Trial Coordinator, day to day running of the trial

Anne Dickinson	University of Nottingham	Researcher, trial intervention delivery and management of the delivery team
Ross Thomson	University of Nottingham	Researcher, participant recruitment/consent
Catherine Hewitt	University of York	Lead Trial statistician
Charlie Welch	University of York	Trial statistician
Gill Parkinson	University of York	Trial statistician
David Torgeson	University of York	Director of the York Trials Unit
Michael Ussher	St Georges, University of London	Population Health Science, and Social Marketing and Health
Sarah Lewis	University of Nottingham	Independent statistician

Trial Steering Committee

**Name**	**Affiliation**	**Role**
Peter Hajek	Queen Mary University London	Independent Chair
Martyn Willmore	Public Health England	Independent member
Jo Locker	Public Health England	Independent member
Donna Wilkes	PPI	Independent PPI Representative
Nikki Totton	University of Sheffield	Medical Independent statistician
Alison Thorpe	University of Nottingham ('Sponsor')	Observer (sponsor)
Thomas Hutchinson	NIHR	Observer (funder)
Catherine Hewitt	Lead Trial statistician (York Trials Unit)	Observer
Charlie Welch	Trial statistician (York Trials Unit)	Observer
Gill Parkinson	Trial statistician (York Trials Unit)	Observer
Tim Coleman	University of Nottingham	Non-independent member
Sue Cooper	University of Nottingham	Observer
Miranda Clark	University of Nottingham	Observer
Trial Manager	University of Nottingham	Observer
Felix Naughton	University of East Anglia	Observer

For peer review only

Participant Information Sheet
(Version 2.1 Date 12.09.22)

IRAS Project ID: 287771

Title of Study: **Smoking, Nicotine and Pregnancy 2 Trial (SNAP 2)**

Name of Chief Investigator: Tim Coleman

We would like to invite you to take part in our research study. Before you decide we would like you to understand why the research is being done and what it would involve for you. Please read the information below carefully. One of our team will go through the information sheet with you before you decide to take part and answer any questions you have. Talk to family, friends, or others about the study if you wish. Please ask us if there is anything that is not clear.

What is the purpose of the study?

- We want to improve the support that pregnant women receive to help them stop smoking.
- Pregnant women can use Nicotine Replacement Therapy (NRT) to help them stop smoking and the NHS prescribes this to them for free.
- However, pregnant women often do not use NRT in the best possible way and this can make it less effective than it could be.
- Therefore, in this study, we are testing a package of support which we hope will help pregnant women make better use of NRT so, it will have a better chance of helping them to stop smoking.

Why have I been invited?

We are inviting you to take part because you have told us that you are less than 25 weeks pregnant, smoke and are interested in getting help to quit, like NRT.

Do I have to take part?

It is up to you to decide whether or not to take part. If you do take part you will be given this information sheet to keep and will be asked to complete a consent form on paper, online or by telephone. If you join the study, you will be free to withdraw at any time without giving a reason. This would not affect your legal rights.

What will happen to me if I take part?

A computer will randomly place you into one of two groups with an equal chance of being in either.

Group One will receive support to stop smoking, which is the same as usual NHS support. You will be offered NRT as a patch, short acting NRT such as lozenges, inhalator or mouth spray or both together ('dual NRT'). You will receive up to six support sessions with a stop smoking practitioner (SSP). The first session will take place just before the day on which you stop smoking (quit date) and will last approximately *30 minutes*. Further consultations will be offered on or around *Day 3, Day 7, Day 14, Day 21, and Day 28* after your quit date. These will take place by telephone or video call and will last approximately *15 minutes* each.

Group Two will receive the same usual NHS stop smoking support that women in Group One receive, plus an intervention to help them make better use of NRT. This includes special support from a stop smoking practitioner, a leaflet, text messages, and a website. The first session will be just before the quit date and will last no longer than *45 minutes* and follow up consultations will last approximately *15 minutes* each and take place by telephone or video call.

For peer review only - http://bmjopen.bmj.com/site/about/guidelines.xhtml

Having two groups is a very important because it allows us to compare them and to learn about any benefits or disadvantages of the support we are testing. Joining the study will not affect your usual care and, should you decide not to participate, you will be offered the usual NHS support for stopping smoking which is available to you locally.

What would we expect from everyone taking part?

We will contact you by telephone, videocall, email, text or post. For some of the research information, we will send you a link by email or text asking you to complete a short questionnaire online:

- **When you first join the study.**

  We will ask questions about smoking and NRT. This should take no longer than *10 minutes*.

  We may ask you to provide a breath sample to measure your smoking. If you are selected to provide a breath test, we may send you a carbon monoxide meter and we will help you to set up an app on your mobile phone which helps you to record a carbon monoxide reading by blowing into this . Providing breath samples should take no longer than *5 minutes*.

  We will help you to download the NicUse app to your mobile phone. For each of the 28 days after your quit date, we will ask you to tell the app about your NRT use and any smoking and / or e-cigarette use. If you do not answer app questions for 2 days in a row, we will send a text message reminder.

- **Day 28 after your quit date**. We will ask about smoking and NRT and how you have got on providing a breath sample and using the app. If you are in Group 2, we will ask you some additional questions about your experiences of using the website, leaflet and receiving text messages; this should take no longer than *10 minutes*.

- **Towards the end of your pregnancy.** At around 36 weeks, we will ask you some questions about your smoking and NRT use. We may also ask you to provide saliva and breath samples. This should take no longer than *10 minutes*.

We will liaise with the hospital you are booked to deliver your baby to check how your pregnancy is progressing at approximately 34 weeks into your pregnancy, and again at a later date to find out details about the birth of your baby and your smoking status around delivery if required. We may contact you to ask you for information about the birth of your baby and your smoking status around delivery.

We will ask your permission to audio record some of your consultations with the stop smoking practitioner; this will help us monitor the quality of the support that we provide. This is optional. You will be able to take part in the study without agreeing to being recorded but, it would be helpful to the study if you were to agree. Audio recordings of consultations will be kept confidential. Only members of the research team will have access to these.

For peer review only - http://bmjopen.bmj.com/site/about/guidelines.xhtml

Below is a summary of what we would expect from you if you decided to take part in this study.

Research collection and stop smoking practitioner schedule

Baseline contact with advisor, research data collection and breath sample. Agree quit date and NRT.		Day 1: Quit Date APP	Day 2 APP	Day 3: Advisor contact APP	Day 4 APP
Day 5 APP	Day 6 APP	Day 7: Advisor contact APP SMS	Day 8 APP	Day 9 APP	Day 10 APP
Day 11 APP	Day 12 APP	Day 13 APP	Day 14: Advisor contact APP	Day 15 APP	Day 16 APP
Day 17 APP	Day 18 APP	Day 19 APP	Day 20 APP	Day 21: Advisor contact APP	Day 22 APP
Day 23 APP	Day 24 APP	Day 25 APP	Day 26 APP	Day 27 APP	Day 28: Final advisor contact, research data collection. APP

Support we give to you	Advisor telephone support	28 Day Supply of Nicotine Replacement Therapy (NRT)
What we ask from you	Saliva sample	Breath sample; APP Use a mobile phone app; Research data collection
36 weeks we will collect & possibly a further and		

Expenses

All texts we send you are free, but texts you send to us will be the same as texting from a UK mobile number. Please check with your mobile phone provider about text messaging charges.

What are the possible disadvantages and risks of taking part?

We do not foresee there being any risks from taking part in this study. However, we appreciate that taking part will use your time and may therefore be inconvenient. Also, if you are likely to be upset by receiving some basic information about the risks of smoking in pregnancy then it is best not to take part.

What are the possible benefits of taking part?

We cannot promise the study will help you, but all participants will receive support to stop smoking based on the best NHS standards of practice. The information you provide to us during the study will be invaluable in helping us devise ways of supporting women like you who want to stop smoking during their pregnancy.

What happens when the research study stops?

Once your involvement in the study ends you will continue to receive routine stop smoking support available to NHS patients in the locality, unless you choose not to. We can assist you with this. If you are interested in reading the findings from this study, you can agree for us to keep your contact details after the end of the study, so that we can share the overall results with you once these are available.

For peer review only - http://bmjopen.bmj.com/site/about/guidelines.xhtml

What if there is a problem?

If you have a concern about any aspect of the study, you should ask to speak with the study team in Nottingham who will do their best to answer your questions. If you remain unhappy and wish to complain formally, you can do this through the NHS Complaints Procedure. For advice on making a complaint, contact your local Patient Advice and Liaison Service (PALS) at your local hospital.

PALS offers confidential advice, support and information on health-related matters and can provide patients with more information about the complaints procedure and the Independent Complaints Advocacy Service (ICAS).

Will my taking part in the study be kept confidential?

We will follow ethical and legal practice and all information about you will be handled in confidence.
If you join the study, we will use information collected from you and your medical notes during the course of the research. All data will be kept **strictly confidential**, stored in a secure and locked office, and on a password protected database at the University of Nottingham or with trial colleagues at the University of Cambridge. Research data shared with individuals from other Universities who are working within our research team will not have access to identifiable data. Any information shared will use a unique personalised participant study number. Under UK Data Protection laws the University is the Data Controller (legally responsible for the data security) and the Chief Investigator of this study (named at the start of this document) is the Data Custodian (manages access to the data). The University of Nottingham is the data controller for the study. This means we are responsible for looking after your information and using it properly. Your rights to access, change or move your information are limited, as we need to manage your information in specific ways to comply with certain laws and for the research to be reliable and accurate. To safeguard your rights we will use the minimum personally-identifiable information possible.

<u>You can find out more about how we use your information and to read our privacy notice at:</u>
https://www.nottingham.ac.uk/utilities/privacy.aspx.

The data collected for the study will be looked at and stored by authorised persons from the research team. They may also be looked at by authorised people from regulatory organisations to check that the study is being carried out correctly. All will have a duty of confidentiality to you as a research participant and we will do our best to meet this duty. Audio recordings will be anonymised and will be accessed by members of the research team. Only members of the research team will have access to any audio recordings where you could be identified. Anonymised transcripts and personal details will be stored separately on a secure network.

If you consent, your contact information will be kept by the University of Nottingham for up to 3 years after the end of the study so that we are able to contact you about the findings of the study and possible follow-up studies. This information will be kept separately from the research data collected and only those who need to will have access to it. All research data will be kept securely for 7 years or longer if required. After this time your data will be disposed of securely. During this time all precautions will be taken by all those involved to maintain your confidentiality, only members of the research team given permission by the data custodian will have access to your personal data.

In order for you to receive the text messaging service, your mobile phone number will be shared with a text carrier called FastSMS and/or Esendex, after the study is completed your confidential information will permanently deleted from these carriers. Their full information security statement can be found here: https://fastsms.co.uk/downloads/fastsms-privacy-policy.pdf and https://www.esendex.co.uk/knowledge-hub/faqs/.

In accordance with the University of Nottingham's, the Government's and our funders' policies we may share our research data with researchers in other Universities and organisations, including those in other countries, for research in health and social care. Sharing research data is important to allow peer scrutiny, re-use (and

For peer review only - http://bmjopen.bmj.com/site/about/guidelines.xhtml

therefore avoiding duplication of research) and to understand the bigger picture in particular areas of research. Data sharing in this way is usually anonymised (so that you could not be identified) but if we need to share identifiable information we will seek your consent for this and ensure it is secure. You will be made aware then if the data is to be shared with countries whose data protection laws differ to those of the UK and how we will protect your confidentiality.

Although what you say in the consultations with your stop smoking practitioner is confidential, should you disclose anything to us which we feel puts you or anyone else at any risk, we may feel it necessary to report this to the appropriate persons.

What will happen if I don't want to carry on with the study?

Your participation is voluntary, and you are free to withdraw at any time, without giving any reason, and without your legal rights being affected. If you want to withdraw from the study, you can do so at any time by texting 07537404542, calling us on 0115 7486681, or by emailing snap2study@nottingham.ac.uk

If you withdraw from the study, we will no longer collect any information about you or from you but we will keep the information about you that we have already obtained. This information may have already been used in some analyses and may still be used in the final study analyses. To safeguard your rights, we will use the minimum personally identifiable information possible.

Involvement of the General Practitioner/Family doctor (GP) and hospital

We tell your GP and the hospital where you plan to deliver your baby, that you are taking part in the study and what this involves (with your consent). We will ask your GP if there is any reason why you might not be suitable to take part in this study.

What will happen to any samples I give?

Only members of the research team, relevant regulatory authorities and the University-approved laboratory who test the saliva will have access to the results of your samples. The saliva samples will be tested for the amount of cotinine and / or anabasine in them. Cotinine is a chemical that is produced when nicotine (from cigarettes) is broken down by the body, present in both NRT and tobacco smoke and anabasine is present in tobacco smoke. All samples will be stored in a monitored freezer at the University-approved laboratory that will carry out their testing.

If the saliva sample you provide us is taken by a researcher then they will post this sample to a University-approved laboratory to be tested on the day it was taken from you. We may ask you to post your samples directly to the University-approved laboratory, if samples are taken by yourself at home (using a pre-paid, stamped addressed envelope we will provide you). The sample will have a study number, initials, whether it is your first or subsequent sample, and date of sample, for identification so only the research team will be able to link your sample to you. Once the laboratory has analysed your sample and we have checked the results, the sample will be destroyed in accordance with the Human Tissue Act 2004.

What will happen to the results of the research study?

The results of the study may be presented to other researchers, at conferences and through publication in scientific and medical journals. No names will be used in the results and individuals will not be identifiable in any written reports or presentations. It is also intended that the findings will be used to design new techniques that stop smoking practitioners can use to support women to stop smoking during their pregnancy.

Who is organising and funding the research?

This research is being organised by the University of Nottingham and is being funded the National Institute for Health Research (NIHR), Programmes for Applied Health Research.

For peer review only - http://bmjopen.bmj.com/site/about/guidelines.xhtml

For peer review only

Who has reviewed the study?

All research in the NHS is looked at by an independent group of people, called a Research Ethics Committee, to protect your interests. This study has been reviewed and given favourable opinion by London Bloomsbury Research Ethics Committee.

Further information and contact details

Chief investigator: **Professor Tim Coleman**
University of Nottingham
Nottingham
NG7 2RD
Phone: 0115 8230204
Email: tim.coleman@nottingham.ac.uk

Senior Trial Manager: **Miranda Clark**
University of Nottingham
Nottingham
NG7 2RD
Email: miranda.clark@nottingham.ac.uk
Telephone: 0115 7486681

General trial: **Email:** snap2study@nottingham.ac.uk
Telephone: 0115 7486681

For peer review only - http://bmjopen.bmj.com/site/about/guidelines.xhtml

University of Nottingham UK | CHINA | MALAYSIA
UEA University of East Anglia
University of York
FUNDED BY NIHR | National Institute for Health Research

CONSENT FORM

(Final Version 2.1 12.09.22)

Title of Study: Smoking, Nicotine and Pregnancy 2 Trial (SNAP 2)

Chief Investigator: Professor Tim Coleman
Principal Investigator:
Participant Name:
Participant Number:

Site Number:
REC ID: 21/LO/0123
IRAS ID: 287771

Please initial box

1. I confirm that I have read and understand the Participant Information Leaflet version number 2.1 12.09.22 for the above study and have had the opportunity to ask questions. ☐

2. I understand that my participation is voluntary and that I am free to withdraw at any time, without giving any reason, and without my medical care or legal rights being affected. I understand that should I withdraw then the information collected so far cannot be erased and that this information may still be used in the project analysis. ☐

3. I understand that relevant sections of my medical notes and data collected in the study may be looked at by authorised individuals from the University of Nottingham, the research group (University of East Anglia and University of York) and regulatory authorities where it is relevant to my taking part in this study. I give permission for these individuals, where it is relevant, to collect, store, analyse and publish information obtained from my participation in this study. I understand that my personal details will be kept confidential. ☐

4. I understand and agree that breath samples and/or saliva samples will be requested to measure my carbon monoxide and nicotine levels. ☐

5. I agree to being followed-up by the research team during the study by telephone/video call, text, email, post, or face-to-face appointments. ☐

6. I agree to my GP and hospital where I will deliver my baby being informed of my participation in this study, and to my GP being asked to provide information if there are any reasons that I should not take part. ☐

7. I agree to my mobile phone number being used in this study and consent to it being passed to the University of Cambridge and their nominated text carrier (FastSMS), and/or a different text carrier (Esendex), so that I can receive study related text messages. I understand that my mobile number will only be used for this study. ☐

8. I understand that my anonymised data collected in the study may be used to support other research in the future and may be shared with other researchers. ☐

9. **(Not essential to study participation)** I agree to have my contact details kept after the end of the study (for a maximum of 3 years) so that I can be contacted about the findings of the study and informed of follow-up studies. **Yes** ☐ **No** ☐

10. **(Not essential to study participation)** I agree that the stop smoking consultation I receive can be audio recorded and that anonymous quotes from the consultation may be used in study reports. **Yes** ☐ **No** ☐

11. I agree to take part in the above study. ☐

Name of Participant	Date	Signature
Name of Person Taking Consent	Date	Signature

3 copies: 1 for participant, 1 for the project notes and 1 for the medical notes

For peer review only - http://bmjopen.bmj.com/site/about/guidelines.xhtml

Reporting checklist for protocol of a clinical trial.

Based on the SPIRIT guidelines.

Instructions to authors

Complete this checklist by entering the page numbers from your manuscript where readers will find each of the items listed below.

Your article may not currently address all the items on the checklist. Please modify your text to include the missing information. If you are certain that an item does not apply, please write "n/a" and provide a short explanation.

Upload your completed checklist as an extra file when you submit to a journal.

In your methods section, say that you used the SPIRITreporting guidelines, and cite them as:

Chan A-W, Tetzlaff JM, Gøtzsche PC, Altman DG, Mann H, Berlin J, Dickersin K, Hróbjartsson A, Schulz KF, Parulekar WR, Krleža-Jerić K, Laupacis A, Moher D. SPIRIT 2013 Explanation and Elaboration: Guidance for protocols of clinical trials. BMJ. 2013;346:e7586

For peer review only

		Reporting Item	Page Number
**Administrative information**			
Title	#1	Descriptive title identifying the study design, population, interventions, and, if applicable, trial acronym	1
Trial registration	#2a	Trial identifier and registry name. If not yet registered,	2

		name of intended registry	
Trial registration: data set	#2b	All items from the World Health Organization Trial Registration Data Set	na

Fully anonymized trial data will be available upon request from the York Trials Unit

Protocol version	#3	Date and version identifier	2
Funding	#4	Sources and types of financial, material, and other support	21
Roles and responsibilities: contributorship	#5a	Names, affiliations, and roles of protocol contributors	1,20
Roles and responsibilities: sponsor contact information	#5b	Name and contact information for the trial sponsor	21
Roles and responsibilities: sponsor and funder	#5c	Role of study sponsor and funders, if any, in study design; collection, management, analysis, and interpretation of data; writing of the report; and the decision to submit the report for publication, including whether they will have ultimate authority over any of these activities	Suppl. materials
Roles and responsibilities: committees	#5d	Composition, roles, and responsibilities of the coordinating centre, steering committee, endpoint adjudication committee, data management team, and	Suppl. materials

For peer review only

		other individuals or groups overseeing the trial, if applicable (see Item 21a for data monitoring committee)	
**Introduction**			
Background and rationale	#6a	Description of research question and justification for undertaking the trial, including summary of relevant studies (published and unpublished) examining benefits and harms for each intervention	4-5
Background and rationale: choice of comparators	#6b	Explanation for choice of comparators	5
Objectives	#7	Specific objectives or hypotheses	4-5
Trial design	#8	Description of trial design including type of trial (eg, parallel group, crossover, factorial, single group), allocation ratio, and framework (eg, superiority, equivalence, non-inferiority, exploratory)	5
**Methods: Participants, interventions, and outcomes**			
Study setting	#9	Description of study settings (eg, community clinic, academic hospital) and list of countries where data will be collected. Reference to where list of study sites can be obtained	6-7
Eligibility criteria	#10	Inclusion and exclusion criteria for participants. If	6

For peer review only

		applicable, eligibility criteria for study centres and individuals who will perform the interventions (eg, surgeons, psychotherapists)	
Interventions: description	#11a	Interventions for each group with sufficient detail to allow replication, including how and when they will be administered	7-8
Interventions: modifications	#11b	Criteria for discontinuing or modifying allocated interventions for a given trial participant (eg, drug dose change in response to harms, participant request, or improving / worsening disease)	8
Interventions: adherance	#11c	Strategies to improve adherence to intervention protocols, and any procedures for monitoring adherence (eg, drug tablet return; laboratory tests)	8
Interventions: concomitant care	#11d	Relevant concomitant care and interventions that are permitted or prohibited during the trial	7-8
Outcomes	#12	Primary, secondary, and other outcomes, including the specific measurement variable (eg, systolic blood pressure), analysis metric (eg, change from baseline, final value, time to event), method of aggregation (eg, median, proportion), and time point for each outcome. Explanation of the clinical relevance of chosen efficacy and harm outcomes is strongly recommended	13
Participant timeline	#13	Time schedule of enrolment, interventions (including any run-ins and washouts), assessments, and visits for	10-12

For peer review only

		participants. A schematic diagram is highly recommended (see Figure)	
Sample size	#14	Estimated number of participants needed to achieve study objectives and how it was determined, including clinical and statistical assumptions supporting any sample size calculations	13-14
Recruitment	#15	Strategies for achieving adequate participant enrolment to reach target sample size	6-7
**Methods: Assignment of interventions (for controlled trials)**			
Allocation: sequence generation	#16a	Method of generating the allocation sequence (eg, computer-generated random numbers), and list of any factors for stratification. To reduce predictability of a random sequence, details of any planned restriction (eg, blocking) should be provided in a separate document that is unavailable to those who enrol participants or assign interventions	6
Allocation concealment mechanism	#16b	Mechanism of implementing the allocation sequence (eg, central telephone; sequentially numbered, opaque, sealed envelopes), describing any steps to conceal the sequence until interventions are assigned	7
Allocation: implementation	#16c	Who will generate the allocation sequence, who will enrol participants, and who will assign participants to	7

For peer review only

		interventions	
Blinding (masking)	#17a	Who will be blinded after assignment to interventions (eg, trial participants, care providers, outcome assessors, data analysts), and how	7
Blinding (masking): emergency unblinding	#17b	If blinded, circumstances under which unblinding is permissible, and procedure for revealing a participant's allocated intervention during the trial	7
**Methods: Data collection, management, and analysis**			
Data collection plan	#18a	Plans for assessment and collection of outcome, baseline, and other trial data, including any related processes to promote data quality (eg, duplicate measurements, training of assessors) and a description of study instruments (eg, questionnaires, laboratory tests) along with their reliability and validity, if known. Reference to where data collection forms can be found, if not in the protocol	9, 11-12
Data collection plan: retention	#18b	Plans to promote participant retention and complete follow-up, including list of any outcome data to be collected for participants who discontinue or deviate from intervention protocols	12
Data management	#19	Plans for data entry, coding, security, and storage,	12-13

For peer review only

		including any related processes to promote data quality (eg, double data entry; range checks for data values). Reference to where details of data management procedures can be found, if not in the protocol	
Statistics: outcomes	#20a	Statistical methods for analysing primary and secondary outcomes. Reference to where other details of the statistical analysis plan can be found, if not in the protocol	14
Statistics: additional analyses	#20b	Methods for any additional analyses (eg, subgroup and adjusted analyses)	14
Statistics: analysis population and missing data	#20c	Definition of analysis population relating to protocol non-adherence (eg, as randomised analysis), and any statistical methods to handle missing data (eg, multiple imputation)	14

Methods: Monitoring

Data monitoring: formal committee	#21a	Composition of data monitoring committee (DMC); summary of its role and reporting structure; statement of whether it is independent from the sponsor and competing interests; and reference to where further details about its charter can be found, if not in the protocol. Alternatively, an explanation of why a DMC is not needed	n/a

As the trial tests the ability of a behavioural intervention to improve women's use of a standard treatment for smoking cessation, we do not consider that a Data Monitoring Committee (DMC) is required. This trial is not categorised as a CTIMP, so formal adverse event monitoring is not

For peer review only

required.

Data monitoring: interim analysis	#21b	Description of any interim analyses and stopping guidelines, including who will have access to these interim results and make the final decision to terminate the trial	n/a

As this is an evaluation of a behavioural intervention, we do not anticipate there to be a need to discontinue the trial due to adverse outcomes or for any other reasons. Adverse pregnancy outcomes, which are thought to be associated with smoking, will be examined to assess any changes in their prevalence caused by the intervention rather than as an assessment of the intervention itself. In addition, there will be no formal comparative monitoring of accumulating outcome data, and no comparison of any emerging signal with benefit or futility stopping boundaries/rules.

Harms	#22	Plans for collecting, assessing, reporting, and managing solicited and spontaneously reported adverse events and other unintended effects of trial interventions or trial conduct	15
Auditing	#23	Frequency and procedures for auditing trial conduct, if any, and whether the process will be independent from investigators and the sponsor	n/a

There are no plans to audit trial conduct beyond the scope of the data management plan

Ethics and dissemination

Research ethics approval	#24	Plans for seeking research ethics committee / institutional review board (REC / IRB) approval	15

For peer review only

Protocol amendments	#25	Plans for communicating important protocol modifications (eg, changes to eligibility criteria, outcomes, analyses) to relevant parties (eg, investigators, REC / IRBs, trial participants, trial registries, journals, regulators)	15
Consent or assent	#26a	Who will obtain informed consent or assent from potential trial participants or authorised surrogates, and how (see Item 32)	6-7
Consent or assent: ancillary studies	#26b	Additional consent provisions for collection and use of participant data and biological specimens in ancillary studies, if applicable	6-7
Confidentiality	#27	How personal information about potential and enrolled participants will be collected, shared, and maintained in order to protect confidentiality before, during, and after the trial	12
Declaration of interests	#28	Financial and other competing interests for principal investigators for the overall trial and each study site	20
Data access	#29	Statement of who will have access to the final trial dataset, and disclosure of contractual agreements that limit such access for investigators	15
Ancillary and post trial care	#30	Provisions, if any, for ancillary and post-trial care, and for compensation to those who suffer harm from trial participation	8,15
Dissemination policy:	#31a	Plans for investigators and sponsor to communicate trial	15

For peer review only

trial results		results to participants, healthcare professionals, the public, and other relevant groups (eg, via publication, reporting in results databases, or other data sharing arrangements), including any publication restrictions	
Dissemination policy: authorship	#31b	Authorship eligibility guidelines and any intended use of professional writers	15
Dissemination policy: reproducible research	#31c	Plans, if any, for granting public access to the full protocol, participant-level dataset, and statistical code	15

Appendices

Informed consent materials	#32	Model consent form and other related documentation given to participants and authorised surrogates	Suppl. materials
Biological specimens	#33	Plans for collection, laboratory evaluation, and storage of biological specimens for genetic or molecular analysis in the current trial and for future use in ancillary studies, if applicable	13

None The SPIRIT Explanation and Elaboration paper is distributed under the terms of the Creative Commons Attribution License CC-BY-NC. This checklist can be completed online using https://www.goodreports.org/, a tool made by the EQUATOR Network in collaboration with Penelope.ai

For peer review only

BMJ Open

Smoking, nicotine and pregnancy 2 (SNAP2) trial: protocol for a randomised controlled trial of an intervention to improve adherence to nicotine replacement therapy in pregnancy

Journal:	*BMJ Open*
Manuscript ID	bmjopen-2024-087175.R1
Article Type:	Protocol
Date Submitted by the Author:	23-Apr-2024

Complete List of Authors:	Clark, Miranda; University of Nottingham, Centre for Academic Primary Care, School of Medicine<br>Cooper, Sue; University of Nottingham, Centre for Academic Primary Care, School of Medicine<br>Naughton, Felix; University of East Anglia School of Health Sciences<br>Ussher, Michael; St George's University of London, Population Health Research Institute; University of Stirling, Institute of Social Marketing and Health<br>Emery, Joanne; University of East Anglia School of Health Sciences<br>McDaid, Lisa; University of East Anglia, School of Health Sciences<br>Thomson, Ross; University of Nottingham School of Medicine, Centre for Academic Primary Care<br>Phillips, Lucy; University of Nottingham School of Medicine, Centre for Academic Primary Care<br>Bauld, Linda; The University of Edinburgh, Usher Institute<br>Aveyard, Paul; University of Oxford Division of Public Health and Primary Health Care, Primary Care Health Sciences<br>Torgerson, David; University of York, York Trials Unit, Department of Health Sciences<br>Berlin, Ivan; Pitié Salpêtrière Hospital-Sorbonne Université, Department of Medical Pharmacology<br>Lewis, Sarah; University of Nottingham, Faculty of Medicine and Health Sciences<br>Parrott, Steve; University of York, Department of Health Sciences<br>Hewitt, Catherine; University of York, York Trials Unit, Department of Health Sciences<br>Welch, Charlie; University of York, York Trials Unit, Department of Health Sciences<br>Parkinson, Gill; University of York, York Trials Unit, Department of Health Sciences<br>Dickinson, Anne; University of Nottingham School of Medicine, Centre for Academic Primary Care<br>Sutton, Stephen; University of Cambridge, Department of Public Health and Primary Care<br>Brimicombe, James; University of Cambridge, Primary Care Unit, Department of Public Health and Primary Care<br>Bowker, Katharine; University of Nottingham School of Medicine, Centre for Academic Primary Care

	McEwen, Andrew; University College London, Department of Behavioural Science and Health<br>Vedhara, Kavita; University of Nottingham School of Medicine, Centre for Academic Primary Care; Cardiff University, School of Psychology<br>Coleman, Tim; University of Nottingham, Centre for Academic Primary Care, School of Medicine
**Primary Subject Heading**:	Smoking and tobacco
Secondary Subject Heading:	Addiction, Public health, Obstetrics and gynaecology
Keywords:	PUBLIC HEALTH, Smoking Reduction, Maternal medicine < OBSTETRICS, RESPIRATORY MEDICINE (see Thoracic Medicine)

SCHOLARONE™ Manuscripts

Smoking, nicotine and pregnancy 2 (SNAP2) trial: protocol for a randomised controlled trial of an intervention to improve adherence to nicotine replacement therapy in pregnancy

Miranda Clark [1], Sue Cooper [1], Felix Naughton [2], Michael Ussher [3], Joanne Emery [2], Lisa McDaid [2], Ross Thomson [1], Lucy Phillips [1], Linda Bauld [4], Paul Aveyard [5], David Torgerson [6], Ivan Berlin [7], Sarah Lewis [8], Steve Parrott [9], Catherine Hewitt [6], Charlie Welch [6], Gill Parkinson [6], Anne Dickinson [1], Stephen Sutton [10], James Brimicombe [10], Kate Bowker [11], Andy McEwen [12], Kavita Vedhara [13], Tim Coleman [1].

1. Centre for Academic Primary Care, School of Medicine, University of Nottingham, Nottingham, UK
2. Behavioural and Implementation Science Group, School of Health Sciences, University of East Anglia, Norwich, UK
3. Division of Population Health Sciences and Education, St. George's, University of London and Institute of Social Marketing and Health, University of Stirling, Stirling, UK
4. Usher Institute and SPECTRUM Consortium, University of Edinburgh, Edinburgh, UK
5. Nuffield Department of Primary Care Health Sciences, University of Oxford, Oxford, UK
6. York Trials Unit, Department of Health Sciences, University of York, York, UK
7. Department of Medical Pharmacology, Pitié Salpêtrière Hospital-Sorbonne Université, Paris, France
8. Faculty of Medicine and Health Sciences, University of Nottingham, Nottingham, UK
9. Institute of Public Health, University of Cambridge, Cambridge, UK
10. Behavioural Science Group, University of Cambridge, Cambridge, UK
11. Nottingham Clinical Trials Unit, School of Medicine, University of Nottingham, Nottingham, UK
12. Department of Behavioural Science and Health, University College London, London, UK
13. School of Psychology, Cardiff University, Cardiff, UK

Correspondence to:
Miranda Clark
miranda.clark@nottingham.ac.uk

For peer review only

Abstract

Introduction: Smoking in pregnancy is harmful for unborn babies, infants, and women. Nicotine replacement therapy (NRT) is offered as usual stop smoking support in the UK. However, this is often used in insufficient doses, intermittently, or for too short a time to be effective. This randomised controlled trial (RCT) explores whether a bespoke intervention, delivered in pregnancy, improves adherence to NRT and is effective and cost effective for promoting smoking cessation.

Methods and analysis: A two-arm parallel-group RCT for pregnant women aged ≥ 16 years and who smoke ≥1 daily cigarette (pre-pregnancy smoked ≥5), and who agree to use NRT in a quit attempt. Recruitment is from antenatal care settings and via social media adverts. Participants are randomised using blocked randomisation with varying block sizes, stratified by gestational age (<14 weeks or ≥14 weeks) to receive: i) usual care stop smoking support (UC) or ii) UC plus an intervention to increase adherence to NRT, called "Baby, Me and NRT", comprising adherence counselling, automated tailored text messages, a leaflet and website. The primary outcome is biochemically validated smoking abstinence at or around childbirth, measured from 36 weeks gestation. Secondary outcomes include NRT adherence; other smoking measures; and birth outcomes. Questionnaires collect follow up data which are augmented by medical records information. We anticipate quit rates of 10% and 16% in control and intervention groups respectively (RR = 1.6). By recruiting 1320 participants the trial should have 90% power (alpha = 5%) to detect this intervention effect. An economic analysis will use the Economics of Smoking in Pregnancy (ESIP) model to determine cost-effectiveness.

Ethics and dissemination: Ethics approval was granted by Bloomsbury NHS Research Ethics Committee (21/LO/0123). Written informed consent will be obtained from all participants. Findings will be disseminated to the public, funders, relevant practice / policy representatives, researchers, and participants.

Trial registration: ISRCTN16830506.

Protocol version: 5.0, 10 Oct 2023.

Strengths and limitations of this study

- This randomised controlled trial (RCT) is testing an intervention aimed at addressing women's concerns about and barriers to nicotine replacement therapy (NRT) adherence.
- The trial design is explanatory and pragmatic, and so will show whether changes in smoking are due to altered adherence to NRT.
- We report the design of the RCT according to the SPIRIT guidelines.
- Participants are not blind to the treatments and this could cause bias, which is limited by using biochemical verification of abstinence as the primary outcome.
- Obtaining data on smoking abstinence from pregnant people who smoke is difficult; using routine data may ameliorate this.

For peer review only - http://bmjopen.bmj.com/site/about/guidelines.xhtml

INTRODUCTION

Smoking in pregnancy is still a prevalent public health issue worldwide. For example in the UK, around 7.5% of UK women smoke at childbirth(1). Smoking in pregnancy is associated with negative outcomes for both women and babies, but is preventable, and women who stop smoking in pregnancy are less likely to have premature or low birth weight infants.(2) Compared with mothers who do not smoke, those who continue smoking in pregnancy have heightened risks of placental abruption, miscarriage, still birth and ectopic pregnancy.(3) Children born to parents who smoke are more likely to start smoking themselves(4) and tobacco smoking is a major risk factor for six of the eight leading causes of death worldwide.(5)

Pregnancy is probably a life event that most motivates people to try stopping smoking; around 50% of women who smoke stop during gestation; many others try but fail.(6) A key reason is that nicotine withdrawal symptoms and smoking urges experienced on stopping smoking are difficult to tolerate. Nicotine replacement therapy (NRT) provides nicotine without exposing users to toxins like tar, cyanide and carbon monoxide, and so safely helps ameliorate withdrawal and smoking urges. In the UK, National Institute for Health and Care Excellence (NICE) recommended NRT use in pregnancy since 2010(7, 8) and is now a central component of routine clinical practice there.(9, 10),(11) However, although NRT is effective in general(12), it appears to work less well in pregnancy(13), probably because pregnant women do not use it consistently, for long enough or in sufficient doses. In trials enrolling pregnant smokers, only 7% to 30% finished courses of NRT(13), and of pregnant smokers prescribed NRT by UK GPs, only 30% were supplied it for longer than 2 weeks(11) and such short NRT courses are ineffective. In contrast, non-pregnant smokers enrolled in smoking cessation trials report up to 94% adherence levels.(14)

It is very likely that for NRT to work in pregnancy, higher nicotine doses and dosing consistency are needed than are currently used. In pregnancy nicotine metabolism accelerates(15, 16) resulting in NRT generating lower blood nicotine concentrations. Additionally, research suggests that many pregnant women struggle to use NRT consistently or for sufficiently long, due to concerns about nicotine safety and a lack of belief in the need of NRT to quit smoking. These are influenced by erroneous lay beliefs which contribute to idiosyncratic NRT usage patterns and NRT not being used as advised (17, 18) Such erratic NRT use can be compounded by inaccurate advice on nicotine safety from friends, family, and even health professionals, exacerbating women's uncertainties about whether and how to use NRT.(19) Both stop smoking practitioners(20) and pregnant women(21) believe that by consistently countering such misinformation, improvements could be made in the number of successful quit attempts. Poor adherence to, and intermittent use of NRT, very likely reduces the chances of smoking cessation in pregnancy, limiting the health benefits that could accrue from optimal use of this treatment.

If better adherence to NRT is not more harmful to the foetus than smoking and helps more pregnancies become smokefree whilst avoiding smoking-related harms, encouraging adherence to NRT would clearly be ethical. There is no biological rationale for suspecting that NRT could be more harmful than smoking in pregnancy. Throughout the 2000s, based on the logical belief that smoking-related harms in pregnancy are unlikely to be due solely to nicotine, there has been expert consensus for using NRT to stop smoking in pregnancy.(22) NRT in pregnancy is not recommended for 'never smokers', but NRT used *instead* of

smoking is very likely to be safer. In the unlikely event of unexpected nicotine-attributable foetal harm(s) occurring, one would expect these to be vastly outweighed by benefits from smoking cessation following effective NRT use. A Cochrane review found no evidence that, for pregnancy outcomes, NRT harms either women or their babies, although analyses were generally underpowered to detect moderate sized effects(13) and observational studies are not sufficiently robust to add to these findings.(23) However, compared to smoking, NRT has an apparently protective effect on infant development; at 2 years old, infants in the largest RCT of NRT in pregnancy(24), born to women randomised to NRT rather than placebo, were more likely to have unimpaired development(25).

Rationale

In a National Institute for Health Research-funded programme, we developed '*Baby, Me & NRT*' (BMN), an intervention to improve adherence to NRT during pregnancy.(26) In cohort studies, we optimised and monitored impacts of BMN and this RCT explores whether BMN helps pregnant women stop smoking and increases adherence to NRT. In this paper we report the protocol of SNAP2 according to the SPIRIT guidelines.(27)

METHODS AND ANALYSIS

SNAP2 is a multi-centre, parallel group, individually randomised controlled trial of the 'Baby, Me & NRT' (BMN) intervention integrated with usual smoking cessation support during pregnancy versus usual smoking cessation support alone.

This RCT was originally envisaged solely as a 'proof of concept' study that aimed to detect whether BMN increased NRT adherence. If so, a separate RCT was planned to explore BMN effects on cessation. However, due to NHS service provision changes and the Covid pandemic, the funder accepted that, following demonstration of 'proof of concept' from a pilot phase, efficacy could be tested by recruiting sufficient participants to SNAP2. Below, we indicate which methodological features were used only in the pilot, and the sample size section explains the basis for progression from pilot to full trial.

Objectives

Primary objective

To determine whether, when added to usual NHS cessation support, the 'Baby, Me & NRT' intervention increases smoking abstinence during pregnancy, as measured in late pregnancy or at childbirth, with exhaled carbon monoxide and/or saliva samples used to validate self-reported abstinence.

Secondary objectives

In ***all*** participants, to compare between intervention and usual care groups:

i) Reported smoking abstinence at 28 days after a quit date

ii) Reported smoking abstinence at both 28 days after a quit date, and in late pregnancy / childbirth with and without validation in late pregnancy

iii) The number of days of NRT use in the first 28 days after a quit date.

iv) Mean daily nicotine dose in the first 7 days after a quit date ('intensity' of NRT use)

v) Adverse pregnancy outcome rates

For peer review only - http://bmjopen.bmj.com/site/about/guidelines.xhtml

In ***intervention group*** participants:
vi) To assess engagement with BMN intervention components.

Economic
vii) To investigate cost-effectiveness of the BMN intervention.

Pilot phase objectives
In ***all*** pilot phase participants, to compare between intervention and usual care groups:

viii) Urges to smoke and tobacco withdrawal symptoms at Day 7 after a quit date

ix) NRT concerns and necessity beliefs at Day 28.

In pilot phase ***intervention group*** participants and practitioners only:

x) To assess fidelity of BMN intervention delivery

Other objectives (pilot phase only)
To compare biochemically measured nicotine exposure before and after exposure to BMN intervention.

Participants and setting

Inclusion criteria
People are eligible if aged ≥ 16; pregnant and <25 weeks' gestation; they smoked ≥ 5 daily cigarettes before pregnancy (currently smoking ≥1 daily cigarette) and are referred for or receiving antenatal care. Participants must have sufficient understanding of English to give informed consent; agree to try stopping smoking with NRT within 14 days, to receive and send SMS text messages, and install the trial's data collection app on their smartphone.
.
Exclusion criteria
They are ineligible if already using NRT or are enrolled in a smoking cessation study, NHS stop smoking support or a cessation-orientated text message service, or they intend to continue using e-cigarettes or have contraindications to NRT.

Recruitment
Participants will be identified from:

i) National Health Service (NHS) clinical settings, by poster, direct contact from researchers (face-to-face or distanced) and online, with adverts in NHS digital spaces.
ii) Online, outside of NHS settings

NHS settings
These can be hospital antenatal care, general practice, community midwifery or stop smoking service settings. Researchers may identify potential participants from medical records, contacting them by letter, telephone, email or text before appointments, and including QR code / links to or paper copies of a Patient Information Sheet (PIS) (see supplementary files). They may also approach pregnant people attending clinics asking them to complete an eligibility screening questionnaire or give them a summary leaflet which contains links / QR codes leading to the PIS. Depending on the setting, researchers may

For peer review only - http://bmjopen.bmj.com/site/about/guidelines.xhtml

consent those who are interested and eligible, or they may pass contact details to the trial team (see supplementary files) to enable consent to be received by them.

Posters describing the trial will be displayed in clinical areas or in appropriate NHS digital spaces; these will include QR codes or links leading to the PIS, and to an online version of the screening questionnaire, following which eligible and interested people will be invited to leave contact details in a secure RedCap database hosted by the University of Nottingham. The trial team can then access contact details and contact potential participants directly.

Online, outside of NHS settings
Google or social media (e.g. Facebook, Instagram,) adverts will be targeted at those who smoke and are pregnant. Embedded links will lead to a study information webpage and to an eligibility questionnaire, and those who are potentially eligible will be asked to enter contact details, as above.

Interested, eligible potential participants will be given at least 24 hours to consider the PIS before discussion with a researcher and informed consent received. Discussion and documentation of consent could be by face-to-face (using 'wet ink') or 'distanced' using either an online form or by telephone (see supplementary files). If the online form is used, eligible potential participants will be sent this via a link and complete this during a telephone conversation with a researcher. Consent via telephone is similar but in this case the consent form will be generated by the researcher from the research database, following a strict protocol, then signed copies will be shared with the participant. For all consent methods, a letter will be sent to the participant's GP informing them of the enrolment.

Randomisation and blinding

After informed consent, participants' baseline data will be collected before randomising them to either study arm with the York Trials Unit's (YTU) web-based system. The randomisation schedule will be computer-generated, with pseudo-random code using random permuted blocks of randomly varying size and stratified by gestational age (<14 weeks or ≥14 weeks). Immediately afterward, the trial office receives email confirmation of treatment allocation. Participants and those delivering interventions will be aware of treatment allocations, but researchers who collect data will be blinded. To prevent BMN components being inadvertently delivered to the usual care group, two separate teams of stop smoking practitioners (SSPs) will be used to deliver smoking cessation support.

Interventions

Control
Usual Care for smoking cessation, following the National Centre for Smoking Cessation Training (NCSCT) standard treatment programme[28] and which comprises:

i) Help setting a quit date
ii) Up to 6 telephone or video call counselling sessions
iii) An offer of NRT as patch, short-acting NRT or both together ('dual NRT'). (9, 10)

Before Quit Date (QD): SSPs assess participants' suitability for NRT in terms of, cautions or contraindications, other prescription medications and health issues, and counsel participants on how best to use NRT as per the NCSCT guidelines. Guidelines advise the 'not a puff' rule where NRT should only be used when not smoking. If there is doubt about NRT safety, participants' GPs are consulted to assess their medical suitability to using NRT products. Participants are mailed a 14-day supply of their chosen NRT product(s) and are instructed to start this on the QD.

For peer review only - http://bmjopen.bmj.com/site/about/guidelines.xhtml

After Quit Date: Practitioners offer counselling appointments between the QD and Day 3 and on Days 7, 14, 21 and 28 after the QD. SSPs ask about withdrawal, use of NRT and experience of nicotine side effects, and advise on effective NRT use. At Day 14, participants still using NRT are offered a further 14-day supply.

NRT: Advice given to use dual therapy (one long acting and one short acting product) with dose titrated to the number of cigarettes smoked per day. For those who cannot tolerate patches, 2 short acting products can be substituted with advice on how to ensure round the clock coverage. Participants may choose from the following products supplied in their original packaging; all have UK licenses for use in pregnancy:

Patches: Daily Nicorette 16-hr (15mg or 25mg) or NiQuitin clear 24 hr (14mg or 21 mg).

Short acting NRT: Nicorette Inhalator 15mg (max 6 cartridges / day); Nicorette Cools Lozenges 2mg or 4 mg (max 8-12 lozenges / day); Nicorette QuickMist mouthspray 1mg/spray (Maximum: 2 sprays at a time; 4 sprays / hour; 64 sprays / day).

For both trial groups, support beyond the 28 days during which trial interventions are delivered, is provided by locally available UC NHS support. Participants were encouraged to attend all counselling sessions with reminder texts sent prior to appointments and up to three follow up scheduled texts if they did not attend.

Intervention

The 'Baby, Me & NRT' (BMN) intervention is offered alongside UC (described above) and integrated into an identical schedule. BMN is described in detail elsewhere(26) and comprises of tailored behavioural support designed to encourage adherence to NRT and increase quit rates during pregnancy. The main components are:

Counselling: Participants are asked to complete a short questionnaire to assess their concerns and necessity beliefs regarding use of NRT in pregnancy(29); the latter are views on how worthwhile NRT might be to participants. The number of counselling sessions mirror usual care, but the content addresses individual concerns and beliefs about the safety of nicotine and the efficacy of nicotine replacement products The first counselling session is on average 10 minutes longer than those delivered in usual care, is delivered by video call where possible and addresses individual concerns and beliefs about the safety of nicotine. To ensure advice is as personalised as possible, SSPs respond to key concerns and necessity beliefs recorded on questionnaires. Participants are advised, if needed, to use a patch and short-acting NRT preparation until childbirth, and during brief lapses to smoking (of up to 14 days), provided they still try to quit. To avoid morning cravings, 24-hour NRT patches may be left on overnight, and support is aimed at maintaining adherence to NRT. Follow up calls are mainly by telephone; these again focus on addressing concerns about nicotine, using sufficient NRT and on not stopping this during brief smoking lapses, in addition to the UC advice.

Leaflet and website: These reinforce key NRT adherence-enhancing messages using video animations and careful wording. Additionally, there are video clips from experts and/or written experiences with NRT from other pregnant women.

Text messages: For up to 30 days, we send personally tailored, automated texts, based on participants' smoking behaviours and NRT-related beliefs. These aim to support participants' abstinence; encourage using sufficient NRT to control withdrawal symptoms and cravings; counter intentional non-adherence to NRT (e.g. due to nicotine-related

For peer review only

concerns) and provide prompts or reminders to prevent non-intentional non-adherence to NRT (e.g., forgetting).

Staff delivering interventions
All SSPs delivering support are trained to the recognised NCSCT standard required for delivering UC stop smoking support in the UK NHS.[26] All intervention group participants are counselled by specially trained SSPs working within the research team, who deliver the Baby, Me and NRT, components integrated into usual care. Control group counselling is provided by either a separate group of research team SSPs or by NHS providers responsible for providing locally available Usual Care stop smoking support.

Data collection

Table 1 shows all participant data collection at time points outlined below and indicates how intervention delivery fits with this. Figure 1 is a study flow diagram. We indicate which measures will be used for research purposes in the pilot only.

Table 1. Schedule of data collection and intervention delivery time points

	**Time point**								
**Data collected**	**Pre-baseline (consent)**	**Baseline[1]**	**Pre-Quit Date**	**Day 1-3**	**Day 7**	**Day 14**	**Day 21**	**Day 28**	**Delivery (Week 36 gestation)**
**Informed consent**	X[2]								
**Smoking status/ CPD/ use of ecigs**		X			P[3]			X	X
**Cravings & tobacco withdrawal**		P			P			P	
**NRT concerns & necessity beliefs**		P						P	
**Saliva samples**		P			P				X
**Exhaled CO**		X							X
**NRT adherence data**					P			X	X
**Reported engagement with intervention**								X	
**NicUse app data collection**				←				→	
**Medical records data**									X
**Intervention Delivery (both trial arms)**									

For peer review only - http://bmjopen.bmj.com/site/about/guidelines.xhtml

**Counselling from SSPs offered**			X	X	X	X	X	X	
**NRT dispatch**			X		X	X	X	X	

[1] Randomisation follows baseline data collection
[2] X = data collected in both pilot and full trial phase
[3] P = data collected only in pilot phase

For peer review only

For peer review only

For peer review only - http://bmjopen.bmj.com/site/about/guidelines.xhtml

Baseline
We will ask participants about demographics, gestation, estimated date of delivery, partner smoking status, whether they smoked in previous pregnancies, nicotine dependence(30), current and pre-pregnancy smoking behaviours, prior experience using NRT, smoking beliefs, urges to smoke (cravings) and tobacco withdrawal symptoms.(31) Where possible, those recruited in person will also provide an expired air carbon monoxide (CO) reading at baseline. Researchers will also help participants to install the 'NicUse' smartphone app from Google Play and Apple Store (32), a bespoke app developed for SNAP2 on which applicants record daily smoking, e-cigarette and NRT use (see below) during the 28 -day intervention period. We will seek contact details for participants' GPs and check with them if there is any reason why a participant should not be enrolled.

Pilot phase only: All participants were sent an online questionnaire measuring concerns and necessity beliefs about NRT.(29) Those recruited in person were asked (at baseline) for a saliva sample and, if possible a CO reading given a self-return kit for collection and return of another saliva sample on Day 7 of their quit attempt. Those recruited online or remotely, were mailed two self-collection kits with instructions for returning one saliva sample immediately, and another on Day 7 of their quit attempt. Prior to collecting samples, we asked participants, verbally or by questionnaire in the postal return kit, when they last smoked, used an e-cigarette, if they are using NRT and which type(s) and, when this was last used.

Follow up
After baseline, apart from data collection by app and from routine sources, the primary data collection mode is by online questionnaires, with links texted or emailed to participants. If there is no response, first a reminder text and/or email will be sent, and then participants will be phoned, hard copy questionnaires with reply paid envelopes will be posted as a final option.

Daily NicUse recording of behaviours (between baseline & Day 28)
NicUse works on Android or Apple smartphones. Participants will be asked to use the app daily to record smoking behaviour, NRT and/or e-cigarette use. If participants miss reporting for one or two days, they can record these data retrospectively. Participants receive text messages to prompt completion of the app. The survey on NRT will ask participants to record patch and short-acting NRT use, and the number of units of short-acting NRT consumed, which allow us to calculate their daily nicotine dose(33). Compared to questionnaires, NicUse facilitates more complete collection of NRT adherence data, with more robust face validity, and is less likely to overestimate adherence than questionnaires(33).

Day 7 after quit date (pilot phase only)
We were asked if a quit attempt was made, if any NRT had been used since the quit date (QD) and if so, on how many days, which types, and if short-acting NRT is used, how many lozenges, cartridges or sprays were used. We also asked about current smoking, e-cigarette use, urges to smoke (cravings) and tobacco withdrawal symptoms. As soon as possible after Day 7, saliva samples (see above) were collected from participants. After the pilot, we discontinued data collection at this time point, as this information was only required for pilot phase outcomes.

Day 28 after quit date
We will use the same questions as in the pilot phase-only Day 7 follow up but will ask about the previous 28 (not 7) days, and we will ask the intervention group about BMN components (e.g. texts and website). Participants who do not provide information on smoking status or NRT use by app or questionnaire methods will be sent a text about NRT use since the quit date, and asked to reply directly.

For peer review only - http://bmjopen.bmj.com/site/about/guidelines.xhtml

Pilot phase only: Participants were asked to re-complete the NRT concerns and necessity beliefs measure they first completed at baseline.(29)

36 weeks gestation or delivery (if earlier)
We will ask participants about smoking, adherence to NRT since 28-day follow up and use of NHS stop smoking support. We will send non-responders a direct-reply text message asking about smoking in the previous 7 days.

Routine data collected from medical records
NHS hospitals collect smoking status and exhaled carbon monoxide from every woman from week 36 of gestation onwards and we will collect these data from NHS records. We will also extract maternal and foetal pregnancy outcome data from medical records. In NHS hospitals which are sites, research staff will collect these data; otherwise, the research team contact relevant NHS staff to ask for this information. Where birth outcome data cannot be obtained from records, using methods outlined above, we will ask participants to provide birth weight, gestation at delivery, and whether they underwent caesarean section or the baby was admitted to special care or had any congenital abnormality, and whether they were a smoker or non-smoker at delivery.

Validation of smoking abstinence
For participants who report 7-day smoking abstinence at 36 weeks or later, we will collect saliva samples as they attend hospital, at home visits, or postally (see above). Before giving samples, participants will be asked about any recent smoking or vaping or NRT use.

Financial incentives encouraging data return
To recognise time taken for study participation, participants will receive up to £50 in 'Love to Shop' gift cards which cannot be redeemed for tobacco or alcohol. To receive maximum remuneration participants will need to submit all adherence app data; they will receive 50p for each daily app report, and an additional £1.50 for supplying a continuous full week's reports, plus an additional £5 if they report for all 28 days after their quit date (maximum total £25). Further gift cards will be given to women if they provide requested questionnaire data and validation saliva samples.

Fidelity assessment (pilot phase only)
We will audio record all initial intervention group consultations, selecting a random sample for further scrutiny. Two researchers will listen to the selected audio recordings, independently rating the completeness of intervention delivery against a fidelity checklist which lists key components of the BMN intervention, and inter-rater reliability between researchers will be determined. We will store recordings on a secure University of Nottingham server for a maximum of seven years.

Data management
Each participant will be assigned a unique study identification number allocated at consent to identify their data and biological samples. Personal identifiers (name, email address and phone number) will be stored in a password-protected computer database accessible only by the researchers. Data will be entered into a REDCap database where possible, but paper CRFs may be used as source data and entered by researchers onto the database. Information submitted by participants via the NicUse app is stored as pseudonymised data on Amazon cloud.

All electronic data will be securely stored at the University of Nottingham for 15 years after which it will be destroyed. Data Management will be lead by York Trials Unit, with the support of the Trial Manager in University of Nottingham as detailed in the Data Management Plan.

For peer review only - http://bmjopen.bmj.com/site/about/guidelines.xhtml

The Trial Management Group (TMG) is responsible for the day to day running of the trial, meeting on a regular basis and is supported by, and report to, the Trial Steering Committee (TSC) (see supplementary file).

Saliva samples

Saliva samples will be collected by researchers or by participants and sent by post directly to ACM labs for storage and analysis at the end of the trial. The laboratory will quantify salivary cotinine concentrations and the presence or absence anabasine using a quantitative enzyme immunoassay technique (EIA). Once the analysis has been completed the saliva samples will be destroyed in accordance with the Human Tissue Act 2004.

Outcomes

Primary outcome

Reported smoking abstinence in late pregnancy or around childbirth, with appropriate biochemical validation.

Secondary outcomes

1. Reported smoking abstinence at *both* 28 days and in late pregnancy or at childbirth, with and without appropriate biochemical validation in late pregnancy.
2. Reported smoking abstinence for 24hrs and 7 days at 28 days.
3. Reported number of days NRT is used in the first 28 days following a quit date.
4. Reported mean daily nicotine dose in the first 7 days of quitting ('intensity' of NRT use)
5. Engagement with BMN intervention components

Pilot phase outcomes

6. Urges to smoke, 'cravings', and tobacco withdrawal symptoms.
7. NRT concerns and necessity beliefs at baseline and Day 28.
8. Fidelity of intervention delivery as measured against fidelity checklist.

Other outcomes

9. Saliva cotinine concentration
10. Number of days NRT use between a quit date and the end of pregnancy.
11. Exhaled carbon monoxide (CO) concentration
12. Birth weight
13. Low birth weight (<2500g)
14. Gestational age at birth
15. Maternal or foetal death (stillbirth or miscarriage).
16. Caesarean section delivery
17. Neonatal intensive care admission
18. Congenital anomaly

Sample size and justification

Design changes: Originally, we planned SNAP2 as an RCT to test the extent to which 'Baby, Me and NRT' (BMN) did or did not increase adherence to NRT; adherence to NRT was intended as the primary outcome and smoking outcomes as secondary. If study findings gave a sufficiently positive 'signal' for an effect on NRT adherence, we planned a second RCT to test whether BMN had positive effects on smoking cessation. However, due to the Covid pandemic and NHS service provision changes, the funder agreed that BMN efficacy for smoking abstinence would be better investigated by instead simply using a measure of smoking behaviour as the trial primary outcome and increasing the SNAP2 sample size. This was dependant on a successful pilot phase of the trial in which i) BMN demonstrated a sufficiently large 'signal' that impacts on adherence to NRT, and ii) explanatory trial

For peer review only - http://bmjopen.bmj.com/site/about/guidelines.xhtml

outcomes data were collected prior to being discontinued in the more pragmatic 'full' trial . Below we detail how, in the SNAP2 pilot phase, the impact of BNM on adherence to NRT assessed, and progression decided upon.

Assessment of BMN's potential effect on NRT adherence': For the original SNAP2 RCT, we had defined a clinically important effect as BMN increasing NRT use by 21%. Data from a previous study indicated that, if the control group was offered NRT for 28 days, they were likely to use this on a mean of 7 days(34), so a 21% increase represented an extra 1-2 days of NRT use.

We used a one-sided confidence interval approach(35) to assess whether or not the pilot phase SNAP2 trial 'signal' for the impact of BMN on NRT adherence was consistent with assumed effects. Using a confidence interval approach, we calculated that for the checkpoint analysis to produce an upper limit of a one-sided 80% or 90% confidence interval which excludes the estimate of effect, assuming the treatment estimate from the checkpoint assessment was zero or less would require 34 or 54 in the analysis. A trial statistician estimated BMN efficacy using data from the 49 participants followed up to the primary outcome point. The point estimate, and the upper confidence limit (whether 80% or 90%) was greater than the pre-specified clinically relevant effect size of 1.21. The funder deemed this sufficient demonstration of potential efficacy for the pilot to progress to a full trial.

Full trial sample size estimate: To determine the sample size for the full trial, we assume a control group quit rate of 10%, consistent with similar UK studies(34);(36, 37), and seek to detect an absolute increase in risk of abstinence of 6% (corresponding to a risk ratio (RR) of 1.6). We think this is a reasonable effect size to seek as all previous trials of NRT used by pregnant women tested use of only one NRT product and these show a relatively weak, imprecise effect (RR 1.37, 95% CI 1.08 to 1.74),(13) with smaller point estimates seen in the least biased studies (RR 1.21, 95% CI 0.95 to 1.55).(13) However, in non-pregnant smokers where adherence to NRT is much stronger, depending on the type of NRT used, Cochrane review point estimates for NRT efficacy range between 1.49 (for gum) and 2.02 (inhalator)(38) and dual NRT (patch + fast acting) is more effective than single product use, with an OR for abstinence with NRT of 1.25 (dual versus single product NRT use).(12) We aim to recruit 1,320 participants, providing a minimum of 90% power for a two-sided test of size 5%, assuming the control group quit rate is at least 10%.

Analyses

Statistical analysis will be conducted when the trial ends using Stata/MP v18 or later unless specified otherwise. All analysis software used including any community contributed software will be explicitly cited in any publication of the trial results. Significance tests will be two-sided at the 5% level unless specified otherwise. Point estimates will be presented with their associated 95% confidence intervals. Full analyses will be detailed in a statistical analysis plan (SAP), finalised prior to the end of data collection, and which will be reviewed by the TSC. A CONSORT flow diagram will be provided to display the flow of participants through the study. Baseline data will be summarised descriptively by group, for all randomised and for all those who are included in the primary analysis. No formal statistical comparisons of group differences at baseline will be conducted. Continuous measures will be reported as means and standard deviation, while the categorical data will be reported as counts and percentages.

Assessment of efficacy

The primary efficacy endpoint is reported abstinence in late pregnancy or around childbirth, validated by appropriate biochemical measures (binary – abstinent or non-abstinent). The primary analysis model will include all randomised participants as part of the groups to which they were allocated, with any missing primary endpoints imputed as being non-abstinent. We

For peer review only - http://bmjopen.bmj.com/site/about/guidelines.xhtml

will compare abstinence between treatment groups using logistic regression, with a fixed effects of treatment group, gestation at baseline (the stratification factor), and other predictive baseline covariates (pre-specified in the SAP). In addition to the point and interval estimate of the OR for allocation, we will use the fitted model to obtain estimates of treatment effects on both the relative risk and risk difference scales. Binary secondary outcomes (e.g. other abstinence outcomes) will be analysed in a broadly similar manner to the analysis of the primary outcome. Secondary outcomes including adherence to NRT, NRT adherence intensity, cotinine (smoking exposure), necessities/concerns and birth outcomes will be compared between arms using appropriate generalised linear models. All other outcomes will be summarised descriptively by randomised arm. If the relevant data are sufficiently complete, we will undertake exploratory analyses to decompose the total effect of allocation on the primary outcome into indirect effects (i.e. those mediated by improved NRT adherence/usage) and direct effects (i.e. those not mediated by improved NRT adherence/usage).

Procedures for missing, unused and spurious data

We will assume that participants with missing smoking status are smoking, in line with the Russell Standard, meaning people who do not provide data are assumed to be smoking(39). Similarly, participants not providing a NRT usage response(s) will be assumed to be not using NRT in the corresponding time period. We will investigate the sensitivity of results from the main analyses to departures from these strict missing not at random (MNAR) assumptions, by imputing these missing data under a range of missing at random (MAR) and MNAR scenarios.

Safety

The study tests a behavioural intervention aimed at optimising NRT use.(26) As this is a standard NHS treatment, we do not anticipate any harm being caused and there is no adverse event monitoring.

Economic analysis

The economic analysis will determine the cost-effectiveness using a lifetime time horizon. To estimate long term benefits, costs and cost-effectiveness, and potential longer-term cost savings we will use the Economics of Smoking in Pregnancy (ESIP) model, a bespoke, dynamic economic model designed specifically for valuing smoking cessation in pregnancy in economic terms.(40);(41) Smoking cessation rates from the trial and costs of intervention delivery and usual care will be used as ESIP inputs. As SNAP 2 has a short follow up period, we will not use measures of participants' Quality of Life as one would not expect changes in response to the SNAP2 intervention within the short study timeframe, and any QoL changes resulting from smoking cessation would likely be variation in QoL reflecting physiological changes in pregnancy.

Patient and public involvement

A User and Public Involvement Advisory Group has been set up to for our N-READY research programme; the current trial is part of this programme. This advisory group is made up of women who currently smoke, who have smoked at any point during pregnancy, or who are of childbearing age and smoke / are ex-smokers. This Advisory Group contributes to all stages of the study, from reviewing study related documentation and materials to dissemination of research findings. Intervention development, via previous workstreams of the N-READY programme, has already been heavily informed by this group as well as by interviews with women who smoke (or have smoked) during pregnancy.

ETHICS AND DISSEMINATION

Ethics approval has been granted by the Bloomsbury NHS Research Ethics Committee (21/LO/0123). Written informed consent will be obtained from all participants. The findings

For peer review only - http://bmjopen.bmj.com/site/about/guidelines.xhtml

will be disseminated to the public, the funders, relevant practice and policy representatives and other researchers. A data sharing agreement has been published; once the trial has finished and the main trial paper has published, a fully anonymised trial data set will be available on reasonable request from York Trials Unit.

Trial and recruitment status

Recruitment began in June 2021. We completed recruitment of 264 participants, 100% of target sample size, to the pilot study in October 2022. The main study aims to recruit 1320 participants by December 2025 with current recruitment on track to achieve this, and follow up to continue for a further 10 months with last participant last visit due in October 2026.

Due to the pandemic, recruitment via traditional routes became problematic. In response to these difficulties, we have successfully recruited via paid social media advertising. Of the first 265 participants 185 (69.8%) were recruited via hospital antenatal settings and 80 (30.2%) via online recruitment routes.

For peer review only

Contributors

TC, SC, FN, MU, LB, PA, DT, IB, SL, SP, SS, AMcE, KV conceived the study project and obtained project funding. TC, MC, AD, RT, JE, LMCD, LP, FN, MU, SS and JB contributed to the design of the intervention Baby, Me and NRT. TC led on the protocol development and TC and MC drafted the manuscript, with contributions from all authors. AD, LP, RT, TC, SC, MC, JE, LMCD, LP and FN contributed to the fidelity assessment of the intervention. SP led on the health economics section. SL, CH, CW, and GP contributed to the statistical analysis section. KB, MC, and LP contributed to the regulatory and data management content. All authors contributed to the preparation and drafting of the manuscript.

Sponsor

University of Nottingham, bb-sponsor@nottingham.ac.uk is the sponsor of the study and provides indemnity insurance. Roles and responsibilities of trial committees and coordinating centres are provided in the supplementary information.

Funding

This paper presents independent research funded by the National Institute for Health Research (NIHR) under its Programme Grants for Applied Research [Grant Reference Number RP-PG-0615-20003]. The views expressed are those of the authors and not necessarily those of the NIHR or the Department of Health and Social Care. Tim Coleman is an NIHR Senior Investigator.

Competing interests

FN is an unpaid member of the scientific committee for the Smoke Free app, a smoking cessation app unrelated to this project. All other authors have declared that they have no competing interests.

References

1. NHS Digital. Statistics on Women's Smoking Status at Time of Delivery: England, Quarter 2, 2023-24 2024 [Available from: https://digital.nhs.uk/data-and-information/publications/statistical/statistics-on-women-s-smoking-status-at-time-of-delivery-england/statistics-on-womens-smoking-status-at-time-of-delivery-england-quarter-2-2023-24.

For peer review only - http://bmjopen.bmj.com/site/about/guidelines.xhtml

2. Chamberlain C, O'Mara-Eves A, Porter J, Coleman T, Perlen SM, Thomas J, et al. Psychosocial interventions for supporting women to stop smoking in pregnancy. Cochrane Database Syst Rev. 2017(2).
3. Cnattingius S. The epidemiology of smoking during pregnancy: smoking prevalence, maternal characteristics, and pregnancy outcomes. Nicotine Tob Res. 2004;6(S2):S125-S40.
4. Leonardi-Bee J, Jere ML, Britton J. Exposure to parental and sibling smoking and the risk of smoking uptake in childhood and adolescence: a systematic review and meta-analysis. Thorax. 2011;66(10):847-55.
5. World Health Organization. Report on the Global Tobacco Epidemic 2008—the mpower package. Geneva: World Health Organization; 2008.
6. Cooper S, Orton S, Leonardi-Bee J, Brotherton E, Vanderbloemen L, Bowker K, et al. Smoking and quit attempts during pregnancy and postpartum: a longitudinal UK cohort. BMJ Open. 2017;7(11).
7. National Institute for Health and Clinical Excellence. Quitting smoking in pregnancy and following childbirth. NICE Public Health Guidance 26. London; 2010. Report No.: PH26.
8. National Institute for Clinical Excellence. Tobacco: preventing uptake, promoting quitting and treating dependence. NICE guideline [NG209]. https://www.nice.org.uk/guidance/ng209/chapter/Recommendations-on-treating-tobacco-dependence-in-pregnant-women#providing-support-to-stop-smoking. 2021.
9. Fahy SJ, Cooper S, Coleman T, Naughton F, Bauld L. Provision of smoking cessation support for pregnant women in England: results from an online survey of NHS stop smoking services for pregnant women. BMC Health Services Research. 2014;14(1):107.
10. Cooper S, Orton S, Campbell K, Ussher M, Coleman-Haynes N, Whitemore R, et al. Attitudes to E-Cigarettes and Cessation Support for Pregnant Women from English Stop Smoking Services: A Mixed Methods Study. International Journal of Environmental Research and Public Health. 2019;16(1):110.
11. Dhalwani NN, Szatkowski L, Coleman T, Fiaschi L, Tata LJ. Prescribing of nicotine replacement therapy in and around pregnancy: a population-based study using primary care data. Br J Gen Pract. 2014;64(626):e554-60.
12. Lindson N, Chepkin SC, Ye W, Fanshawe TR, Bullen C, Hartmann-Boyce J. Different doses, durations and modes of delivery of nicotine replacement therapy for smoking cessation. Cochrane Database Syst Rev. 2019;4:CD013308.
13. Claire R, Chamberlain C, Davey MA, Cooper SE, Berlin I, Leonardi-Bee J, et al. Pharmacological interventions for promoting smoking cessation during pregnancy. Cochrane Database of Systematic Reviews. 2020(3).
14. Hollands GJ, Sutton S, McDermott MS, Marteau TM, Aveyard P. Adherence to and consumption of nicotine replacement therapy and the relationship with abstinence within a smoking cessation trial in primary care. Nicotine Tob Res. 2013;15(9):1537-44.
15. Bowker K, Lewis S, Coleman T, Cooper S. Changes in the rate of nicotine metabolism across pregnancy: a longitudinal study. Addiction. 2015;110(11):1827-32.
16. Dempsey D, Jacob P, III., Benowitz NL. Accelerated metabolism of nicotine and cotinine in pregnant smokers. J Pharmacol Exp Ther. 2002;301(2):594-8.
17. Bowker K, Campbell KA, Coleman T, Lewis S, Naughton F, Cooper S. Understanding pregnant smokers' adherence to nicotine replacement therapy during a quit attempt: a qualitative study. Nicotine Tob Res. 2015.
18. Campbell K, Coleman-Haynes T, Bowker K, Cooper SE, Connelly S, Coleman T. Factors influencing the uptake and use of nicotine replacement therapy and e-cigarettes in pregnant women who smoke: a qualitative evidence synthesis. Cochrane Database Syst Rev. 2020;5:CD013629.

For peer review only - http://bmjopen.bmj.com/site/about/guidelines.xhtml

19. Thomson R, McDaid L, Emery J, Naughton F, Cooper S, Dyas J, et al. Knowledge and Education as Barriers and Facilitators to Nicotine Replacement Therapy Use for Smoking Cessation in Pregnancy: A Qualitative Study with Health Care Professionals. Int J Environ Res Public Health. 2019;16(10):1814.
20. Fergie L, Campbell KA, Coleman-Haynes T, Ussher M, Cooper S, Coleman T. Stop smoking practitioner consensus on barriers and facilitators to smoking cessation in pregnancy and how to address these: A modified Delphi survey. Addictive Behaviors Reports. 2019;9:100164.
21. Fergie L, Coleman T, Ussher M, Cooper S, Campbell KA. Pregnant Smokers' Experiences and Opinions of Techniques Aimed to Address Barriers and Facilitators to Smoking Cessation: A Qualitative Study. Int J Environ Res Public Health. 2019;16(15):2772.
22. Benowitz NL, Dempsey DA, Goldenberg RL, Hughes JR, Dolan-Mullen P, Ogburn PL, et al. The use of pharmacotherapies for smoking cessation during pregnancy. Tob Control. 2000;9 Suppl 3:III91-III4.
23. Taylor L, Claire R, Campbell K, Coleman-Haynes T, Leonardi-Bee J, Chamberlain C, et al. Fetal safety of nicotine replacement therapy in pregnancy: systematic review and meta-analysis. Addiction. 2020.
24. Coleman T, Cooper S, Thornton JG, Grainge MJ, Watts K, Britton J, et al. A randomized trial of nicotine-replacement therapy patches in pregnancy. N Engl J Med. 2012;366(9):808-18.
25. Cooper S, Taggar J, Lewis S, Marlow N, Dickinson A, Whitemore R, et al. Effect of nicotine patches in pregnancy on infant and maternal outcomes at 2 years: follow-up from the randomised, double-blind, placebo-controlled SNAP trial. The Lancet Respiratory Medicine. 2014;2(9):728-37.
26. McDaid L, Emery J, Thomson R, Coleman T, Cooper S, Dickinson A, et al. Development of "Baby, Me, & NRT": A Behavioral Intervention to Improve the Effectiveness of Nicotine Replacement Therapy in Pregnancy. Nicotine & tobacco research : official journal of the Society for Research on Nicotine and Tobacco. 2023;25(11):1770-80.
27. Chan AW, Tetzlaff Jm Fau - Gøtzsche PC, Gøtzsche Pc Fau - Altman DG, Altman Dg Fau - Mann H, Mann H Fau - Berlin JA, Berlin Ja Fau - Dickersin K, et al. SPIRIT 2013 explanation and elaboration: guidance for protocols of clinical trials. (1756-1833 (Electronic)).
28. Papadakis S, Hermon Y, McEwan A. Standard Treatment Programme for Pregnant Women: A guide to behavioural support for smoking cessation during pregnancy and the post-partum period: National Centre for Smoking Cessation and Training (NCSCT); 2019. Available from: https://www.ncsct.co.uk/usr/pub/NCSCT%20Standard%20Treatment%20Programme%20for%20Pregnant%20Women.pdf.
29. Emery J, McDaid L, Coleman T, Cooper S, Thomson R, Kinahan-Goodwin D, et al. Development and Content Validation of a Questionnaire for Measuring Beliefs About Using Nicotine Replacement Therapy for Smoking Cessation in Pregnancy. Nicotine Tob Res. 2023;25(7):1310-8.
30. Heatherton TF, Kozlowski LT, Frecker RC, Rickert W, Robinson J. Measuring the heaviness of smoking: using self-reported time to the first cigarette of the day and number of cigarettes smoked per day. British Journal of Addiction. 1989;84(7):791-9.
31. West R, Hajek P. Evaluation of the mood and physical symptoms scale (MPSS) to assess cigarette withdrawal. Psychopharmacology (Berl). 2004;177(1-2):195-9.
32. Huang Y, Emery J, Naughton F, Cooper S, McDaid L, Dickinson A, et al. The development and acceptability testing of an app-based smart survey system to record

For peer review only - http://bmjopen.bmj.com/site/about/guidelines.xhtml

smoking behaviour, use of nicotine replacement therapy (NRT) and e-cigarettes. BMC Res Notes. 2022;15(1):100.
33. Emery J, Huang Y, Naughton F, Cooper S, McDaid L, Dickinson A, et al. Comparison of a Daily Smartphone App and Retrospective Questionnaire Measures of Adherence to Nicotine Replacement Therapy Among Pregnant Women: Observational Study. JMIR Form Res. 2023;7:e35045.
34. Coleman T, Cooper S, Thornton JG, Grainge MJ, Watts K, Britton J, et al. A randomized trial of nicotine-replacement therapy patches in pregnancy 8. NEnglJMed. 2012;366(9):808-18.
35. Cocks K, Torgerson DJ. Sample size calculations for pilot randomized trials: a confidence interval approach. J Clin Epidemiol. 2013;66(2):197-201.
36. Ussher M, Lewis S, Aveyard P, Manyonda I, West R, Lewis B, et al. Physical activity for smoking cessation in pregnancy: randomised controlled trial. British Medical Journal. 2015;350.
37. Tappin D, Bauld L, Purves D, Boyd K, Sinclair L, MacAskill S, et al. Financial incentives for smoking cessation in pregnancy: randomised controlled trial. BMJ. 2015;350.
38. Hartmann-Boyce J, Chepkin SC, Ye W, Bullen C, Lancaster T. Nicotine replacement therapy versus control for smoking cessation. Cochrane Database of Systematic Reviews. 2018(5).
39. West R, Hajek P, Stead L, Stapleton J, West R, Hajek P, et al. Outcome criteria in smoking cessation trials: proposal for a common standard. Addiction. 2005;100(3):299-303.
40. Jones M, Smith M, Lewis S, Parrott S, Coleman T. A dynamic, modifiable model for estimating cost-effectiveness of smoking cessation interventions in pregnancy: application to an RCT of self-help delivered by text message. Addiction. 2018;0(0).
41. Coleman T, Clark M, Welch C, Whitemore R, Leonardi-Bee J, Cooper S, et al. Effectiveness of offering tailored text message, self-help smoking cessation support to pregnant women who want information on stopping smoking: MiQuit3 randomised controlled trial and meta-analysis. Addiction. 2021.

Figure 1. Study flowchart

For peer review only - http://bmjopen.bmj.com/site/about/guidelines.xhtml

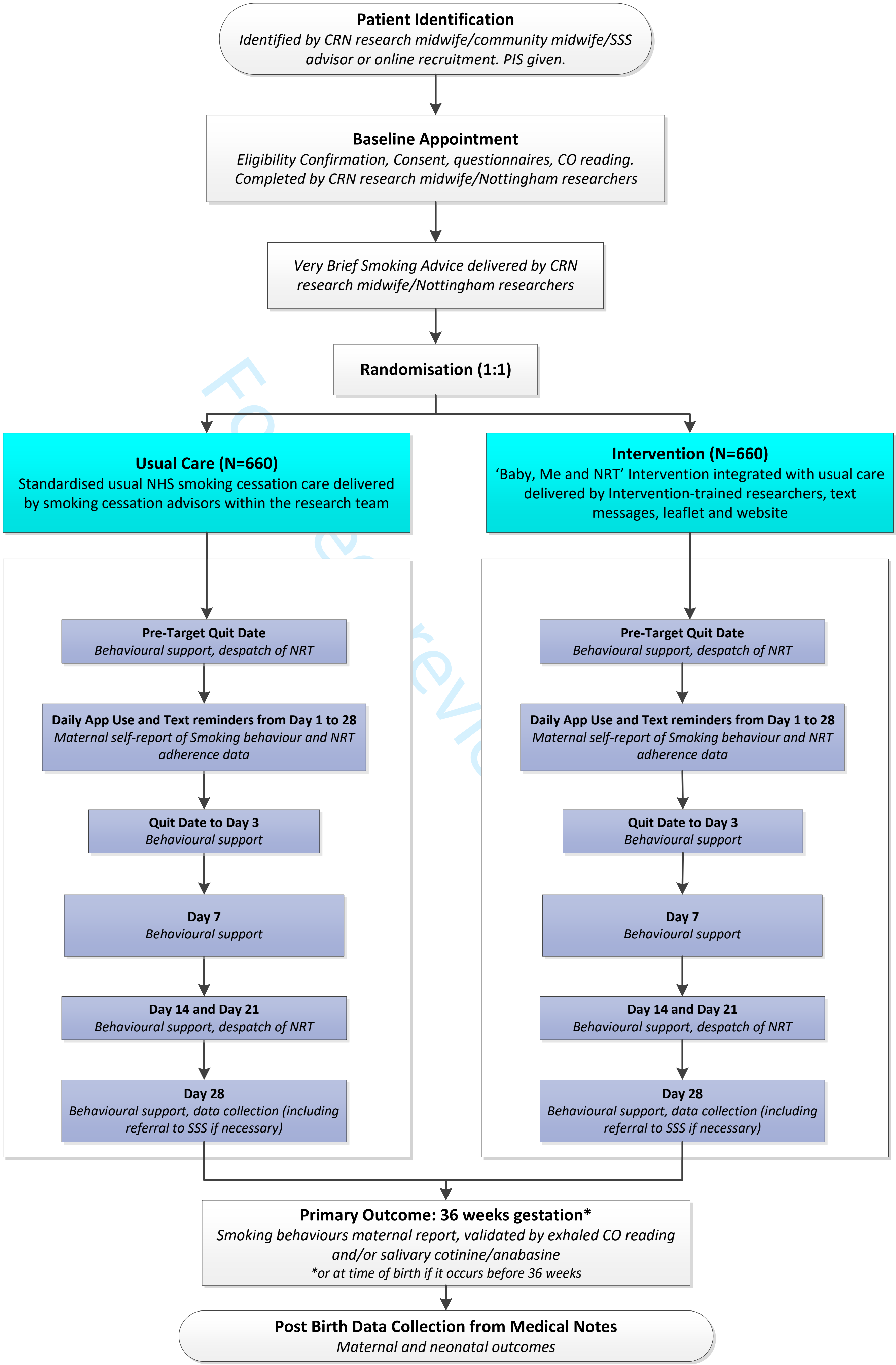
Patient Identification
Identified by CRN research midwife/community midwife/SSS advisor or online recruitment. PIS given.
Baseline Appointment
Eligibility Confirmation, Consent, questionnaires, CO reading. Completed by CRN research midwife/Nottingham researchers
Very Brief Smoking Advice delivered by CRN research midwife/Nottingham researchers
Randomisation (1:1)
Usual Care (N=660)
Standardised usual NHS smoking cessation care delivered by smoking cessation advisors within the research team
Intervention (N=660)
'Baby, Me and NRT' Intervention integrated with usual care delivered by Intervention-trained researchers, text messages, leaflet and website
Pre-Target Quit Date
Behavioural support, despatch of NRT
Daily App Use and Text reminders from Day 1 to 28
Maternal self-report of Smoking behaviour and NRT adherence data
Quit Date to Day 3
Behavioural support
Day 7
Behavioural support
Day 14 and Day 21
Behavioural support, despatch of NRT
Day 28
Behavioural support, data collection (including referral to SSS if necessary)
Pre-Target Quit Date
Behavioural support, despatch of NRT
Daily App Use and Text reminders from Day 1 to 28
Maternal self-report of Smoking behaviour and NRT adherence data
Quit Date to Day 3
Behavioural support
Day 7
Behavioural support
Day 14 and Day 21
Behavioural support, despatch of NRT
Day 28
Behavioural support, data collection (including referral to SSS if necessary)
Primary Outcome: 36 weeks gestation*
Smoking behaviours maternal report, validated by exhaled CO reading and/or salivary cotinine/anabasine
*or at time of birth if it occurs before 36 weeks
Post Birth Data Collection from Medical Notes
Maternal and neonatal outcomes

Roles and responsibilities

These membership lists are correct at the time of writing:

Roles of trial sponsor and funders

Name	Affiliation	Role
Alison Thorpe	University of Nottingham ('Sponsor')	Representative of the sponsor
Thomas Hutchinson	NIHR ('Funder')	Representative of the funder

Trial Team

Name	Affiliation	Role
Tim Coleman	University of Nottingham	Chief Investigator
Sue Cooper	University of Nottingham	Programme lead for Workstream 3.
Miranda Clark	University of Nottingham	Senior Trial Manager, trial management oversight
Kate Bowker	University of Nottingham	Trial Manager, day to day running of the trial
Lucy Phillips	University of Nottingham	Trial Manager, day to day running of the trial
Karen Daykin/ Nicki Stockdale	University of Nottingham	Trial Manager/Trial Coordinator, day to day running of the trial
Anne Dickinson	University of Nottingham	Researcher, trial intervention delivery and management of the delivery team
Daniel Robertson	University of Nottingham	Trial Coordinator, day to day running of the trial and participant follow up
Kasia Kowalewska	University of Nottingham	Trial Coordinator, day to day running of the trial and participant follow up
Anne Dickinson	University of Nottingham	Researcher, trial intervention delivery and management of the delivery team
Amy Morton	University of Nottingham	Trial administration, participant follow up
Eleanor Holmes	University of Nottingham	Trial administration, participant follow up
Katie Zhoya	University of Nottingham	Trial administration, participant follow up
Michelle Rawding	University of Nottingham	Trial administration, participant follow up
Daniel Simpkins		Senior Data Manager responsible for management of the database
Sarah Gardner	University of York	Database design and build
Matthew Bailey	University of York	Trial database set up and randomisation system build
Ross Thompson	University of Nottingham	Researcher, participant recruitment/consent
Lisa McDaid	University of East Anglia	Researcher, development of the intervention, participant recruitment/consent
Jo Emery	University of East Anglia	Researcher, development of the intervention, participant recruitment/consent
Felix Naughton	University of East Anglia	Programme lead for Workstream 1 and 2 of the NREADY programme

Trial Management Group

Name	Affiliation	Role
Tim Coleman	University of Nottingham	Chief Investigator
Sue Cooper	University of Nottingham	Programme manager
Miranda Clark	University of Nottingham	Senior Trial Manager
Kate Bowker	University of Nottingham	Trial Manager, day to day running of the trial
Lucy Phillips	University of Nottingham	Trial Manager, day to day running of the trial
Karen Daykin/ Nicki Stockdale	University of Nottingham	Trial Manager/Trial Coordinator, day to day running of the trial

For peer review only

Anne Dickinson	University of Nottingham	Researcher, trial intervention delivery and management of the delivery team
Ross Thomson	University of Nottingham	Researcher, participant recruitment/consent
Catherine Hewitt	University of York	Lead Trial statistician
Charlie Welch	University of York	Trial statistician
Gill Parkinson	University of York	Trial statistician
David Torgeson	University of York	Director of the York Trials Unit
Michael Ussher	St Georges, University of London	Population Health Science, and Social Marketing and Health
Sarah Lewis	University of Nottingham	Independent statistician

Trial Steering Committee

Name	Affiliation	Role
Peter Hajek	Queen Mary University London	Independent Chair
Martyn Willmore	Public Health England	Independent member
Jo Locker	Public Health England	Independent member
Donna Wilkes	PPI	Independent PPI Representative
Nikki Totton	University of Sheffield	Medical Independent statistician
Alison Thorpe	University of Nottingham ('Sponsor')	Observer (sponsor)
Thomas Hutchinson	NIHR	Observer (funder)
Catherine Hewitt	Lead Trial statistician (York Trials Unit)	Observer
Charlie Welch	Trial statistician (York Trials Unit)	Observer
Gill Parkinson	Trial statistician (York Trials Unit)	Observer
Tim Coleman	University of Nottingham	Non-independent member
Sue Cooper	University of Nottingham	Observer
Miranda Clark	University of Nottingham	Observer
Trial Manager	University of Nottingham	Observer
Felix Naughton	University of East Anglia	Observer

For peer review only

Participant Information Sheet
(Version 2.1 Date 12.09.22)

IRAS Project ID: 287771

Title of Study: **Smoking, Nicotine and Pregnancy 2 Trial (SNAP 2)**

Name of Chief Investigator: Tim Coleman

We would like to invite you to take part in our research study. Before you decide we would like you to understand why the research is being done and what it would involve for you. Please read the information below carefully. One of our team will go through the information sheet with you before you decide to take part and answer any questions you have. Talk to family, friends, or others about the study if you wish. Please ask us if there is anything that is not clear.

What is the purpose of the study?

- We want to improve the support that pregnant women receive to help them stop smoking.
- Pregnant women can use Nicotine Replacement Therapy (NRT) to help them stop smoking and the NHS prescribes this to them for free.
- However, pregnant women often do not use NRT in the best possible way and this can make it less effective than it could be.
- Therefore, in this study, we are testing a package of support which we hope will help pregnant women make better use of NRT so, it will have a better chance of helping them to stop smoking.

Why have I been invited?
We are inviting you to take part because you have told us that you are less than 25 weeks pregnant, smoke and are interested in getting help to quit, like NRT.

Do I have to take part?
It is up to you to decide whether or not to take part. If you do take part you will be given this information sheet to keep and will be asked to complete a consent form on paper, online or by telephone. If you join the study, you will be free to withdraw at any time without giving a reason. This would not affect your legal rights.

What will happen to me if I take part?
A computer will randomly place you into one of two groups with an equal chance of being in either.

Group One will receive support to stop smoking, which is the same as usual NHS support. You will be offered NRT as a patch, short acting NRT such as lozenges, inhalator or mouth spray or both together ('dual NRT'). You will receive up to six support sessions with a stop smoking practitioner (SSP). The first session will take place just before the day on which you stop smoking (quit date) and will last approximately *30 minutes*. Further consultations will be offered on or around *Day 3, Day 7, Day 14, Day 21, and Day 28* after your quit date. These will take place by telephone or video call and will last approximately *15 minutes* each.

Group Two will receive the same usual NHS stop smoking support that women in Group One receive, plus an intervention to help them make better use of NRT. This includes special support from a stop smoking practitioner, a leaflet, text messages, and a website. The first session will be just before the quit date and will last no longer than *45 minutes* and follow up consultations will last approximately *15 minutes* each and take place by telephone or video call.

For peer review only - http://bmjopen.bmj.com/site/about/guidelines.xhtml

Having two groups is a very important because it allows us to compare them and to learn about any benefits or disadvantages of the support we are testing. Joining the study will not affect your usual care and, should you decide not to participate, you will be offered the usual NHS support for stopping smoking which is available to you locally.

What would we expect from everyone taking part?

We will contact you by telephone, videocall, email, text or post. For some of the research information, we will send you a link by email or text asking you to complete a short questionnaire online:

- **When you first join the study.**

  We will ask questions about smoking and NRT. This should take no longer than *10 minutes*.

  We may ask you to provide a breath sample to measure your smoking. If you are selected to provide a breath test, we may send you a carbon monoxide meter and we will help you to set up an app on your mobile phone which helps you to record a carbon monoxide reading by blowing into this . Providing breath samples should take no longer than *5 minutes*.

  We will help you to download the NicUse app to your mobile phone. For each of the 28 days after your quit date, we will ask you to tell the app about your NRT use and any smoking and / or e-cigarette use. If you do not answer app questions for 2 days in a row, we will send a text message reminder.

- **Day 28 after your quit date**. We will ask about smoking and NRT and how you have got on providing a breath sample and using the app. If you are in Group 2, we will ask you some additional questions about your experiences of using the website, leaflet and receiving text messages; this should take no longer than *10 minutes*.

- **Towards the end of your pregnancy.** At around 36 weeks, we will ask you some questions about your smoking and NRT use. We may also ask you to provide saliva and breath samples. This should take no longer than *10 minutes*.

We will liaise with the hospital you are booked to deliver your baby to check how your pregnancy is progressing at approximately 34 weeks into your pregnancy, and again at a later date to find out details about the birth of your baby and your smoking status around delivery if required. We may contact you to ask you for information about the birth of your baby and your smoking status around delivery.

We will ask your permission to audio record some of your consultations with the stop smoking practitioner; this will help us monitor the quality of the support that we provide. This is optional. You will be able to take part in the study without agreeing to being recorded but, it would be helpful to the study if you were to agree. Audio recordings of consultations will be kept confidential. Only members of the research team will have access to these.

For peer review only - http://bmjopen.bmj.com/site/about/guidelines.xhtml

Below is a summary of what we would expect from you if you decided to take part in this study.

Research collection and stop smoking practitioner schedule

Baseline contact with advisor, research data collection and breath sample. Agree quit date and NRT.		Day 1: Quit Date APP	Day 2 APP	Day 3: Advisor contact APP	Day 4 APP
Day 5 APP	Day 6 APP	Day 7: Advisor contact APP SMS	Day 8 APP	Day 9 APP	Day 10 APP
Day 11 APP	Day 12 APP	Day 13 APP	Day 14: Advisor contact APP	Day 15 APP	Day 16 APP
Day 17 APP	Day 18 APP	Day 19 APP	Day 20 APP	Day 21: Advisor contact APP	Day 22 APP
Day 23 APP	Day 24 APP	Day 25 APP	Day 26 APP	Day 27 APP	Day 28: Final advisor contact, research data collection. APP

Support we give to you	Advisor telephone support	28 Day Supply of Nicotine Replacement Therapy (NRT)			
What we ask from you	Saliva sample	Breath sample	Use a mobile phone app (APP)	Research data collection	
36 weeks we will collect & possibly a further and					

Expenses

All texts we send you are free, but texts you send to us will be the same as texting from a UK mobile number. Please check with your mobile phone provider about text messaging charges.

What are the possible disadvantages and risks of taking part?

We do not foresee there being any risks from taking part in this study. However, we appreciate that taking part will use your time and may therefore be inconvenient. Also, if you are likely to be upset by receiving some basic information about the risks of smoking in pregnancy then it is best not to take part.

What are the possible benefits of taking part?

We cannot promise the study will help you, but all participants will receive support to stop smoking based on the best NHS standards of practice. The information you provide to us during the study will be invaluable in helping us devise ways of supporting women like you who want to stop smoking during their pregnancy.

What happens when the research study stops?

Once your involvement in the study ends you will continue to receive routine stop smoking support available to NHS patients in the locality, unless you choose not to. We can assist you with this. If you are interested in reading the findings from this study, you can agree for us to keep your contact details after the end of the study, so that we can share the overall results with you once these are available.

For peer review only - http://bmjopen.bmj.com/site/about/guidelines.xhtml

What if there is a problem?

If you have a concern about any aspect of the study, you should ask to speak with the study team in Nottingham who will do their best to answer your questions. If you remain unhappy and wish to complain formally, you can do this through the NHS Complaints Procedure. For advice on making a complaint, contact your local Patient Advice and Liaison Service (PALS) at your local hospital.

PALS offers confidential advice, support and information on health-related matters and can provide patients with more information about the complaints procedure and the Independent Complaints Advocacy Service (ICAS).

Will my taking part in the study be kept confidential?

We will follow ethical and legal practice and all information about you will be handled in confidence.
If you join the study, we will use information collected from you and your medical notes during the course of the research. All data will be kept **strictly confidential**, stored in a secure and locked office, and on a password protected database at the University of Nottingham or with trial colleagues at the University of Cambridge. Research data shared with individuals from other Universities who are working within our research team will not have access to identifiable data. Any information shared will use a unique personalised participant study number. Under UK Data Protection laws the University is the Data Controller (legally responsible for the data security) and the Chief Investigator of this study (named at the start of this document) is the Data Custodian (manages access to the data). The University of Nottingham is the data controller for the study. This means we are responsible for looking after your information and using it properly. Your rights to access, change or move your information are limited, as we need to manage your information in specific ways to comply with certain laws and for the research to be reliable and accurate. To safeguard your rights we will use the minimum personally-identifiable information possible.

For peer review only

You can find out more about how we use your information and to read our privacy notice at: https://www.nottingham.ac.uk/utilities/privacy.aspx.

The data collected for the study will be looked at and stored by authorised persons from the research team. They may also be looked at by authorised people from regulatory organisations to check that the study is being carried out correctly. All will have a duty of confidentiality to you as a research participant and we will do our best to meet this duty. Audio recordings will be anonymised and will be accessed by members of the research team. Only members of the research team will have access to any audio recordings where you could be identified. Anonymised transcripts and personal details will be stored separately on a secure network.

If you consent, your contact information will be kept by the University of Nottingham for up to 3 years after the end of the study so that we are able to contact you about the findings of the study and possible follow-up studies. This information will be kept separately from the research data collected and only those who need to will have access to it. All research data will be kept securely for 7 years or longer if required. After this time your data will be disposed of securely. During this time all precautions will be taken by all those involved to maintain your confidentiality, only members of the research team given permission by the data custodian will have access to your personal data.

In order for you to receive the text messaging service, your mobile phone number will be shared with a text carrier called FastSMS and/or Esendex, after the study is completed your confidential information will permanently deleted from these carriers. Their full information security statement can be found here: https://fastsms.co.uk/downloads/fastsms-privacy-policy.pdf and https://www.esendex.co.uk/knowledge-hub/faqs/.

In accordance with the University of Nottingham's, the Government's and our funders' policies we may share our research data with researchers in other Universities and organisations, including those in other countries, for research in health and social care. Sharing research data is important to allow peer scrutiny, re-use (and

For peer review only - http://bmjopen.bmj.com/site/about/guidelines.xhtml

therefore avoiding duplication of research) and to understand the bigger picture in particular areas of research. Data sharing in this way is usually anonymised (so that you could not be identified) but if we need to share identifiable information we will seek your consent for this and ensure it is secure. You will be made aware then if the data is to be shared with countries whose data protection laws differ to those of the UK and how we will protect your confidentiality.

Although what you say in the consultations with your stop smoking practitioner is confidential, should you disclose anything to us which we feel puts you or anyone else at any risk, we may feel it necessary to report this to the appropriate persons.

What will happen if I don't want to carry on with the study?

Your participation is voluntary, and you are free to withdraw at any time, without giving any reason, and without your legal rights being affected. If you want to withdraw from the study, you can do so at any time by texting 07537404542, calling us on 0115 7486681, or by emailing snap2study@nottingham.ac.uk

If you withdraw from the study, we will no longer collect any information about you or from you but we will keep the information about you that we have already obtained. This information may have already been used in some analyses and may still be used in the final study analyses. To safeguard your rights, we will use the minimum personally identifiable information possible.

Involvement of the General Practitioner/Family doctor (GP) and hospital

We tell your GP and the hospital where you plan to deliver your baby, that you are taking part in the study and what this involves (with your consent). We will ask your GP if there is any reason why you might not be suitable to take part in this study.

What will happen to any samples I give?

Only members of the research team, relevant regulatory authorities and the University-approved laboratory who test the saliva will have access to the results of your samples. The saliva samples will be tested for the amount of cotinine and / or anabasine in them. Cotinine is a chemical that is produced when nicotine (from cigarettes) is broken down by the body, present in both NRT and tobacco smoke and anabasine is present in tobacco smoke. All samples will be stored in a monitored freezer at the University-approved laboratory that will carry out their testing.

If the saliva sample you provide us is taken by a researcher then they will post this sample to a University-approved laboratory to be tested on the day it was taken from you. We may ask you to post your samples directly to the University-approved laboratory, if samples are taken by yourself at home (using a pre-paid, stamped addressed envelope we will provide you). The sample will have a study number, initials, whether it is your first or subsequent sample, and date of sample, for identification so only the research team will be able to link your sample to you. Once the laboratory has analysed your sample and we have checked the results, the sample will be destroyed in accordance with the Human Tissue Act 2004.

What will happen to the results of the research study?

The results of the study may be presented to other researchers, at conferences and through publication in scientific and medical journals. No names will be used in the results and individuals will not be identifiable in any written reports or presentations. It is also intended that the findings will be used to design new techniques that stop smoking practitioners can use to support women to stop smoking during their pregnancy.

Who is organising and funding the research?

This research is being organised by the University of Nottingham and is being funded the National Institute for Health Research (NIHR), Programmes for Applied Health Research.

For peer review only - http://bmjopen.bmj.com/site/about/guidelines.xhtml

Who has reviewed the study?

All research in the NHS is looked at by an independent group of people, called a Research Ethics Committee, to protect your interests. This study has been reviewed and given favourable opinion by London Bloomsbury Research Ethics Committee.

Further information and contact details

Chief investigator:

Professor Tim Coleman
University of Nottingham
Nottingham
NG7 2RD
Phone: 0115 8230204
Email: tim.coleman@nottingham.ac.uk

Senior Trial Manager:

Miranda Clark
University of Nottingham
Nottingham
NG7 2RD
Email: miranda.clark@nottingham.ac.uk
Telephone: 0115 7486681

General trial:

Email: snap2study@nottingham.ac.uk
Telephone: 0115 7486681

For peer review only

For peer review only - http://bmjopen.bmj.com/site/about/guidelines.xhtml

CONSENT FORM

(Final Version 2.1 12.09.22)

Title of Study: Smoking, Nicotine and Pregnancy 2 Trial (SNAP 2)

Chief Investigator: Professor Tim Coleman
Principal Investigator:
Participant Name:
Participant Number:

Site Number:
REC ID: 21/LO/0123
IRAS ID: 287771

Please initial box

1. I confirm that I have read and understand the Participant Information Leaflet version number 2.1 12.09.22 for the above study and have had the opportunity to ask questions.
2. I understand that my participation is voluntary and that I am free to withdraw at any time, without giving any reason, and without my medical care or legal rights being affected. I understand that should I withdraw then the information collected so far cannot be erased and that this information may still be used in the project analysis.
3. I understand that relevant sections of my medical notes and data collected in the study may be looked at by authorised individuals from the University of Nottingham, the research group (University of East Anglia and University of York) and regulatory authorities where it is relevant to my taking part in this study. I give permission for these individuals, where it is relevant, to collect, store, analyse and publish information obtained from my participation in this study. I understand that my personal details will be kept confidential.
4. I understand and agree that breath samples and/or saliva samples will be requested to measure my carbon monoxide and nicotine levels.
5. I agree to being followed-up by the research team during the study by telephone/video call, text, email, post, or face-to-face appointments.
6. I agree to my GP and hospital where I will deliver my baby being informed of my participation in this study, and to my GP being asked to provide information if there are any reasons that I should not take part.
7. I agree to my mobile phone number being used in this study and consent to it being passed to the University of Cambridge and their nominated text carrier (FastSMS), and/or a different text carrier (Esendex), so that I can receive study related text messages. I understand that my mobile number will only be used for this study.
8. I understand that my anonymised data collected in the study may be used to support other research in the future and may be shared with other researchers.
9. **(Not essential to study participation)** I agree to have my contact details kept after the end of the study (for a maximum of 3 years) so that I can be contacted about the findings of the study and informed of follow-up studies. Yes No
10. **(Not essential to study participation)** I agree that the stop smoking consultation I receive can be audio recorded and that anonymous quotes from the consultation may be used in study reports. Yes No
11. I agree to take part in the above study.

Name of Participant	Date	Signature
Name of Person Taking Consent	Date	Signature

3 copies: 1 for participant, 1 for the project notes and 1 for the medical notes

For peer review only - http://bmjopen.bmj.com/site/about/guidelines.xhtml

Reporting checklist for protocol of a clinical trial.

Based on the SPIRIT guidelines.

Instructions to authors

Complete this checklist by entering the page numbers from your manuscript where readers will find each of the items listed below.

Your article may not currently address all the items on the checklist. Please modify your text to include the missing information. If you are certain that an item does not apply, please write "n/a" and provide a short explanation.

Upload your completed checklist as an extra file when you submit to a journal.

In your methods section, say that you used the SPIRITreporting guidelines, and cite them as:

Chan A-W, Tetzlaff JM, Gøtzsche PC, Altman DG, Mann H, Berlin J, Dickersin K, Hróbjartsson A, Schulz KF, Parulekar WR, Krleža-Jerić K, Laupacis A, Moher D. SPIRIT 2013 Explanation and Elaboration: Guidance for protocols of clinical trials. BMJ. 2013;346:e7586

		Reporting Item	Page Number
**Administrative information**			
Title	#1	Descriptive title identifying the study design, population, interventions, and, if applicable, trial acronym	1
Trial registration	#2a	Trial identifier and registry name. If not yet registered,	2

For peer review only

		name of intended registry	
Trial registration: data set	#2b	All items from the World Health Organization Trial Registration Data Set	na

Fully anonymized trial data will be available upon request from the York Trials Unit

Protocol version	#3	Date and version identifier	2
Funding	#4	Sources and types of financial, material, and other support	21
Roles and responsibilities: contributorship	#5a	Names, affiliations, and roles of protocol contributors	1,20
Roles and responsibilities: sponsor contact information	#5b	Name and contact information for the trial sponsor	21
Roles and responsibilities: sponsor and funder	#5c	Role of study sponsor and funders, if any, in study design; collection, management, analysis, and interpretation of data; writing of the report; and the decision to submit the report for publication, including whether they will have ultimate authority over any of these activities	Suppl. materials
Roles and responsibilities: committees	#5d	Composition, roles, and responsibilities of the coordinating centre, steering committee, endpoint adjudication committee, data management team, and	Suppl. materials

For peer review only

		other individuals or groups overseeing the trial, if applicable (see Item 21a for data monitoring committee)	
**Introduction**			
Background and rationale	#6a	Description of research question and justification for undertaking the trial, including summary of relevant studies (published and unpublished) examining benefits and harms for each intervention	4-5
Background and rationale: choice of comparators	#6b	Explanation for choice of comparators	5
Objectives	#7	Specific objectives or hypotheses	4-5
Trial design	#8	Description of trial design including type of trial (eg, parallel group, crossover, factorial, single group), allocation ratio, and framework (eg, superiority, equivalence, non-inferiority, exploratory)	5
**Methods: Participants, interventions, and outcomes**			
Study setting	#9	Description of study settings (eg, community clinic, academic hospital) and list of countries where data will be collected. Reference to where list of study sites can be obtained	6-7
Eligibility criteria	#10	Inclusion and exclusion criteria for participants. If	6

For peer review only

		applicable, eligibility criteria for study centres and individuals who will perform the interventions (eg, surgeons, psychotherapists)	
Interventions: description	#11a	Interventions for each group with sufficient detail to allow replication, including how and when they will be administered	7-8
Interventions: modifications	#11b	Criteria for discontinuing or modifying allocated interventions for a given trial participant (eg, drug dose change in response to harms, participant request, or improving / worsening disease)	8
Interventions: adherance	#11c	Strategies to improve adherence to intervention protocols, and any procedures for monitoring adherence (eg, drug tablet return; laboratory tests)	8
Interventions: concomitant care	#11d	Relevant concomitant care and interventions that are permitted or prohibited during the trial	7-8
Outcomes	#12	Primary, secondary, and other outcomes, including the specific measurement variable (eg, systolic blood pressure), analysis metric (eg, change from baseline, final value, time to event), method of aggregation (eg, median, proportion), and time point for each outcome. Explanation of the clinical relevance of chosen efficacy and harm outcomes is strongly recommended	13
Participant timeline	#13	Time schedule of enrolment, interventions (including any run-ins and washouts), assessments, and visits for	10-12

For peer review only

		participants. A schematic diagram is highly recommended (see Figure)	
Sample size	#14	Estimated number of participants needed to achieve study objectives and how it was determined, including clinical and statistical assumptions supporting any sample size calculations	13-14
Recruitment	#15	Strategies for achieving adequate participant enrolment to reach target sample size	6-7
**Methods: Assignment of interventions (for controlled trials)**			
Allocation: sequence generation	#16a	Method of generating the allocation sequence (eg, computer-generated random numbers), and list of any factors for stratification. To reduce predictability of a random sequence, details of any planned restriction (eg, blocking) should be provided in a separate document that is unavailable to those who enrol participants or assign interventions	6
Allocation concealment mechanism	#16b	Mechanism of implementing the allocation sequence (eg, central telephone; sequentially numbered, opaque, sealed envelopes), describing any steps to conceal the sequence until interventions are assigned	7
Allocation: implementation	#16c	Who will generate the allocation sequence, who will enrol participants, and who will assign participants to	7

For peer review only

		interventions	
Blinding (masking)	#17a	Who will be blinded after assignment to interventions (eg, trial participants, care providers, outcome assessors, data analysts), and how	7
Blinding (masking): emergency unblinding	#17b	If blinded, circumstances under which unblinding is permissible, and procedure for revealing a participant's allocated intervention during the trial	7
**Methods: Data collection, management, and analysis**			
Data collection plan	#18a	Plans for assessment and collection of outcome, baseline, and other trial data, including any related processes to promote data quality (eg, duplicate measurements, training of assessors) and a description of study instruments (eg, questionnaires, laboratory tests) along with their reliability and validity, if known. Reference to where data collection forms can be found, if not in the protocol	9, 11-12
Data collection plan: retention	#18b	Plans to promote participant retention and complete follow-up, including list of any outcome data to be collected for participants who discontinue or deviate from intervention protocols	12
Data management	#19	Plans for data entry, coding, security, and storage,	12-13

For peer review only

		including any related processes to promote data quality (eg, double data entry; range checks for data values). Reference to where details of data management procedures can be found, if not in the protocol	
Statistics: outcomes	#20a	Statistical methods for analysing primary and secondary outcomes. Reference to where other details of the statistical analysis plan can be found, if not in the protocol	14
Statistics: additional analyses	#20b	Methods for any additional analyses (eg, subgroup and adjusted analyses)	14
Statistics: analysis population and missing data	#20c	Definition of analysis population relating to protocol non-adherence (eg, as randomised analysis), and any statistical methods to handle missing data (eg, multiple imputation)	14

Methods: Monitoring

Data monitoring: formal committee	#21a	Composition of data monitoring committee (DMC); summary of its role and reporting structure; statement of whether it is independent from the sponsor and competing interests; and reference to where further details about its charter can be found, if not in the protocol. Alternatively, an explanation of why a DMC is not needed	n/a

As the trial tests the ability of a behavioural intervention to improve women's use of a standard treatment for smoking cessation, we do not consider that a Data Monitoring Committee (DMC) is required. This trial is not categorised as a CTIMP, so formal adverse event monitoring is not

For peer review only

required.

Data monitoring: interim analysis	#21b	Description of any interim analyses and stopping guidelines, including who will have access to these interim results and make the final decision to terminate the trial	n/a

As this is an evaluation of a behavioural intervention, we do not anticipate there to be a need to discontinue the trial due to adverse outcomes or for any other reasons. Adverse pregnancy outcomes, which are thought to be associated with smoking, will be examined to assess any changes in their prevalence caused by the intervention rather than as an assessment of the intervention itself. In addition, there will be no formal comparative monitoring of accumulating outcome data, and no comparison of any emerging signal with benefit or futility stopping boundaries/rules.

Harms	#22	Plans for collecting, assessing, reporting, and managing solicited and spontaneously reported adverse events and other unintended effects of trial interventions or trial conduct	15
Auditing	#23	Frequency and procedures for auditing trial conduct, if any, and whether the process will be independent from investigators and the sponsor	n/a

There are no plans to audit trial conduct beyond the scope of the data management plan

Ethics and dissemination

Research ethics approval	#24	Plans for seeking research ethics committee / institutional review board (REC / IRB) approval	15

For peer review only

Protocol amendments	#25	Plans for communicating important protocol modifications (eg, changes to eligibility criteria, outcomes, analyses) to relevant parties (eg, investigators, REC / IRBs, trial participants, trial registries, journals, regulators)	15
Consent or assent	#26a	Who will obtain informed consent or assent from potential trial participants or authorised surrogates, and how (see Item 32)	6-7
Consent or assent: ancillary studies	#26b	Additional consent provisions for collection and use of participant data and biological specimens in ancillary studies, if applicable	6-7
Confidentiality	#27	How personal information about potential and enrolled participants will be collected, shared, and maintained in order to protect confidentiality before, during, and after the trial	12
Declaration of interests	#28	Financial and other competing interests for principal investigators for the overall trial and each study site	20
Data access	#29	Statement of who will have access to the final trial dataset, and disclosure of contractual agreements that limit such access for investigators	15
Ancillary and post trial care	#30	Provisions, if any, for ancillary and post-trial care, and for compensation to those who suffer harm from trial participation	8,15
Dissemination policy:	#31a	Plans for investigators and sponsor to communicate trial	15

For peer review only

trial results		results to participants, healthcare professionals, the public, and other relevant groups (eg, via publication, reporting in results databases, or other data sharing arrangements), including any publication restrictions	
Dissemination policy: authorship	#31b	Authorship eligibility guidelines and any intended use of professional writers	15
Dissemination policy: reproducible research	#31c	Plans, if any, for granting public access to the full protocol, participant-level dataset, and statistical code	15

Appendices

Informed consent materials	#32	Model consent form and other related documentation given to participants and authorised surrogates	Suppl. materials
Biological specimens	#33	Plans for collection, laboratory evaluation, and storage of biological specimens for genetic or molecular analysis in the current trial and for future use in ancillary studies, if applicable	13

None The SPIRIT Explanation and Elaboration paper is distributed under the terms of the Creative Commons Attribution License CC-BY-NC. This checklist can be completed online using https://www.goodreports.org/, a tool made by the EQUATOR Network in collaboration with Penelope.ai

For peer review only
