# Supplementary material for: Smoking, nicotine and pregnancy 2 (SNAP2) trial: protocol for a randomised controlled trial of an intervention to improve adherence to nicotine replacement therapy during pregnancy
Source: BMJ Open. 2024 May 28;14(5):e087175. doi: 10.1136/bmjopen-2024-087175 (PMC11138292; doi:10.1136/bmjopen-2024-087175)
Supplement: Reviewer comments [file bmjopen-2024-087175.reviewer_comments.pdf]

PEER REVIEW HISTORY

BMJ Open publishes all reviews undertaken for accepted manuscripts. Reviewers are asked to complete a checklist review form (**http://bmjopen.bmj.com/site/about/resources/checklist.pdf**) and are provided with free text boxes to elaborate on their assessment. These free text comments are reproduced below.

ARTICLE DETAILS

**TITLE (PROVISIONAL)**	Smoking, nicotine and pregnancy 2 (SNAP2) trial: protocol for a randomised controlled trial of an intervention to improve adherence to nicotine replacement therapy in pregnancy
**AUTHORS**	Clark, Miranda; Cooper, Sue; Naughton, Felix; Ussher, Michael; Emery, Joanne; McDaid, Lisa; Thomson, Ross; Phillips, Lucy; Bauld, Linda; Aveyard, Paul; Torgerson, David; Berlin, Ivan; Lewis, Sarah; Parrott, Steve; Hewitt, Catherine; Welch, Charlie; Parkinson, Gill; Dickinson, Anne; Sutton, Stephen; Brimicombe, James; Bowker, Katharine; McEwen, Andrew; Vedhara, Kavita; Coleman, Tim

This article was not externally reviewed at BMJ Open. Protocols that have been independently assessed prior to submission to BMJ Open are usually fast-tracked to publication on the grounds that further substantial changes will not be possible. This independent assessment will usually be external, independent review for both a substantial grant award from a non-commercial or government funder and ethics approval.
